# Photothermally Powered 3D Microgels Mechanically Regulate Mesenchymal Stem Cells Under Anisotropic Force

**DOI:** 10.1002/adma.202506769

**Published:** 2025-09-24

**Authors:** Chen Wang, Nergishan İyisan, Philipp Harder, Valentin H. K. Fell, Viktorija Kozina, Hendrik Dietz, Olivia M. Merkel, Berna Özkale

**Affiliations:** ^1^ Microrobotic Bioengineering Lab School of Computation Information and Technology Department of Electrical Engineering Technical University of Munich (TUM) Hans‐Piloty‐Straße 1 85748 Garching Germany; ^2^ Munich Institute of Robotics and Machine Intelligence Technical University of Munich Georg‐Brauchle‐Ring 60 80992 Munich Germany; ^3^ Munich Institute of Biomedical Engineering Technical University of Munich Boltzmannstraße 11 85748 Garching Germany; ^4^ Pharmaceutical Technology and Biopharmaceutics Department of Pharmacy Ludwig‐Maximilians‐Universität in Munich Butenandtstraße 5‐13 81377 Munich Germany; ^5^ Laboratory for Biomolecular Nanotechnology Department of Biosciences School of Natural Sciences Technical University of Munich Am Coulombwall 4a 85748 Garching Germany

**Keywords:** differentiation, mechanical stimulation, mechanotransduction, nanorobotic microgels, photothermal actuation, spatially patterned forces, stem cells

## Abstract

Exogenous forces significantly influence mammalian cell behavior, yet current strategies fail to resolve signaling processes between individual cells under conditions that accurately mimic the native microenvironment. This work presents a new cell culture technology capable of applying spatially patterned exogenous forces on individual cells within multicellular clusters encased in three‐dimensional (3D) hydrogel matrices. Photothermally powered 3D microgels containing stem cells and integrated force generators are engineered to investigate intercellular communication under anisotropic forces with excellent spatial resolution (≈1 µm). Varying force patterns, such as uniform compression versus spatially heterogeneous tension, are achieved in 3D by relying on the synergistic effect of plasmonic gold nanorods and thermally responsive co‐polymers under light actuation. The microgels generate 17–34 nN force locally, which activates mechanically sensitive ion channels in encapsulated cells stimulated with isotropically applied compression and spatially heterogeneous tension in 3D in a selective manner. Spatially patterned exogenous forces trigger F‐actin remodeling, nuclear translocation of Yes‐associated protein (YAP) and Runt‐related transcription factor 2 (RUNX2) in encapsulated cells following cyclic stimulation. Sustained application of exogenous forces over three days is sufficient to regulate stem cell fate toward osteogenesis. This technology allows combinatorial studies of biomolecular and biophysical cues in 3D, making it suitable for applications in mechanobiology and bioengineering.

## Graphical abstract

The photothermally powered microgel is designed to mechanically train mesenchymal stem cells using spatially patterned exogenous forces in three‐dimensional (3D) workspaces. When microgels are activated selectively via photothermal actuation, locally confined tens of nN forces are exerted on cells, triggering osteogenic differentiation in encapsulated cells.

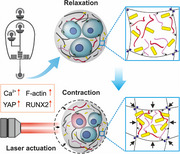

## 1 Introduction

Cells residing in native tissue experience exogenous forces in a periodic manner, which guide changes in cell morphology, migration, proliferation, differentiation, and apoptosis.^[^ ^1^, ^2^, ^3^, ^4^, ^5^, ^6^, ^7^, ^8^ ^]^ Early efforts to decipher how mammalian cells sense, transmit, and interpret these forces heavily relied on established micromanipulation technologies ranging from optical tweezers to micropipette aspiration.^[^ ^9^, ^10^ ^]^ These investigations led to the discovery of key cellular components and pathways that play a role in mechanotransduction, including integrin receptors, mechanosensitive ion channels, and transcriptional regulators such as Yes‐associated protein (YAP) and transcriptional coactivator with PDZ‐binding motif (TAZ).^[^ ^11^, ^12^, ^13^, ^14^, ^15^, ^16^, ^17^, ^18^ ^]^ However, established micromanipulation technologies have commonly suffered from a lack of resemblance to the native tissue microenvironment in terms of biomolecular properties and dimensionality, limiting their use to planar cell culture conditions.

To overcome these limitations, cellular manipulation strategies that integrate exogenous force generation into artificial extracellular matrices (ECMs) that resemble the native microenvironment have been developed.^[^ ^19^, ^20^ ^]^ For example, elastomer‐based stretching devices were used to apply uniaxial tension on large populations of various cell types .^[^ ^21^, ^22^, ^23^, ^24^ ^]^ Subjecting adult stem cells cultured over two‐dimensional (2D) surfaces to cyclic stretching in a directional manner induced their differentiation into contractile cell types, clearly demonstrating the relevance of anisotropic forces in programming cell behavior.^[^ ^21^, ^22^, ^23^ ^]^ Integrating hydrogels with actuation systems allowed force application in 3D while maintaining control over anisotropic force patterns.^[^ ^24^, ^25^, ^26^ ^]^ A significant benefit of this approach is the ability to recreate adaptable force patterns according to the natural requirements of different cell types. For instance, applying directional tension on adipose‐derived stem cells in three‐dimensional (3D) matrices led to myogenic differentiation and muscle fiber formation.^[^ ^25^, ^26^ ^]^ On the other hand, subjecting mesenchymal stem cells to isotropic pressure enabled chondrogenic differentiation and prevention of fibrosis at the cellular level, under biophysical conditions resembling native tissue.^[^ ^27^, ^28^ ^]^ Interestingly, applying directional compression on human mesenchymal stem cells embedded in gelatin‐based matrices led to osteogenic differentiation under periodic stimulation.^[^ ^29^ ^]^ While these examples clearly highlight the relevance of directional force patterns on cell behavior in large populations, the influence of anisotropic versus isotropic mechanical stimuli on individual cells remains elusive.

Introducing small‐scale actuators in artificial ECMs could offer a solution, enabling the generation of exogenous forces with tunable patterns at single‐cell resolution, while maintaining the structure and biomolecular properties of the 3D microenvironment.^[^ ^7^, ^30^, ^31^ ^]^ Cellular networks at different size scales were manipulated in a spatiotemporally controlled manner with magnetically controlled microrobots, which were used to sense cell‐generated traction forces in cancer cell clusters and zebrafish embryos.^[^ ^30^, ^31^, ^32^, ^33^ ^]^ Acoustic tweezers have also been employed to manipulate single cells and molecules in a label‐free manner, applying forces in the sub‐pN to hundreds of pN range, with spatial resolution ≈1–10 µm.^[^ ^34^ ^]^ Similarly, optically triggered small‐scale actuators have been used to generate forces from a few pN up to tens of µN with nanometer resolution.^[^ ^35^, ^36^, ^37^ ^]^ This feature has allowed targeting individual integrins in single cells, although it has only been demonstrated over 2D planar cell culture conditions.^[^ ^37^, ^38^ ^]^ Despite their remarkably fast actuation (∼milliseconds) and high force generation capability, the use of surface‐bound nanoactuators restrict the cells to planar 2D surfaces that do not accurately represent the native 3D microenvironment. In our own previous work, we extended the application of optically triggered nanoactuators into 3D artificial ECMs and demonstrated the relevance of isotropic forces on mechanoresponses from single cells.^[^ ^39^, ^40^ ^]^ However, the actuated microgels we previously engineered suffered from lower mechanical performance in comparison to stand‐alone nanoactuators and high heat output, restricting the application of the technology to only short‐term mechanotransduction experiments.^[^ ^39^ ^]^ As such, the effective integration of such nanoactuators in 3D hydrogels and enabling force generation with tunable spatial patterns remains a challenging task. Mechanically active cell culture systems capable of generating spatially patterned exogenous forces are necessary to understand how forces are transmitted within neighboring cells in a 3D microenvironment. The challenge here is to generate a technology capable of generating anisotropic forces to selectively address single cells in 3D multicellular constructs. Such technology should simultaneously enable control over cell‐ECM adhesion ligands to be able to assess the synergistic influence of biomolecular signals and exogenously applied anisotropic forces for effective regulation of cell fate.

**Figure 1:**
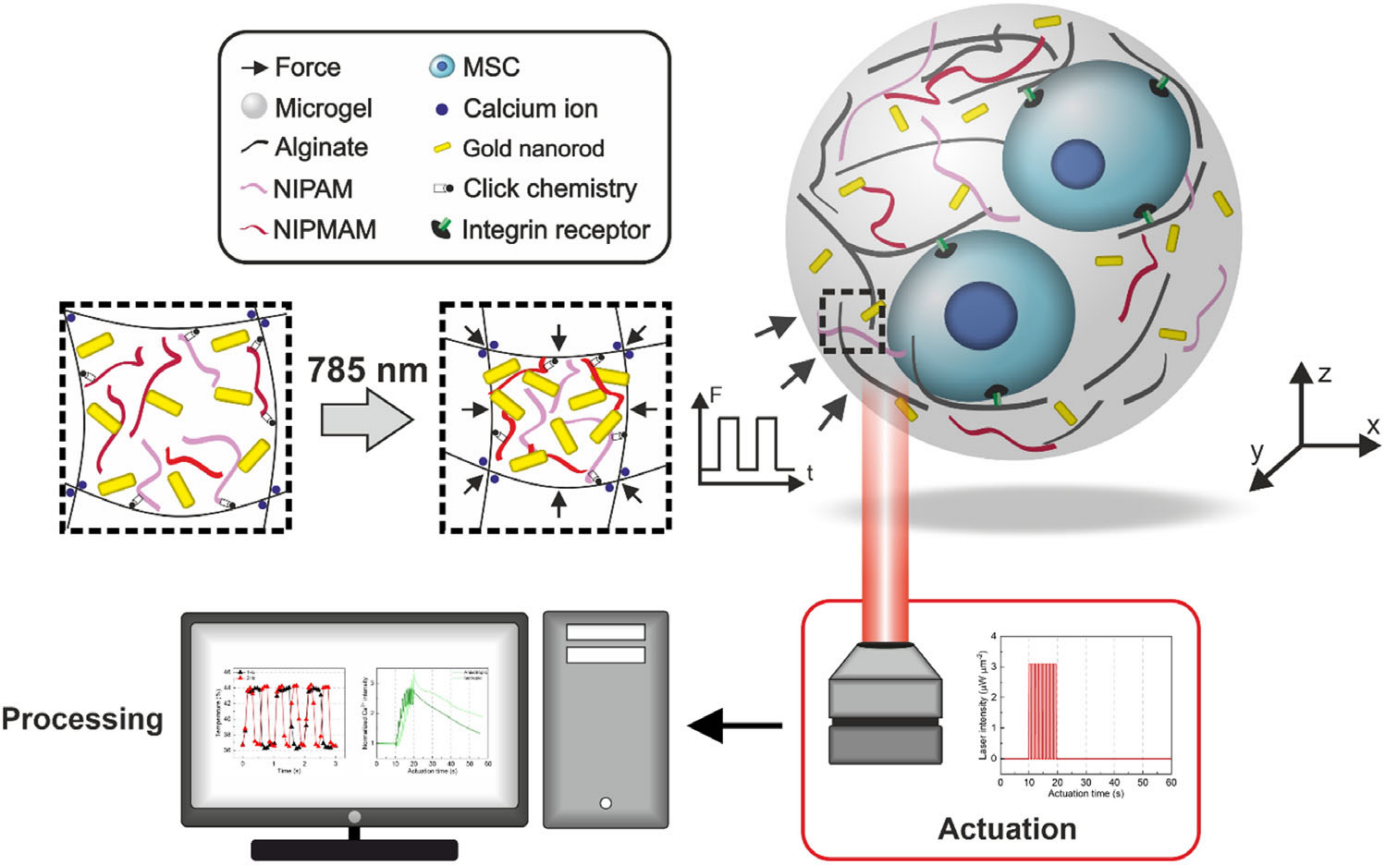
Schematic description of the working principle of photothermally powered 3D microgels. Two mesenchymal stem cells (MSCs) are encased within a microgel consisting of plasmonic gold nanorods and thermoresponsive co‐polymers. The microgel is actuated with a 785 nm near‐infrared laser through an objective in the 3D workspace. Photothermal actuation leads to local contraction of the microgel network, through a combination of plasmonic heating and thermoresponsive phase transition. The system enables control over temperature, strain, and forces with tunable spatial pattern, magnitude, and frequency. The response of individually stimulated MSCs and their influence on neighboring cells that do not receive mechanical actuation is investigated via fluorescence microscopy.

Here, we report on photothermally powered 3D microgels designed to apply spatially patterned exogenous forces on encapsulated mesenchymal stem cells for dynamic investigations on mechanotransduction and cell‐to‐cell communication (**Figure** **1**). We investigate the synergistic influence of cell adhesion in the 3D microenvironment and spatially patterned exogenous forces on the mechanoresponsive behavior of single cells and how this process affects neighboring cells. The microgels consist of thermoresponsive nanoelements and cells within an alginate network, where coupling the thermoresponsive co‐polymers with the plasmonic gold nanorods leads to force generation via light actuation. Our photothermally powered 3D microgels are capable of applying uniform compression (isotropic actuation) and local spatially heterogeneous tension (anisotropic actuation) up to 34 nN, with high precision. We demonstrate that effective force generation and transmission depend on the internal structure of the microgels. To avoid potentially harmful effects of high heat loads on encapsulated cells, the operation temperature of the thermoresponsive copolymer (NIPMAM‐co‐NIPAM) is reduced to a cytocompatible range of 38–42 °C compared to previous work.^[^ ^36^ ^]^ Adjusting the photothermal performance of the microgels by redesigning their architecture and composition leads to radial strains up to 7.2%, which is well above the minimum threshold necessary for mechanosensing.^[^ ^10^ ^]^ Establishing this trade‐off allows force patterning in the microgels, which we use to target distinct regions on the cell membrane of single cells belonging to clusters. Subjecting single cells to localized spatially heterogeneous tension within 3D microgels induces a regional influx of calcs, whereas the calcium intensity rises homogeneously throughout the cells upon uniform compression. We demonstrate that the mechanosensing process and subsequent intracellular calcium signaling are mediated via biomolecular cues in the microenvironment, specifically by integrin‐ECM binding. Prolonging mechanical stimulation leads to increased F‐actin polymerization and nuclear translocation of YAP in encapsulated cells. We finally show that photothermally powered 3D microgels enable regulation of stem cell fate, where sustained actuation over three days trigger osteogenic differentiation.

## 2 Results and Discussion

### 2.1 Synthesis of Thermoresponsive Nanoelements

In this work, our primary goal was to engineer multicellular microgels with integrated force generators to investigate intracellular communication under anisotropic forces. To build our microgels, we chose a combination of thermoresponsive polymers and plasmonic nanoparticles that would generate forces in 3D hydrogel networks. Alginate acted as the static component, providing the 3D microenvironment necessary to culture mesenchymal stem cells. We previously reported on the feasibility of this approach by integrating optically controlled nanoactuators into alginate 3D networks.^[^ ^39^, ^40^ ^]^ While nanoactuators generated forces on the order of several pN, microgel actuation required large heat loads due to the high transition temperature (≈45 °C) of the thermoresponsive polymer. Moreover, a high concentration of nanoactuators was required to trigger effective force generation and transmission within the microgel network, leading to jamming and inhomogeneities during microfluidic production. These shortcomings limited the use of actuated microgels to a handful of short‐range mechanotransduction studies lasting only a few seconds to avoid heat damage to encapsulated cells.

**Figure 2:**
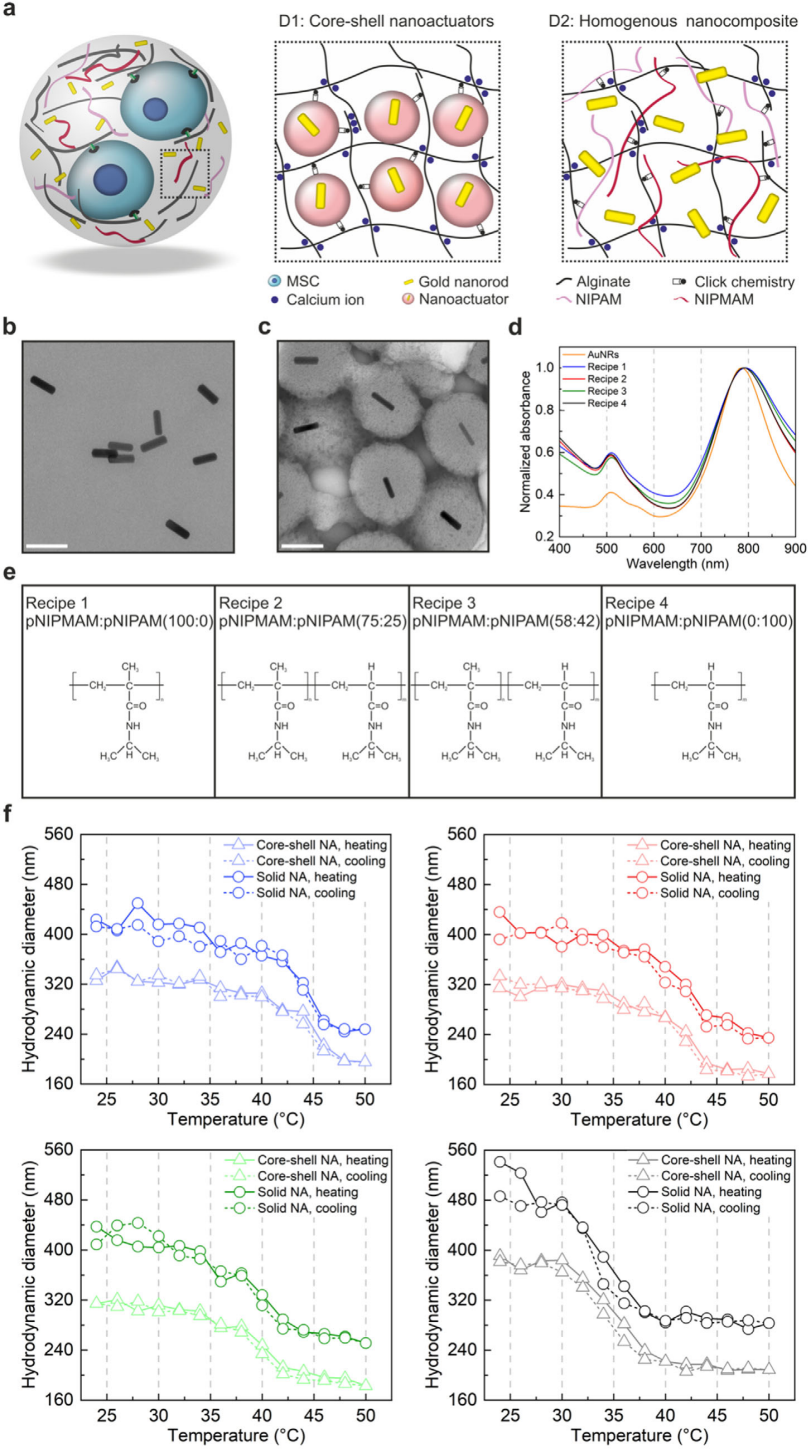
Design and characterization of thermoresponsive nanoelements. a) Schematic overview illustrates two separate designs of thermoresponsive nanoelements, namely (D1) core‐shell nanoactuators, and (D2) homogenous nanocomposite. The core‐shell nanoactuator is composed of a gold nanorod core conformally coated with a thermoresponsive poly(NIPMAM‐co‐NIPAM) co‐polymer shell, which is integrated into an alginate network at 20 mg mL^−1^ final concentration. The homogenous nanocomposite consists of a mixture of homogeneously distributed gold nanorods at 5 mg mL^−1^ concentration with thermoresponsive polymer fragments at a concentration of 12 mg mL^−1^, mixed with alginate biopolymers. For both designs, DBCO‐azide click pair is used to covalently couple the thermoresponsive polymers to the alginate network. Transmission electron microscopy images of b) gold nanorods (scale bar: 200 nm) and c) core‐shell nanoactuators (scale bar: 200 nm). d) UV–vis spectra of gold nanorods and core–shell nanoactuators show the normalized maximum absorbance of gold nanorods at 785 nm before and after polymer coating. e) An overview of the chemical composition of the thermoresponsive co‐polymers, indicating the weight ratios of NIPMAM‐to‐NIPAM used to synthesize the solid and core‐shell nanoactuators. f) The change in hydrodynamic sizes of solid and core‐shell nanoactuators with increasing temperature is shown, measured by DLS. The blue, red, green, and black colors refer to the nanoactuators synthesized via recipe 1, 2, 3 and 4, respectively.

We projected that redesigning the microgel architecture and modulating the actuation temperature toward a cytocompatible range would solve these limitations, enabling long‐range mechanotransduction studies in multicellular microgels. Toward this goal, we proposed two microgel designs to evaluate the influence of microgel architecture on force transmission within the hydrogel network (**Figure** **2**a). Plasmonic nanoparticles and thermoresponsive polymers were integrated into alginate networks either as core‐shell structures (Design 1) or as separate thermoresponsive nanoelements scattered homogeneously within the microgels (Design 2). A major benefit of core‐shell nanoactuators has been high force generation and fast force transmission, owing to the close coupling between plasmonic cores and thermoresponsive polymers.^[^ ^35^, ^36^, ^37^, ^38^, ^39^ ^]^ On the other hand, separating the two thermoresponsive nanoelements (i.e., plasmonic nanoparticles and thermoresponsive polymers) to build homogeneous nanocomposites could allow better regulation of the photothermal performance of the resulting microgels.

In order to produce microgels with either design, we first synthesized the thermoresponsive nanoelements and characterized their properties following a previously established method.^[^ ^39^, ^40^ ^]^ For this purpose, gold nanoparticles were produced via the seed‐mediated nanorod synthesis approach in the presence of silver nitrate. Gold nanorods synthesized this way were either directly used in microgel fabrication or processed further to form core‐shell nanoactuators. In both cases, gold nanorods exhibited the same size (Figure 2b,c), and the optical properties of the plasmonic nanoparticles stayed within the near‐infrared (NIR) range following in situ polymerization (Figure 2d). The gold nanorods were stable in terms of size and surface charges under thermally static conditions, while exhibiting partially reversible agglomerations when subjected to multiple cycles of heating and cooling (Figure S1, Supporting Information). This finding indicated the necessity of stabilizing gold nanorods within the microgel polymer network prior to actuation.

We then focused on lowering the operating temperature of the thermoresponsive nanoelements. To do so, we designed four types of thermoresponsive co‐polymers consisting of varying monomer ratios of *N*‐isopropylmethacrylamide (NIPMAM) to *N*‐isopropylacrylamide (NIPAM), which exhibit high (45–48 °C) and low (32–35 °C) lower critical solution temperatures (LCST) (Figure 2e).^[^ ^41^ ^]^ Using the four different co‐polymers, we fabricated nanoactuators with and without the gold nanorod core, using in situ free radical polymerization (Figure S2, Supporting Information). Polymer growth was achieved in both cases, revealing uniformly formed core‐shell nanoactuators and solid nanoactuators without the gold core (Figure 2c; Figure S3, Supporting Information). Interestingly, core‐shell nanoactuators (351 ± 25 nm) were smaller in terms of average size compared to solid nanoactuators (461 ± 66 nm) evidenced by TEM images and hydrodynamic size measurements (Figure S3, Supporting Information; Figure 2f). This discrepancy may be related to a difference in the reaction efficiency, caused by the presence of competing chemical species brought by the CTAB‐capped gold nanoparticles. Nonetheless, a step‐wise reduction in LCST of the co‐polymer was clearly observed from ≈44 to 34 °C, when changing the respective amounts of high (Recipe 1) and low (Recipe 4) LCST exhibiting monomers used to construct both core‐shell and solid nanoactuators (Figure 2e). We next investigated the mechanical performance of the different nanoactuators to make sure that nanoactuators built with poly(NIPMAM‐co‐NIPAM) co‐polymers retain their deformation ability. Both types of nanoactuators fabricated using all versions of NIPMAM‐NIPAM random co‐polymers exhibited reversible changes in hydrodynamic size exceeding 50% (Figure 2f). We compared the thermomechanical performance among different co‐polymer nanoactuators using the deswelling ratio (*Q_d_*), which was calculated for each type of co‐polymer according to Equation (1),

(1) $$Q_{d}=\frac{r}{r_{0}}$$

where *r* and *r*_0_ represent the radii of nanoactuators at the contracted (at 50 °C) and initial relaxed (24 °C) states, respectively. Regulating the transition temperatures through varying monomer compositions did not dramatically influence the deswelling ratio among the different nanoactuators for neither core‐shell nor solid ones (**Table** **1**). The highest deswelling ratio was observed for nanoactuators containing only pNIPMAM at 0.6 and the lowest was for those consisting of only pNIPAM. These results match well with previously published reports on the tendency for methacrylated co‐polymers of NIPAM to exhibit higher swelling when thermally actuated.^[^ ^41^ ^]^ Despite these observations, the deswelling ratio of nanoactuators was maintained within 0.54–0.6, irrespective of the type of co‐polymer used. A major advantage of this approach is the substantially lowered LCST compared to pure pNIPMAM nanoactuators that operate within a temperature range of 44–48 °C.^[^ ^39^, ^40^ ^]^ Considering the necessity for a cytocompatible actuation temperature, we selected nanoactuators fabricated with Recipe 3‐type co‐polymers, which exhibited a deswelling ratio of 0.58 at a transition temperature of 39 °C, providing a suitable trade‐off (Table 1). Recipe 3‐type solid nanoactuators exhibited excellent stability in terms of size, which stayed constant after multiple heating‐cooling cycles in aqueous solutions (Figure S4, Supporting Information). The change in size and surface charge between 24 and 50 °C was repeatable, indicating the transition of the thermoresponsive co‐polymer from hydrophilic to hydrophobic states (Figure S4, Supporting Information).

**Table 1:** Polymer compositions of core‐shell and solid nanoactuators with the corresponding lower critical solution temperatures (LCST) and deswelling ratios Q_d_. The deswelling ratio of each type of nanoactuator was calculated using Equation 1, where nanoactuator radii at contracted past LCST and initial relaxed states were extracted from the DLS measurements.

Recipe	Design	NIPMAM [mg]	NIPAM [mg]	LCST [°C]	*Q_d_*
1	Core‐shell nanoactuator	600	0	44	0.6
1	Solid nanoactuator	600	0	44	0.59
2	Core‐shell nanoactuator	450	150	41	0.56
2	Solid nanoactuator	450	150	41	0.54
3	Core‐shell nanoactuator	350	250	39	0.58
3	Solid nanoactuator	350	250	39	0.57
4	Core‐shell nanoactuator	0	600	34	0.53
4	Solid nanoactuator	0	600	34	0.52

### 2.2 Fabrication of Photothermally Powered 3D Microgels

**Figure 3:**
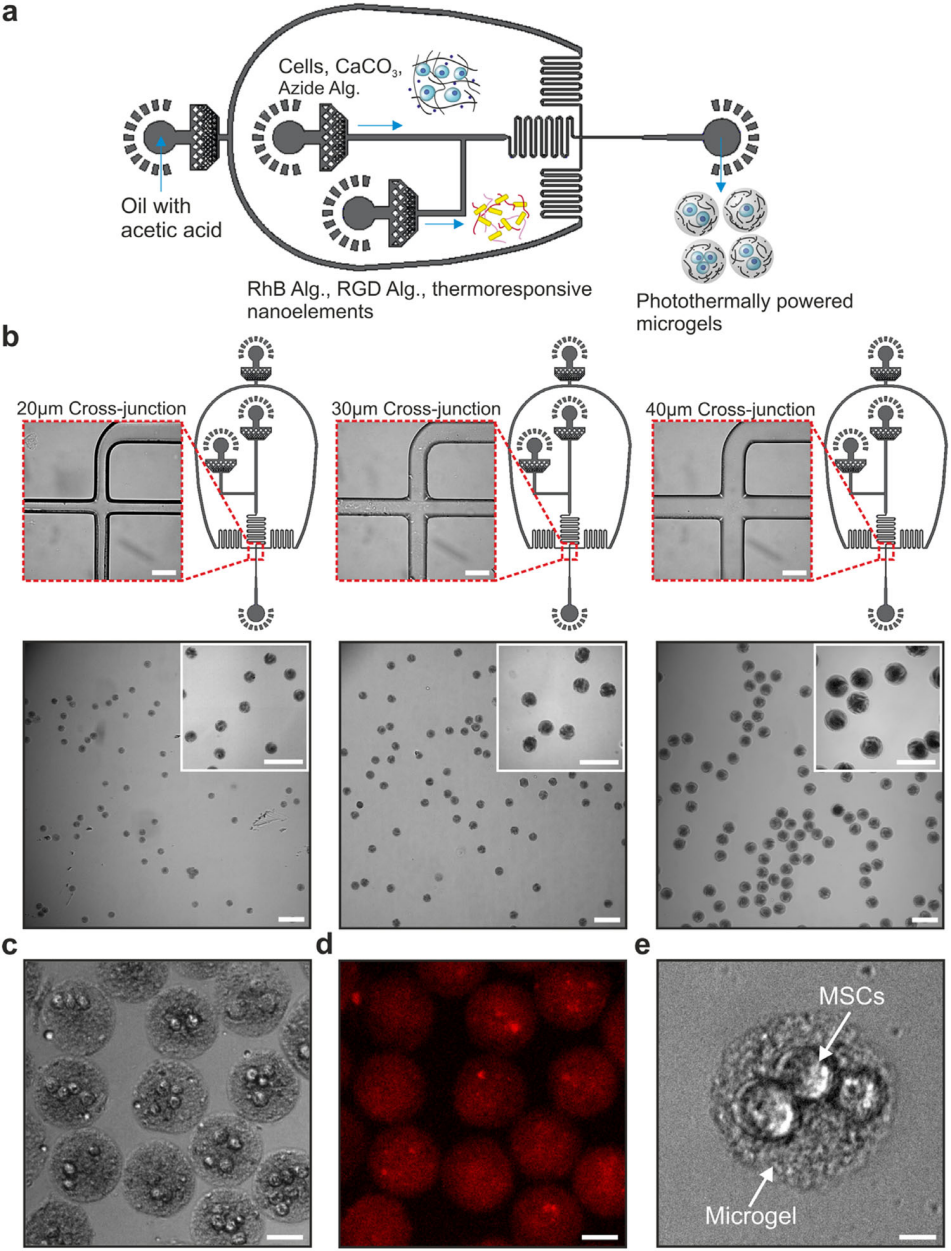
Microfluidic fabrication of photothermally powered 3D microgels with varying sizes. a) A three‐channel cross‐junction microfluidic device enables droplet formation. An intermediate T‐junction is used to mix two aqueous phases carrying (1) CaCO_3_‐treated cells and azide‐modified alginate and (2) a mixture of Rhodamine B‐modified alginate, RGD‐modified alginate, and homogeneous nanocomposite. The homogeneous nanocomposite mixture contains gold nanorods (5 mg mL^−1^) and DBCO‐modified solid nanoactuators (12 mg mL^−1^), subjected to tip sonication prior to microfluidics. Dividing the click pairs into separate aqueous phases prevents premature crosslinking and agglomerations in the channels. All components meet at the T‐junction, which enables proper mixing through the serpentine structure. Droplets are then formed at the cross‐junction where the acetic acid (0.04 vol%) in the oil phase dissolves CaCO_3_ around the cells, thereby crosslinking the alginate network. b) Brightfield images of three different microfluidics designs with 20, 30, and 40 µm channel widths are shown with representative images of cell‐free microgels. Modulating the channel width enables control over microgel size as demonstrated in inset (scale bar: 50 µm) and overview (scale bar: 100 µm) images. c) A representative brightfield image of encapsulated MSCs in photothermally powered 3D microgels (scale bar: 100 µm). d) Corresponding fluorescence image of the same microgels showing the rhodamine B‐labeled alginate network in red (scale bar: 100 µm). e) High‐magnification brightfield image shows an exemplary microgel with three MSCs embedded inside (scale bar: 10 µm).

After obtaining the thermoresponsive nanoelements, we set out to produce photothermally powered 3D microgels using microfluidics (**Figure** **3**a). Our goal here was to uniformly integrate the thermoresponsive nanoelements, e.g., gold nanorods and nanoactuators, into the alginate network, while allowing the encapsulation of multiple cells within the microgels simultaneously. Mechanical coupling between the thermoresponsive nanoelements and the alginate network was achieved using the click pair, dibenzocyclooctyne (DBCO)‐azide, to ensure the efficient transmission of force from the nanoactuators to the alginate network. The presence of rapid binding click pairs in a single aqueous phase could seriously jeopardize the microfluidic process due to agglomerations and channel occlusion. Accordingly, we developed a three‐channel device that would fulfill all of these requirements. We chose to distribute the components making up the aqueous phase into two separate streams, paying attention to the separation of click pairs. It was crucial to sufficiently mix all components within the device prior to droplet formation, to ensure effective control over photothermal actuation in later stages. We evaluated four different geometries combined with a serpentine mixing element for the aqueous phase junction to induce proper mixing of the two aqueous streams (Figure S5, Supporting Information). The shape of the aqueous phase junction and the angle between the two channels influenced the homogeneity of the resulting microgels. Microfluidic devices with a U‐shaped junction produced Janus microgels with two distinctly separate hemispheres, consistent with prior reports.^[^ ^42^ ^]^ Removing the curvature from the two aqueous phase channels induced better mixing of different alginate species in microgels when fabricated with the cross‐ and Y‐shape junctions. However, the distribution of nanoactuators within the microgels was poor despite the presence of mixing units in both cases. Such microfluidic devices have commonly been used to fabricate Janus‐type microgels consisting of two types of biopolymers.^[^ ^42^, ^43^, ^44^ ^]^ In our process, the presence of solid nanoparticles in one of the streams and the necessity to mix all components on chip to form a homogeneous nanocomposite posed a technical challenge. Increasing the angle between the two aqueous streams at the junction site and maintaining a serpentine structure following the T‐junction enabled the fabrication of uniformly mixed nanocomposite microgels (Figure S5, Supporting Information). Microgel uniformity was maintained in T‐junction devices with increasing channel size for all types of nanoactuators being encapsulated (Figure 3b). Based on these findings, we chose T‐junction microfluidic devices with a channel size of 40 µm to encapsulate multiple cells in photothermally‐powered 3D microgels.

Microfluidic cell encapsulation was performed using one aqueous stream to carry the cells, crosslinker CaCO_3_ nanoparticles, and azide‐functionalized alginate (Figure S6, Supporting Information), and a second stream carrying fluorescently tagged alginate, cell adhesive alginate functionalized with RGD peptides, and thermoresponsive nanoelements. Prior to microfluidics, the gold nanorods were mixed with alginate and homogenized via tip sonication, which prevented nanoparticle agglomeration in later stages of fabrication.^[^ ^40^ ^]^ The oil phase included a low concentration of acetic acid, which dissolved the crosslinker particles after droplet formation and induced ionic crosslinking of alginate. We chose Rhodamine B as the fluorescent marker, to localize the microgels during imaging and to enable real‐time monitoring of changes in temperature during photothermal actuation. The separation of click‐modified nanoactuators and the corresponding alginate species prevented premature crosslinking in the device. This fabrication approach was compatible with both types of microgel architecture relying on either core‐shell nanoactuators or homogeneous nanocomposites. Interestingly, microgels containing pure pNIPAM solid nanoactuators exhibited the lowest nanoactuator packing efficiency (Figure S7, Supporting Information) likely due to the difference in surface charges between NIPAM and NIPMAM. Zeta‐potential measurements conducted on the different co‐polymer nanoactuators revealed the highly negative surface charge of pure NIPAM polymer compared to NIPMAM‐NIPAM co‐polymer at 58:42 ratio (Figure S8, Supporting Information). Nonetheless, multiple mesenchymal stem cells (MSCs) were conformally encapsulated in microgels containing thermoresponsive elements in a reliable manner (Figure 3c–e).

### 2.3 Characterization of Thermomechanical Performance

**Figure 4:**
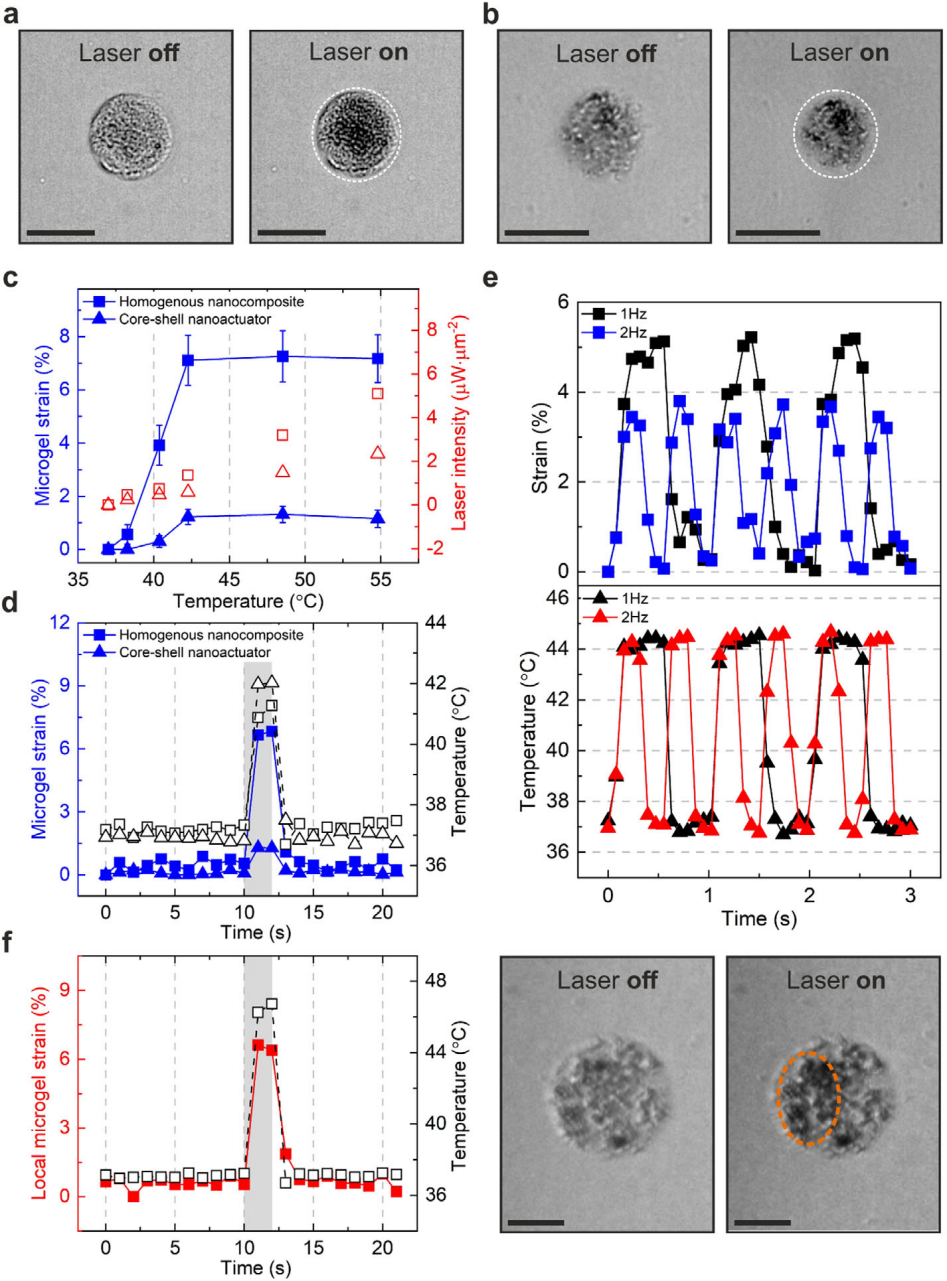
Thermomechanical characterization of photothermally powered 3D microgels. a) Brightfield microscopy images show isotropic actuation of a D1‐type microgels with core‐shell nanoactuators, prior to actuation (laser *off*), and under NIR light actuation (laser *on*) at 2.3 µW µm^−2^ laser power. The dotted white circle indicates the initial perimeter of the microgel before actuation is initiated (scale bar: 40 µm). b) Representative images of a D2‐type microgel with nanocomposite structure are shown during one cycle of isotropic actuation. The dotted white circle indicates the initial circumference of the microgel, clearly showing higher deformation compared to the D1‐type microgel (scale bar: 40 µm). Actuation was performed at 1.4 µW µm^−2^ with a laser on duration of 2 s. c) The comparison of strain performance for D1‐type (core‐shell nanoactuators) and D2‐type (homogeneous nanocomposite) microgels under isotropic actuation is shown. Microgel strain was determined by calculating the average values of radial displacement over the initial radii of microgels (*n* = 10). Microgel temperature was determined for each microgel and condition using Rhodamine B as a heat sensor via the experimentally determined calibration curve, which correlates changes in Rhodamine B intensity to changes in temperature. Triangle and square symbols represent D2‐type microgels with the homogeneous nanocomposite structure and D1‐type microgels with core‐shell nanoactuators, respectively. Blue and red symbol colors refer to microgel strain and laser intensities, respectively. d) Photothermally triggered strain and temperature profiles of a D1‐type and D2‐type microgel are plotted over time. Both microgels were isotropically actuated at 1.4 µW µm^−2^ for 2 s, indicated by the grey bar. e) Frequency modulation of microgel strain over time with corresponding temperature fluctuations of a single D2‐type microgel is shown. The microgel was isotropically actuated at 1.4 µW µm^−2^ laser power with 1 and 2 Hz frequencies with 50% duty cycle. f) Local strain and temperature profiles of an anisotropically actuated microgel are demonstrated with corresponding images of the microgel prior to and during laser activation. The left side (orange dotted ellipse) of the D2‐type microgel with homogeneous nanocomposite structure was actuated at 3.2 µW µm^−2^ for 2 s. In all cases, the concentration of core‐shell nanoactuators in D1‐type microgels was 20 mg mL^−1^, while D2‐type microgels with homogeneous nanocomposite structure had a final concentration of 5 mg mL^−1^ gold nanorods and 12 mg mL^−1^ poly(NIPMAM‐co‐NIPAM) solid nanoactuators (scale bar: 20 µm).

We next characterized the thermomechanical performance of photothermally powered 3D microgels containing either core‐shell nanoactuators (D1) or homogeneous nanocomposites (D2) (**Figure** **4**). The goal here was to identify the microgel architecture necessary to maximize photothermally generated strain at lowest possible heat load. We specifically evaluated the strain performance of D1 and D2 microgels fabricated with Recipe 3 type (LCST = 38–42 °C) thermoresponsive polymers, considering that the actuation temperature had to be higher than 37 °C but still within a tolerable range for cells. NIR laser irradiation led to temporary shrinkage in both microgels as expected (Figure 4a,b), with the deformation more clearly observable in D2‐type microgels possessing the homogeneous nanocomposite structure (Figure 4a). The mechanical performance of D1‐type and D2‐type microgels was compared in a quantitative manner, for which radial strain ɛ_*r*_ (%) was calculated using the following relationship,

(2) $$\varepsilon_{r}\;\left(\%\right)=\frac{r_{0}-r}{r_{0}}\times 100$$

where *r*_0_ and *r* denote the initial and contracted radii of the microgel, respectively. Both types of microgels with core‐shell nanoactuators embedded in an alginate network (D1) and homogeneous nanocomposite structure (D2) exhibited reversible deformation when illuminated isotropically with NIR light (Figure 4c). Here, we used a fluorescence‐based microthermometry approach we developed to track microgel temperature during actuation and correlate laser power to microgel temperature.^[^ ^45^ ^]^ As expected, microgel strain steadily increased with increasing laser power for both types of microgels and plateaued past 42 °C, which was the LCST of the thermoresponsive co‐polymer used (Recipe 3) to construct both types of microgels, consistent with DLS measurements (Figure 2f). Interestingly, D2‐type microgels with homogeneous nanocomposite structure exhibited much higher radial strain (7.2 ± 0.9%) than D1‐type microgels with integrated core‐shell nanoactuators (1.2 ± 0.3%) at 1.4 µW µm^−2^ corresponding to 42 °C (Figure 4c,d). The strain performance of the D1‐type microgels with core‐shell nanoactuators matches well with previous reports on macroscale gelatin matrices comprising optically triggered nanoactuators.^[^ ^7^ ^]^ Our microgels with integrated core‐shell nanoactuators deliver over 1% radial strain at 3 times lower laser power at 13 vol% nanoactuator concentration, compared to macroscale optomechanical gels fabricated at a loading concentration of 20 vol%.^[^ ^7^ ^]^ This observation indicates favorable heat generation and dissipation at small scales, increasing the efficiency of heat transfer and strain generation in the microgels.

When evaluating the influence of microgel architecture on strain performance, we observed that D2‐type microgels with the homogeneous nanocomposite structure generated 7 times higher values than D1‐type microgels despite the higher concentration of poly(NIPMAM‐co‐NIPAM) co‐polymers in core‐shell nanoactuator integrated microgels (20 mg mL^−1^) than in homogeneous nanocomposite microgels (5 mg mL^−1^ gold nanorods and 12 mg mL^−1^ thermoresponsive polymers). The observed difference in strain performance is likely related to the structure of the microgels. Indeed, subjecting a mixture of solid nanoactuators (thermoresponsive polymers without any gold nanorods) and uncrosslinked alginate polymers to tip sonication could have induced much more efficient mixing. In addition, the high concentration of gold nanorods in D1‐type microgels containing core‐shell nanoactuators could have restricted the movement of polymer chains due to their much higher density and tendency to stack. Increasing the concentration of core‐shell nanoactuators in a hydrogel network may lead to undesirable performance loss due to these reasons, although they exhibit excellent thermomechanical coupling between their plasmonic and thermoresponsive components. On the other hand, D1‐type microgels with integrated core‐shell nanoactuators offer higher light‐to‐heat transition efficiency compared to D2‐type homogeneous nanocomposite microgels, evidenced by the lower laser power necessary to induce the same temperature output (Figure 4c). The trade‐off between strain performance and heat output is clearly an important factor to consider when designing actuating microgels for cell applications. Despite its lower light‐to‐heat conversion efficiency, we chose to continue our investigations with D2‐type homogeneous nanocomposite microgels owing to their better strain performance.

Characterizing the temporal strain and temperature profiles of actuated microgels showed fast and reversible network deformation (Figure 4d). Cycling the NIR laser at frequencies ranging from 1 to 4 Hz at 50% duty cycle showed a gradual decrease in the maximum achievable microgel strain, which became much more pronounced beyond 3 Hz (Figure 4e; Figure S9, Supporting Information). Despite these variations in strain behavior, local temperature remained stable at ≈44 °C across all tested frequencies, confirming that the observed lag in strain performance is caused by a delay in the mechanical response of the thermoresponsive polymers. These results also indicate that it may not be possible to reduce output heat loads by simply cycling the laser in this frequency range. We projected that despite a loss in strain amplitude, the ability to modulate the frequency to deliver 4% strain was sufficient for mechanotransduction experiments based on previous reports on macroscale systems.^[^ ^46^, ^47^ ^]^

One of the most important design criteria for photothermally powered microgels was spatial patterning of forces within the microgels. A crucial criterion here was spatial resolution; we therefore evaluated the smallest actuation area and displacement achieved during actuation in 3D microgels. Photothermally powered 3D microgels with integrated fluorescent nanoprobes were imaged at 63X magnification during actuation, where the displacement of the nanoprobes was quantified post‐actuation (Figure S10, Supporting Information). The smallest laser beam size generated at the focal plane was 6 µm in diameter with the current optical stimulation system. The smallest displacement that can be generated under these conditions was 0.2 µm at 3.93 µW µm^−2^ (100 mA) laser power (Figure S10, Supporting Information), which increased with increasing laser power as expected (Figure S10, Supporting Information). These results indicate that the actuation system enables high spatial control over photothermally generated strains within the microgels on the order of 1 µm.

We conjectured that this high spatial resolution would allow force patterning in the microgels. To this end, we evaluated the mechanical performance of microgels under anisotropic actuation (Figure 4f). Subjecting the left hemisphere of a single D2‐type microgel to the NIR laser led to anisotropic deformation of the microgel in the region of interest (Figure 4f, orange ellipse). As expected, the change in local microgel strain over time demonstrated similar temporal response as in the case of isotropically actuated microgels, reaching a maximum of 6.5% when actuated at 3.2 µW µm^−2^. This laser power simultaneously led to a higher output temperature (46 °C) compared to the D2‐type microgel characterized in Figure 4d, which was actuated at 1.4 µW µm^−2^. These results indicate that higher laser power may be necessary for anisotropically actuated microgels to maintain the same level of mechanical output as in the case of isotropically actuated microgels.

### 2.4 Quantification of Force in Actuating Microgels and Assessment of Force Patterns

**Figure 5:**
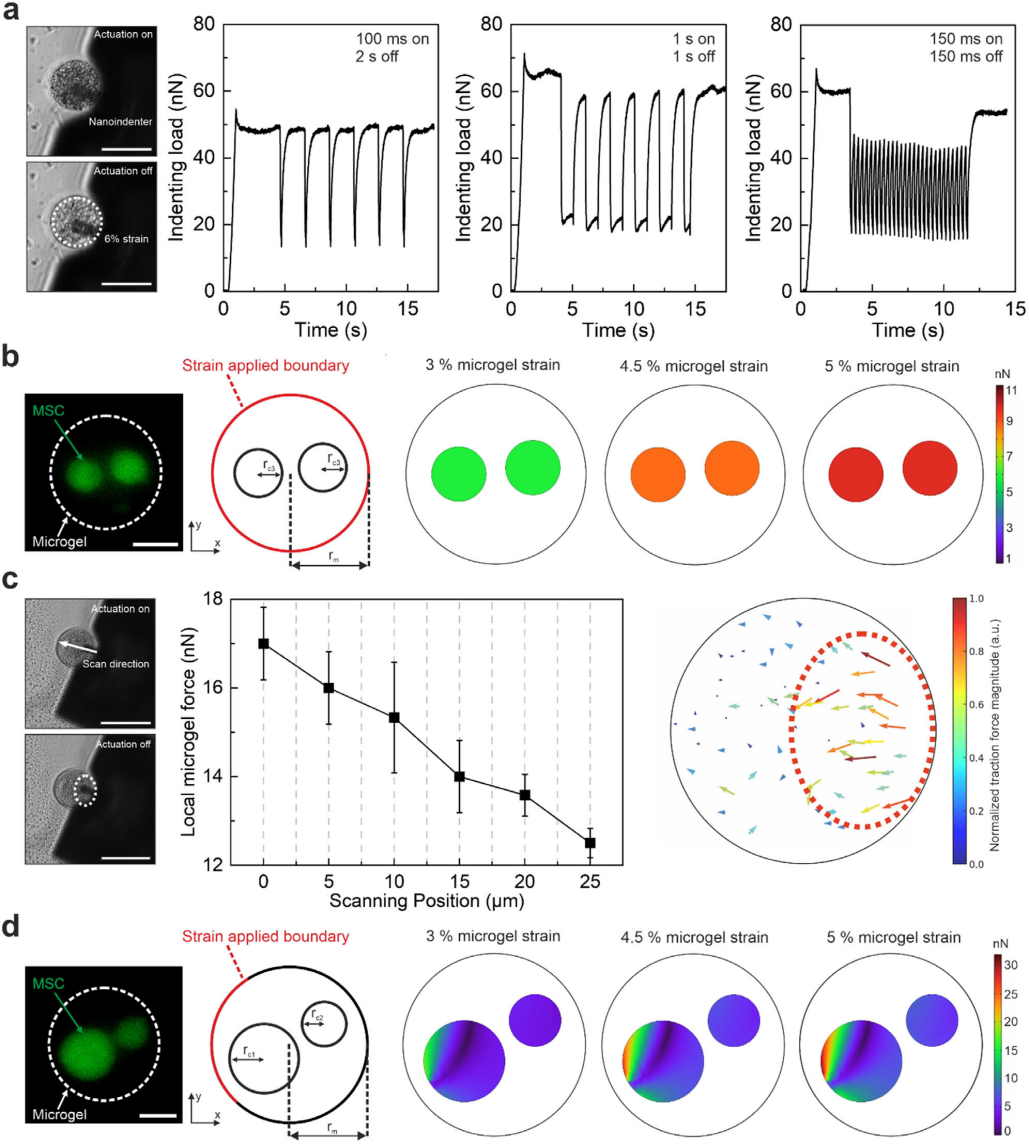
Force quantification in photothermally powered 3D microgels and comparison of force profiles in isotropically and anisotropically actuated D2‐type microgels. a) A single cell‐free microgel is isotropically actuated at 1.4 µW µm^−2^ (white dotted circle) while engaged with the nanoindenter under indenting load (≈50 nN), using different frequencies and duty cycles. The fluctuations in the indenting load over time indicate the force generated by the actuating microgel (scale bar: 30 µm). b) FEM analysis of a multicellular microgel under isotropic actuation at 3%, 4.5%, and 5% strain shows estimated force distribution over the cell surface. Fluorescence image of an exemplary microgel and the corresponding force profiles are shown. The size and location of encapsulated cells were determined from the fluorescence image (scale bar: 20 µm). c) Local microgel force measured over 25 µm distance from the center of actuation in an anisotropically triggered D2‐type microgel with corresponding brightfield images and deformation map (scale bar: 30 µm). Nanoindentation was performed in scanning mode starting from the center of actuation (1.4 µW µm^−2^), designated with the white dotted ellipse. d) FEM analysis of a multicellular microgel under anisotropic actuation, for 3%, 4.5%, and 5% microgel strain (scale bar: 20 µm). All components in the microgels were modeled using elastic solid materials in 2D simulation, where the corresponding mechanical and geometrical properties of the microgels were experimentally determined.

Characterizing the photothermal strain performance of actuating microgels was crucial for evaluating the influence of microgel architecture in a fast and reliable manner. However, accurately determining the force generated within the microgels was necessary in order to precisely regulate the spatiotemporal force patterns applied to encapsulated cells. For this purpose, we investigated the force output of isotropically and anisotropically stimulated single cells using nanoindentation and finite element modeling (**Figure** **5**). We were particularly interested in how locally defined deformation within the microgel network would be transferred to the encapsulated cells and how force distributions varied depending on actuation patterns, i.e., isotropic and anisotropic. Toward this goal, we first evaluated the maximum force generated by a single microgel. A series of nanoindentation measurements was conducted on a microgel, which was isotropically actuated in a cyclic manner with varying laser *on* and *off* durations, while the indenter was engaged with the microgel (Figure 5a). In all cases, the force load curves indicated a decrease in cantilever force during the laser *on* phase, indicating contraction of the microgel. Initial indenting load force was rapidly recovered during the laser *off* phases, corresponding to microgel relaxation. The fast actuation kinetics of our microgels make it an ideal platform to probe both fast and slow cellular responses to exogenous force, such as ion channel activation, cytoskeletal remodeling, and nuclear translocation of mechanosensitive transcription factors. Comparing the force profiles for 100 ms, 150 ms, and 1 s laser *on* durations indicates a fast contractile response on the order of ≈150 ms, while relaxation during the laser *off* phase was slower. The nanoindentation measurements revealed an average force of 34 ± 8 nN taken with a 6 µm round cantilever tip at 3.5 µm indentation depth. When integrated over the entire area of the 40 µm microgel, this value corresponds to a total force of 1.29 ± 0.3 µN per actuating microgel, which is in agreement with earlier reports.^[^ ^35^ ^]^ The ability to tune exogenous forces from tens of nN up to several µNs with µm precision offers a wide range of applications for a variety of mechanosensitive cell types other than stem cells, such as neurons and epithelial cells.^[^ ^19^ ^]^

We next investigated the transmission of the photothermally generated force within the microgel and the magnitude of force acting on encapsulated cells under isotropic actuation. For this purpose, we adapted a finite element modeling (FEM) approach we developed previously using experimentally determined geometrical parameters and mechanical properties of the different components (Table S1, Supporting Information).^[^ ^40^ ^]^ The homogeneous nanocomposite network and the encapsulated cells (Figure 5b) were modeled as linear elastic materials, and experimentally measured microgel strains were assigned as boundary conditions. An exemplary microgel with two encapsulated cells representing the population was chosen when spatially distributing the cells in the model. Under these conditions, the computed force distribution over the encapsulated cell increased with increasing radial strain as expected (Figure 5b), reaching a maximum isotropic force of 9 nN at 5% radial strain. This value is in line with the average force measured via the nanoindenter, which corroborates the reliability of the FEM calculations.

Similarly, we used a combination of nanoindentation and FEM analysis to assess the magnitude of force in anisotropically actuated microgels (Figure 5c,d). The change in nanoindenter load force was recorded over 25 µm from the center of actuation toward the unaffected microgel hemisphere. The local microgel force was recorded to be 17 nN at the center of the actuation area, which steadily decreased as the cantilever was moved away from the region of interest. The deformation profile in the region of actuation indicated a clear difference in spatially heterogeneous tension. Simulations revealed similar results with a maximum force of 15 nN at 3% microgel strain and 25 nN at 5% microgel strain. As expected, these values were recorded over the part of the cell surface closest to the actuation site. The difference in calculated force values between isotropic and anisotropic actuation was likely due to a difference in the boundary conditions. Taken altogether, these results demonstrate that our photothermally powered 3D microgels generate locally defined forces within a range of 17–34 nNs in 3D workspaces. From a biological perspective, this force range is suitable for triggering mechanotransduction through integrin receptors and focal adhesions, which require pN to nN forces.^[^ ^10^ ^]^ The achievable force range in actuating microgels is higher than that of optical and acoustic tweezers, while being lower than the maximum force generated by traditional techniques such as micropipette aspiration, at comparable spatial precision.^[^ ^9^, ^34^, ^48^ ^]^ A major advantage of our system is its high spatial resolution and enhanced addressability in 3D workspaces, which allows the generation of many different force patterns (Figure S11, Supporting Information). These features allow photothermally powered microgels to exceed the capabilities of existing systems (e.g., stretching devices and magnetically controlled microactuators) in terms of the force patterning.

### 2.5 Guiding Mechanotransduction via Optically Patterned Exogenous Forces in 3D Microgels

**Figure 6:**
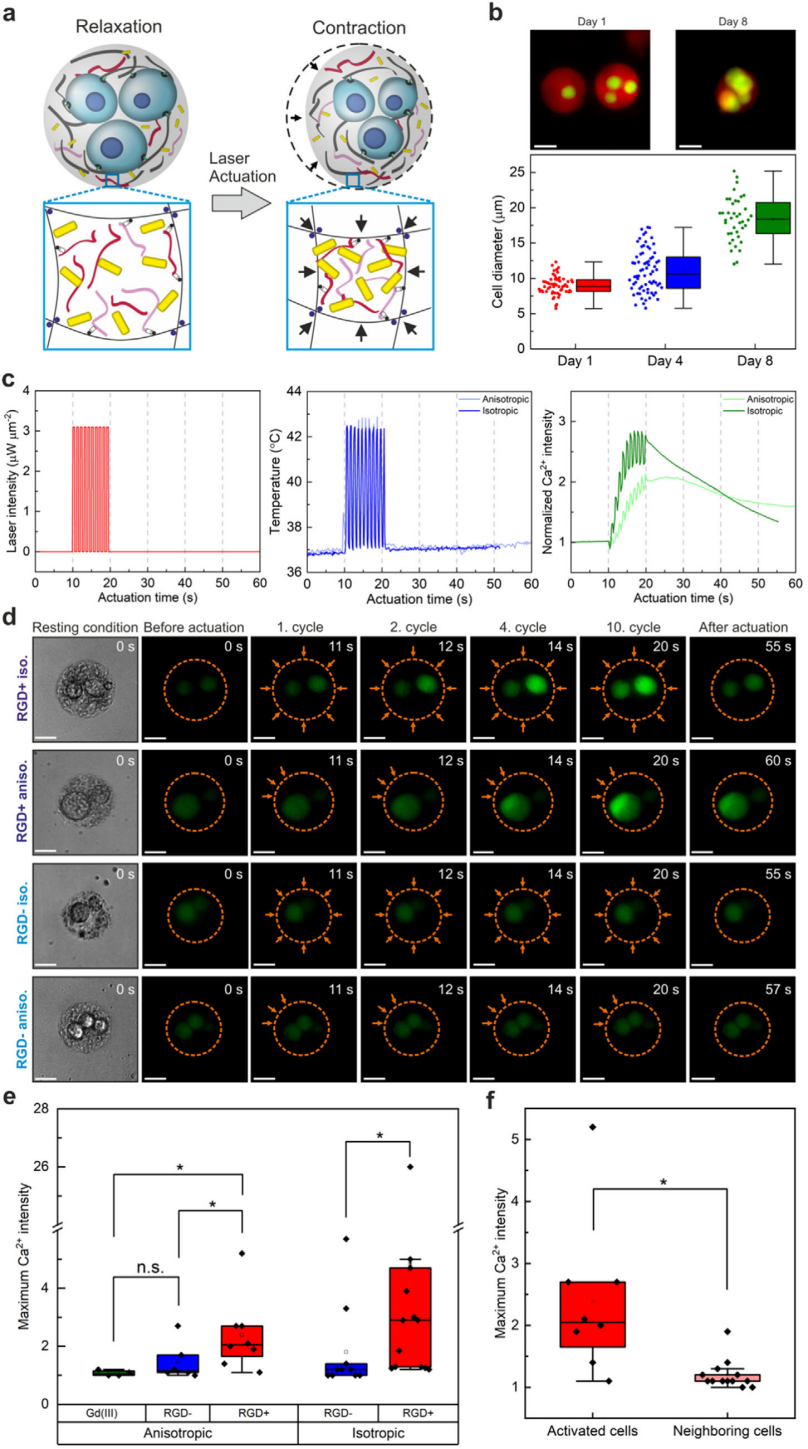
Influence of spatially patterned exogenous forces on cell behavior in multicellular actuating microgels. a) Schematic description of mechanobiology experiments demonstrate partial actuation of stem cell‐encapsulated microgels to apply anisotropic forces in a localized manner. b) Representative fluorescence images of D2‐type actuating microgels carrying multiple MSCs, showing the change in cell size over one week in culture. Red indicates Rhodamine B‐labeled alginate microgel and green shows live cells. The plot shows the change in cell size over 8 days in culture (*n* = 58 for Day 1, *n* = 71 for Day 4, *n* = 40 for Day 8) (scale bar: 20 µm). c) The laser actuation and temperature profiles of two multicellular microgels receiving isotropic and anisotropic actuation are plotted over time. The final plot shows the change in normalized intracellular calcium intensity under isotropic compression and spatially heterogeneous tension. Actuation was initiated at t = 10 s, delivered at 1 Hz frequency (50% duty cycle) using 3.1 µW µm^−2^ laser power for both microgels. Microgels with RGD peptides to enable the mechanical coupling between cells and the ECM were chosen. d) Fluorescence image series showing the change in intracellular calcium intensity of the corresponding cells in the microgels shown in c) before and during actuation in the presence of RGD peptides. Dotted orange lines indicate the perimeter of the microgels, with orange arrows pointing to the site of actuation. The bottom row image series represents intracellular calcium intensities of cells receiving either isotropic compression or spatially heterogeneous tension in actuating microgels in the absence of RGD peptides, revealing a lack of observable response (scale bar: 20 µm). e) Comparative plot showing the maximum fold change in intracellular calcium intensities of cells subjected to anisotropic and isotropic actuation in the presence and absence of cell‐ECM binding RGD peptides. The data for anisotropically actuated microgels only include cells that were directly stimulated (*n* = 8 for RGD peptide‐bearing (RGD+), *n* = 6 for RGD peptide‐absent (RGD‐) microgels). A negative control group treated with Gd^3+^ to block ion channels was included for RGD‐bearing anisotropically actuated microgels (*n* = 4). For the isotropically actuated microgels, all encapsulated cells were included in the quantification (*n* = 14 for RGD peptide‐bearing (RGD+), and *n* = 10 for RGD peptide‐absent (RGD‐) microgels). The fold change in intracellular calcium intensity was determined by normalizing the maximum fluorescence intensity of the calcium dye to the starting baseline conditions (^*^ *p* < 0.05 indicates significant difference, measured using two‐way analysis of variance (ANOVA) followed by Tukey test). f) Comparison of fold‐change in intracellular calcium intensity for actuated and non‐actuated cells within anisotropically stimulated microgels in the presence of RGD peptides. Actuated cells (*n* = 8) were subjected to spatially heterogeneous tension at 1 Hz frequency (50% duty cycle) for 10 s, while the neighboring cells (*n* = 13) were not targeted and therefore did not receive any stimulation (^*^ *p* < 0.05 indicates significant difference, measured using an unpaired two‐sample *T*‐test).

In biological tissues, cells experience a variety of spatially patterned forces, such as tension, compression, and shear stress, which strongly influence cellular processes.^[^ ^3^, ^49^ ^]^ Mimicking these anisotropic forces in vitro may elucidate the principles of mechanotransduction within neighboring cells belonging to the same population. While existing platforms such as micropillar arrays, stretchable membranes, and bioreactors have enabled mechanical stimulation in vitro, they are typically limited to surface‐bound cell culture conditions, can only apply exogenous forces globally, and lack the spatial resolution required to target single cells in 3D.^[^ ^50^ ^]^ To overcome these limitations, we used our platform to study how isotropic and anisotropic force patterns influence the behavior of single encapsulated mesenchymal stem cells (MSCs) and how biological signals are transmitted within neighboring cells (**Figure** **6**a).

Stem cells were encapsulated in photothermally actuated 3D microgels and cultured over 8 days to allow sufficient time for cell‐ECM connections to form (Figure 6b). We specifically chose cross‐junction microfluidic devices with a channel width of 50 µm to enable the encapsulation of multiple cells per microgel. Encapsulated cells remained conformally embedded within the microgels, with the majority of microgels housing multiple cells (Figure 6b; Figure S12, Supporting Information). Over the course of a week in culture, encapsulated cell viability in homogeneous nanocomposite microgels remained above 90% (Figure S12, Supporting Information). This high level of cell viability was maintained during photothermal actuation, even for long periods of actuation reaching 12 h (Figure S13, Supporting Information). To corroborate these findings, we evaluated the impact of heat loads on cell health by investigating apoptosis and proliferation in encapsulated cells subjected to photothermally generated heat loads at 42 °C using 1 Hz frequency and 4.45 µW µm^−2^ laser power for 30 min. The number of apoptotic cells remained constant, below 10%, for isotropically actuated and unactuated control microgels measured at 24 and 72 h after stimulation (Figure S14, Supporting Information). The high percentage of Ki‐67‐positive cells simultaneously indicated proliferative behavior in actuated microgels, confirming that cell health was maintained. Moreover, the expression of heat shock protein 70 stayed constant between actuated and unactuated conditions (Figure S15, Supporting Information), further proving the cytocompatibility of our approach.

Encapsulated MSCs exhibited a clear increase in size throughout the 8 days in culture, nearly doubling in diameter from Day 1 to Day 8, demonstrating that encapsulated MSCs proliferate and adapt within the homogeneous nanocomposite network (Figure 6b). During this time, the overall microgel size did not significantly change, and encapsulated cells stayed intact, indicating the mechanical stability of the photothermally powered microgels carrying cells inside (Figure S16, Supporting Information). Taken together, these results suggest that the microgels support long‐term culture, making them suitable for studies that require long‐term mechanical stimulation, such as lineage commitment studies on a single‐cell level.

We next investigated mechanotransduction of encapsulated MSCs under isotropic and anisotropic force conditions. Intracellular calcium signaling was chosen as a measurable response, due to the mechanosensitive nature of PIEZO and TRPV ion channels in mammalian cells.^[^ ^51^ ^]^ The activation of mechanosensitive ion channels in mammalian cells was demonstrated under 2D culture conditions by locally stretching the cell membrane via patch clamps and microscale force probes.^[^ ^52^, ^53^, ^54^ ^]^ However, only a few studies have been reported on the influence of exogenous forces over calcium signaling in 3D, while there is almost no conceptual evidence on whether spatially patterned forces elicit similar responses.^[^ ^39^, ^55^ ^]^ Moreover, the synergistic influence of biomolecular cues, namely cell adhesion ligand density, and spatially regulated exogenous forces on individual cells in 3D needs addressing. Accordingly, we hypothesized that anisotropic forces could selectively trigger mechanotransduction in encapsulated MSCs in the presence of cell adhesion RGD peptides.

To test this hypothesis, we first applied isotropic actuation on the microgels at 1 Hz at 3.1 µW µm^−2^ and recorded changes in intracellular calcium intensity. The average microgel strain under these conditions was 4%, corresponding to a force of 8 nN, while the output temperature was maintained at 42 °C (Figure 6c; Figure S17, Supporting Information). The decrease in microgel temperature compared to that without cells was most likely related to the displacement of photothermally active material. The isotropically applied exogenous force was sufficient to trigger reversible changes in the intracellular calcium intensity of neighboring cells within the microgels (Figure 6c,d). As expected, the intracellular calcium intensity of two cells sharing the same microgel increased simultaneously in an incremental manner for the entire duration of stimulation (10 s), and decreased to baseline conditions within one  minute post‐actuation (Figure 6d). All actuated cells exhibited reversible changes, with varying rates of intracellular calcium increase within the 10‐s actuation duration (Figure S18, Supporting Information).

One of the strengths of our photothermally powered microgels is its force‐calibrated operation, allowing quantitative investigations on mechanotransduction under exogenous forces. We analyzed the force applied to the encapsulated cells in isotropically actuated microgels to better understand the relationship between exogenous forces and intracellular calcium signaling (Figure S19, Supporting Information). The likelihood of calcium signaling tended to increase above 5 nN of isotropically applied exogenous forces, although no clear correlation was evident between force and maximum change in intracellular calcium content. Cellular heterogeneity likely contributes to the variability in calcium responses among individual cells, as differences in ion channel expression, membrane tension, and cell cycle state may affect the amplitude of calcium signaling, even under comparable force magnitudes. Nonetheless, the increase in intracellular calcium intensity following mechanical stimulation was consistent with prior reports on substrate‐bound cellular networks probed by nanomanipulators.^[^ ^53^, ^54^ ^]^ The force generated by our actuating microgels at 4% strain (≈8 nN) was in close proximity to the force (≈60 nN) reported to trigger intracellular calcium signaling in single osteoblast precursor cells cultured over 2D substrates and probed via nanoindentation at the same actuation frequency (1 Hz).^[^ ^54^ ^]^ While such reports allow qualitative comparison, the lack of single‐cell actuation systems applicable to 3D matrices makes it challenging to make a direct comparison. Our photothermally powered 3D microgels allow single‐cell manipulation in 3D with high spatial precision and control over force parameters.

In comparison, targeting a single cell in multicellular microgels via anisotropic actuation at 1 Hz for 10 s led to responses from only the cells that were selectively stimulated (Figure 6d). The change in intracellular calcium was initiated at the site of actuation, where the maximum applied force was 15 nN, supporting the notion that partial actuation of the cell membrane leads to the activation of calcium signaling via the channels in the same area. Quantifying the average of maximum calcium intensity change for both conditions resulted in a higher fold‐change value for isotropically actuated cells at 4.8 versus anisotropically actuated ones with a value of 2.4 (Figure 6e). Evaluating the applied force and maximum change in intracellular calcium intensity indicated a mechanical threshold (Figure S20, Supporting Information): cells receiving spatially heterogeneous forces less than 5 nN exhibited only minimal change at 10% (*n* = 8). Exogenous forces higher than 5 nN led to a much higher increase in intracellular calcium intensity at an average of 100% (*n* = 11). On the other hand, the average maximum fold change in the intracellular calcium signal of encapsulated cells away from the actuation site within anisotropically triggered microgels was significantly lower than the neighboring cells receiving force application (Figure 6f). This finding demonstrates the capability of photothermally powered microgels to selectively target single cells in multicellular clusters.

The lack of a significant response from neighboring cells residing in anisotropically actuated microgels suggested that the observed signaling processes were actively regulated via mechanically sensitive ion channels. In order to evaluate this claim, we chemically blocked ion channels in actuating microgels using Gd^3+^, following our previously established protocol^[^ ^45^ ^]^ and observed changes in intracellular calcium intensity. In the presence of Gd^3+^, the intracellular calcium intensity remained constant for cells subjected to both types of forces (Figure S21, Supporting Information; Figure 6e).

We next investigated the role of cell‐adhesion ligands in force‐induced calcium signaling with our microgel platform. In the absence of RGD peptides, intracellular calcium signaling was inhibited in both isotropically and anisotropically actuated microgels (Figure 6d), highlighting that effective mechanical signaling requires biomolecular coupling between encapsulated cells and the ECM. This mechanochemical interaction can be precisely studied in our 3D actuating microgel system, compared to conventional 2D stimulation strategies. Tested among a population of cells, the normalized calcium fold change was 1.8 for isotropic and 1.5 for anisotropic force patterns, which are significantly lower than those measured in the presence of RGD peptides. These results suggest that affective force transmission from the microgel network to the cell membrane requires mechanical coupling between actuators and encapsulated cells. The localized spatially heterogeneous tension on the cell adhesion ligands is likely transmitted through the soft cell membrane, triggering the opening of mechanically gated ion channels. Our platform not only offers mechanistic insight into force transmission at the single‐cell level, but also presents a modular interface where alternative ligand chemistries can be readily incorporated to probe diverse receptor‐mediated pathways. Taken altogether, our observations indicate that intracellular calcium signaling is mechanically regulated in photothermally powered 3D microgels. We find that the local force necessary to trigger calcium signaling in 3D confined MSCs is ≈5 nN.

Given that calcium influx is a well‐known activator of pathways that promote focal‐adhesion reinforcement and actin‐filament polymerization,^[^ ^56^ ^]^ we next investigated whether prolonged application of exogenous forces could induce cytoskeletal remodeling in encapsulated MSCs. We were particularly interested in understanding the influence of force magnitude and spatial pattern on the cytoskeletal structure and reorganization in encapsulated MSCs. Stem cells were accordingly encapsulated in RGD‐presenting photothermally powered microgels, considering the importance of cell‐ECM adhesion for mechanotransduction. To ensure optimal transparency for extended imaging durations, we reduced the concentration of gold nanorods to 3 mg mL^−1^. This reduction necessitated an increase in laser power to ensure comparable microgel performance as in intracellular calcium signaling experiments. The photothermally powered microgels carrying multiple cells reached a maximum average temperature of 42.4 ± 1.1 °C (Figure S22, Supporting Information) when actuated at 4.45 µW µm^−2^ laser power, corresponding to 4% radial strain and ≈15 nN of force (Figure S21, Supporting Information).

**Figure 7:**
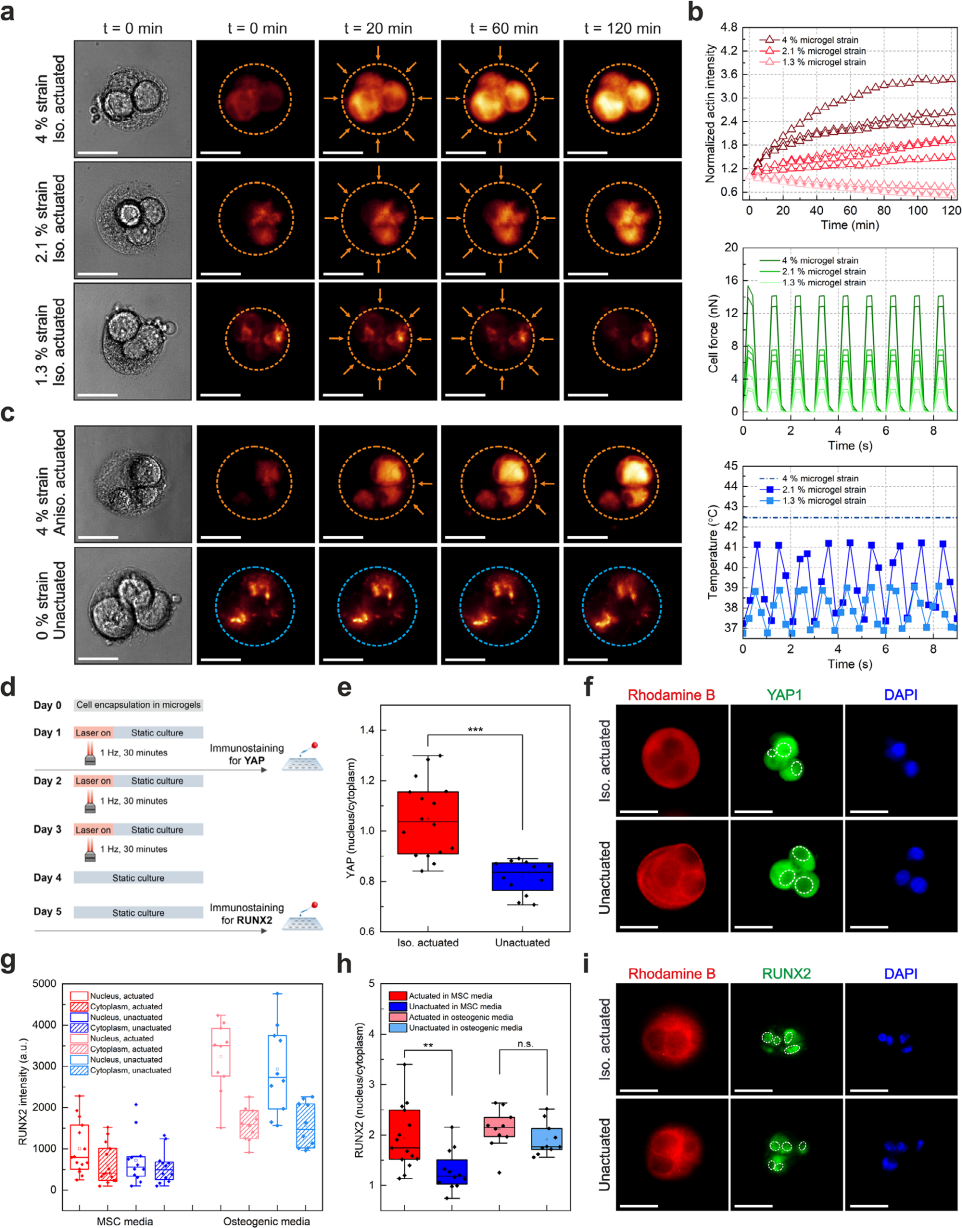
The effect of spatially patterned exogenous forces on actin remodeling, nuclear translocation of YAP, and RUNX2 is investigated using photothermally powered 3D microgels. The microgels contain either 3 mg mL^−1^ or 4.5 mg mL^−1^ of gold nanorods and 12 mg mL^−1^ of NIPMAM‐co‐NIPAM copolymers. a) Fluorescence image series illustrate the change in F‐actin intensity in isotropically actuated microgels for stepwise microgel strains of 4%, 2.1% and 1.3%. Microgels loaded with 3 mg mL^−1^ gold nanorods were actuated with a laser power of 4.45 µW µm^−2^ to enable 4% microgel strain at 1 Hz frequency (50% duty cycle). Lower strain values were achieved in microgels with 4.5 mg mL^−1^ gold nanorod concentration at 2.47 µW µm^−2^ and 3.09 µW µm^−2^ laser power, corresponding to 1.3% and 2.1% strain, respectively. All cells were actuated for 1 h. Dotted orange lines show the perimeter of the microgels subjected to the isotropic actuation, with orange arrows indicating the site of actuation (scale bar: 30 µm). b) The graphs show the change in F‐actin fluorescence intensity, cell force and system temperature of the three corresponding microgels in image series a). c) Fluorescence image series illustrate the change in F‐actin intensity in anisotropically actuated microgels and unactuated control microgels at 4% microgel strain. Dotted orange lines show the perimeter of the microgels subjected to the anisotropic actuation, with orange arrows indicating the site of actuation. Dotted blue lines indicate the perimeter of the control microgels without laser actuation (scale bar: 30 µm). d) Experimental schematic for YAP and RUNX2 translocation assays of cell‐laden microgels. The fabricated microgels were subjected to isotropic actuation at 1 Hz for 30 min, followed by one day of static incubation culture prior to immunostaining. e) The nucleus‐to‐cytoplasm YAP ratio is plotted for isotropically actuated microgels (*n* = 16) versus unactuated control microgels (*n* = 12), both carrying multiple cells inside in MSC media. ^*^ *p* < 0.001 indicates a significant difference, measured using an unpaired two‐sample *T*‐test. The average diameter of cells residing in actuated and control microgels was 19.8 ± 2.5 and 19.9 ± 5.0 µm, respectively. Actuation was performed at a laser power of 4.45 µW µm^−2^ in microgels loaded with 3 mg mL^−1^ gold nanorods at 4% strain. f) Representative fluorescence images of encapsulated cells that received isotropic force stimulation versus those kept under mechanically static, unactuated control conditions. Red indicates the Rhodamine B‐labeled alginate network, while green and blue channels correspond to the YAP signal and DAPI, showing the nuclei of the cells, respectively. White dotted circles show the perimeter of the nuclei for reference (scale bar: 30 µm). g) The absolute RUNX2 intensity in the nucleus and cytoplasm, as well as h) the nucleus‐to‐cytoplasm RUNX2 ratio, are shown for either isotropically actuated microgels (*n* = 15) versus unactuated control microgels (*n* = 12) in MSC media, or isotropically actuated microgels (*n* = 10) versus unactuated control microgels (*n* = 10) in osteogenic induction media. Microgel strain at 4% was achieved at a laser power of 3.45 µW µm^−2^ for RUNX2 measurements, where actuation was conducted at 1 Hz frequency (50% duty cycle) for 30 min per day over 3 days for the RUNX2 translocation study. ^*^ *p* < 0.001 indicates a significant difference, measured using an unpaired two‐sample *T*‐test. i) Representative fluorescence images of encapsulated cells cultured in MSC media, comparing those subjected to isotropic force stimulation with mechanically static, unactuated controls. Red indicates the Rhodamine B‐labeled alginate network, while green and blue channels correspond to the RUNX2 signal and DAPI, showing the nuclei of the cells, respectively. White dotted circles show the perimeter of the nuclei for reference (scale bar: 30 µm).

Subjecting encapsulated cells to periodically applied isotropic forces at 1 Hz frequency for 1 h led to a distinct increase in F‐actin intensity (**Figure** **7**a). The lack of stress fibers was expected considering the round cell shape and the 3D culture environment with an approximate stiffness of 1 kPa.^[^ ^40^ ^]^ We detected over a two‐fold increase in F‐actin intensity of all cells residing in isotropically actuated microgels, where the average applied force was 14 nN (Figures 7; Figure S23, Supporting Information). We next investigated cell response to lower force values, considering the tendency we observed in intracellular calcium signaling. To modulate the magnitude of applied forces, microgel strain was reduced to 2.1% and 1.3% by decreasing the laser power to trigger the thermoresponsive copolymer at local temperatures lower than the LCST (Figure 7b). Encapsulated cells stimulated at 2.1% microgel strain received an average of 6.9 nN, while further reducing the microgel strain to 1.3% led to an average of 3.1 nN of force (Figure 7b). Indeed, reducing the applied force from 14 to 6.9 nN led to reduced F‐actin polymerization, and further decreasing it to 3.1 nN did not induce any changes in F‐actin intensity in the stimulated cells (Figure 7a). The results support the indication of a mechanical threshold at ≈5 nN, below which mechanotransduction is no longer triggered in 3D microgels. Targeting individual cells within multicellular microgels via anisotropic forces with an average magnitude of 15 nN (Figure S23, Supporting Information) led to a selective cell response; only directly stimulated cells exhibited an increase in F‐actin production (Figure 7c; Figure S24, Supporting Information). These observations indicate that the integrin‐dependent force transmission is rapidly translated into cytoskeletal remodeling by triggering F‐actin build‐up.^[^ ^57^, ^58^ ^]^ In contrast, encapsulated cells residing in static microgels without any actuation showed clearly reduced F‐actin production (Figure 7c; Figure S24, Supporting Information). Cells subjected to the laser beam alone without photothermally powered microgels exhibited constant F‐actin intensity (Figure S25, Supporting Information). These findings support the notion that spatially patterned exogenous forces are sufficient to trigger cytoskeletal remodeling in cells encapsulated within 3D microgels as long as the force magnitude is above the mechanical threshold.

We next evaluated whether photothermally applied exogenous forces could influence downstream signaling events in 3D encapsulated cells. For this purpose, we focused our attention on the mechanically sensitive transcriptional regulator YAP in isotropically stimulated cells. Direct force application on mammalian cells cultured over planar substrates has been reported to cause nuclear translocation of YAP.^[^ ^18^ ^]^ We conjectured that periodic application of exogenous forces on encapsulated cells would trigger a similar response. Accordingly, multicellular microgels were isotropically actuated at 1 Hz frequency for 30 min, and the cells were then fixed and stained 24 h post‐actuation (Figure 7d–f). We chose 30 min of isotropic force application, considering the discernible rise in F‐actin intensity in the actuated cells already within the first 20 min of mechanical stimulation. Comparing the 24‐h nuclear‐to‐cytosolic YAP intensity of encapsulated cells receiving isotropic force application demonstrated a significantly higher average value of 1.05, versus unactuated control cells with an average value of 0.83 (Figure 7e). These observations align well with the notion that increased actin polymerization via the integrin–talin–actin axis transmits forces to the nucleus through the LINC complex, where such forces are sufficient to dilate nuclear pores and accelerate YAP nuclear translocation.^[^ ^18^, ^59^, ^60^ ^]^ These results demonstrate that applying exogenous forces of ≈14 nN on stem cells encapsulated within soft microgels enables effective regulation of mechanotransduction. Representative fluorescence images (Figure 7f) visually support this quantitative trend, showing increased nuclear localization of YAP in force‐stimulated cells relative to the static controls. Taken together, our data align with the following cascade: cyclic nN‐scale forces are transmitted through integrin–RGD linkages, leading to mechanosensitive Ca^2^⁺ influx, which, in turn, triggers cytoskeletal reinforcement within tens of minutes, and the resulting increase in actin filaments elevates cortical and perinuclear tension that reaches the nucleus, promoting YAP nuclear import. While this sequence is well established in 2D cells, analogous 3D single‐cell systems remain scarce. Our platform provides a force‐calibrated material system for single‐cell actuation in 3D, with spatial control sufficient to distinguish isotropic from anisotropic responses.

Previous studies demonstrated that mechanical stimulation alone can be an effective strategy to regulate stem cell fate.^[^ ^3^, ^8^, ^10^, ^61^ ^]^ However, the translation of this knowledge to single cells in 3D matrices is lacking. We used photothermally powered 3D microgels to determine if our technology can provide a robust solution. We selected an early osteogenic differentiation marker, RUNX2, as the biological output and investigated its nuclear translocation under mechanical stimulation. Encapsulated cells were isotropically actuated at 1 Hz frequency for 30 min per day, repeated over a three‐day period (Figure 7d). The microgel strain and applied force were kept the same at 4% strain corresponding to 14 nN of average force. Cells were allowed to culture for an additional day, after which they were fixed and stained for RUNX2 at the end of Day 5 (Figure 7d). Stimulated cells showed higher nuclear RUNX2 intensity compared to the cytoplasm (Figure 7g), leading to a significantly higher nuclear‐to‐cytoplasmic ratio of 1.93 relative to the ratio of 1.29 for unactuated control microgels (Figure 7h,i) in regular cell culture media. We then investigated how the presence of osteogenic media would affect cell behavior with and without mechanical stimulation. As expected, encapsulated cells cultured in osteogenic media showed upregulated RUNX2 expression even in the absence of mechanical stimulation (Figure 7g,h). Applying mechanical stimulation under these conditions led to a slight increase in nuclear RUNX2 content, resulting in an elevated nuclear‐to‐cytoplasmic RUNX2 ratio in actuated cells, which was slightly higher than in unstimulated controls. While cells in each group remained similar in terms of size compared to the respective controls, cell morphology was vastly different in the absence and presence of osteogenic media (Figure S26, Supporting Information). Overall, these findings demonstrate the effectiveness of our photothermally powered microgels in regulating cell fate using spatiotemporally controlled exogenous forces.

## 3 Conclusion

Here, we present photothermally powered 3D microgels, designed for high‐resolution multiparametric studies on single‐cell mechanobiology. While established nanomanipulators offer excellent force sensitivity, they are incompatible with the hydrogel matrices necessary to recreate the native 3D microenvironment in vitro. In contrast, our platform enables force actuation within a 3D ECM, matching the force range of traditional techniques while providing 3D culture, comparable spatial precision, excellent temporal control, and the ability to study cells in situ over extended timescales. The actuating microgels are used to apply spatiotemporally controlled exogenous forces on encapsulated stem cells in 3D, relying on the synergistic interaction of plasmonic and thermoresponsive nanoelements. Optical actuation provides excellent micron‐scale resolution (≈1 µm) and millisecond responsiveness (≈100 ms), generating tunable forces within a range of 17–34 nN, well suited for mechanotransduction studies. Our technology makes it possible to quantitatively determine the force necessary to trigger mechanotransduction in 3D, which we demonstrate by investigating intracellular calcium signaling under spatially patterned exogenous forces. Isotropically stimulating mesenchymal stem cells in 3D using a total applied force of 8 nN leads to reversible intracellular calcium signaling in the presence of RGD peptides. This response is inhibited in the absence of cell‐ECM binding, clearly demonstrating the force‐sensing role of integrins and its influence over calcium ion channel activation. Locally stretching a few integrins in the cell membrane of individual cells by applying anisotropic forces at ≈15 nN within multicellular microgels leads to reversible changes in intracellular calcium signals. Remarkably, the platform allows tracking of forces and calcium transients in a spatiotemporally controlled manner at 1 µm resolution. Finally, we show the long‐term actuation capabilities of the photothermally powered 3D microgels by investigating cytoskeletal reorganization and nuclear translocation of the mechanoresponsive transcriptional regulator YAP in encapsulated stem cells. The application of locally defined exogenous forces triggers increased production of F‐actin and nuclear translocation of YAP in encapsulated MSCs above a mechanical threshold of 2.1% microgel strain corresponding to 6.9 nN. While this work focused on integrin‐triggered mechanotransduction, the inclusion of native extracellular matrix molecules will allow investigating the role of other cell adhesion ligands in cell response to exogenous forces. Moreover, we demonstrate the potential of photothermally powered 3D microgels in regulating stem cell fate. Future work will target the conversion of this technology into a high‐throughput single‐cell screening technology. Using the ability to pattern exogenous forces, we aim to control the differentiation of encapsulated MSCs toward osteogenic and chondrogenic differentiation in a rapid manner. This work represents a first step toward training cells through mechanical conditioning, laying the groundwork for future automation and high‐throughput strategies that apply dynamic, programmable force patterns to guide cell fate.

## 4 Experimental Section

### Synthesis of Thermoresponsive Nanoelements

Two types of thermoresponsive nanoelements, namely core–shell nanoactuators and homogenous nanocomposites consisting of gold nanorods and solid nanoactuators were synthesized. Unless otherwise stated, all reagents were purchased from Sigma Aldrich and used without prior purification. Highly pure water (HPW) was obtained by a Sartorius Arium Pro VF Milli‐Q system, Göttingen, Germany. Gold nanorods were first synthesized via seed‐mediated growth method following the previously established protocol.^[^ ^45^ ^]^ Briefly, a growth solution was prepared with two surfactants, namely sodium oleate and hexadecyltrimethylammonium bromide (CTAB), templating molecule silver nitrate (4 mM), metal source gold(III) chloride trihydrate (HAuCl_4_, 1 mM), and ascorbic acid (64 mM) as the reducing agent. The seed solution was prepared by combining 5 mL of 200 mM CTAB and 5 mL of 0.5 mM HAuCl_4_ in a glass scintillation vial. The resulting mixture was stirred at 1200 rpm, resulting in a color change from yellow to orange. A fresh solution of sodium borohydride (10 mM) was prepared, diluted with ultrapure water in a 3:2 ratio, and subsequently added to the CTAB‐HAuCl_4_ solution. This seed solution was stirred at 1200 rpm for 2 min and left undisturbed at room temperature for 30 min to allow aging. Finally, 2.4 mL of ascorbic acid (64 mM) and 320 µL of the seed solution were added to the growth solution in order under 1200 rpm stirring. Following the addition of the seed solution, stirring was stopped and the reaction was allowed to carry on for 15 h at 30 °C, followed by ligand exchange with *N,N'*‐bis(acryloyl)cystamine. The gold nanorods were centrifuged to remove excess reactants and stored at 4 °C until further use.

To synthesize core‐shell nanoactuators, gold nanorods were coated with poly(NIPMAM‐co‐NIPAM) polymers at varying weight ratios denoted as Recipe 1 through 4 (600:0 mg, 450:150 mg, 350:250 mg, 0:600 mg) via in situ free radical polymerization (**Table** **2**). For this, 90 mL of HPW was degassed with N_2_ for 1 h and heated to 70 °C under constant stirring at 700 rpm for 1 h. Respective amounts of *N*‐isopropylmethacrylamide (NIPMAM) and *N*‐isopropylacrylamide (NIPAM) were added, followed by the addition of 60 mg (0.4 mmol) of *N,N'*‐methylenebisacrylamide, which served as the crosslinker. Then, 6 mL of gold nanorod solution was introduced while the stirring speed was increased to 1400 rpm. After 1 min, 480 µL of degassed 0.1 M *2,2′*‐azobis(2‐methylpropionamidine) dihydrochloride (AAPH) was injected into the mixture, and the reaction was stirred for a total of 2 h at 70 °C under continuous N_2_ purging. After 30 min, 60 µL allylamine diluted in 1 mL degassed of HPW was directly added into the mixture, resulting in a turbid solution. The reaction was allowed to proceed under continuous N_2_ degassing and stirring at 1400 rpm for 90 min. Upon completion, the reaction mixture was cooled to room temperature, and the nanoactuators were washed with HPW via centrifugation at 11 000 rpm for three cycles. Solid nanoactuators were synthesized using the same approach with Recipe 1‐4 and by removing gold nanorods from the reaction mixture. All nanoactuators were then washed with ultra‐pure water three times via centrifugation to remove any remaining reactants.

**Table 2:** Amount of reactants in moles and molar concentrations for the four recipes used for synthesizing the thermoresponsive copolymers. In all reactions, the final concentrations of *N,N”*‐methylenebisacrylamide, AAPH, and allylamine were 4 mM, 0.5 mM, and 7.9 mM, respectively. Molar equivalents of the monomers for each recipe were calculated by taking *N,N”*‐methylenebisacrylamide as the limiting reactant.

Recipe	NIPMAM [mg]	NIPMAM [mmol]	NIPMAM [mol eq]	NIPAM [mg]	NIPAM [mmol]	NIPAM [mol eq]
1	600	4.7	11.8	0	0	0
2	450	3.5	8.8	150	1.3	3.3
3	350	2.8	7	250	2.2	5.5
4	0	0	0	600	5.3	13.3

Prior to microfluidic encapsulation, all nanoactuators were functionalized with click moiety dibenzocyclooctyne (DBCO) following the previously established protocol.^[^ ^39^ ^]^ Core–shell and solid nanoactuators were freeze dried for 48 h and resuspended in 0.1 M sodium bicarbonate at a final concentration of 7.5 mg mL^−1^. Resuspended nanoactuators were mixed with dibenzocyclooctyne‐N‐hydroxysuccinimidyl ester (38 mg mL^−1^) dissolved in dimethylformamide (DMF, Tokyo Chemical Industry) in a stepwise manner. The reaction was allowed to take place for 48 h under gentle stirring. Functionalized nanoactuators were washed via three centrifugation cycles at 11 000 rpm and reconstituted to 10 mg mL^−1^. Homogeneous nanocomposites were formed by mixing the gold nanorods and solid nanoactuators with uncrosslinked alginate at final concentrations of 5 mg mL^−1^ and 12 mg mL^−1^, prior to microfluidics. To induce proper mixing, a two‐step sonication (Fisherbrand Model 120 Sonic Dismembrator) approach was used. Modified solid nanoactuators were broken up into polymer fragments via tip sonication for 15 s at 70% amplitude, which was sonicated a second time after mixing it with the gold nanorods.

The synthesized thermoresponsive nanoelements were imaged via transmission electron microscopy (TEM, FEI Tecnai) at 120 kV via negative staining. The detailed TEM preparation protocol is described in the Supporting Information. Nanoactuator size analysis on TEM images was conducted using the open‐source software Fiji (*n* = 100).

### Fabrication of Microfluidic Devices

Microfluidic devices were fabricated via standard soft photolithography based on the previous work.^[^ ^40^, ^45^ ^]^ A detailed description of the fabrication process is provided in the Supporting Information. Briefly, A 4‐inch silicon wafer (p‐type, MicroChemicals GmbH) was spin‐coated with the negative photoresist SU8‐3050 (Kayaku Advanced Materials) at a thickness of 25 µm. UV‐exposure was conducted at 250 µJ cm^−2^ using a tabletop maskless aligner system (µMLA, Heidelberg Instruments). All baking steps and development were conducted following manufacturer instructions. To fabricate the microfluidic devises, a degassed 10:1 mixture of polydimethylsiloxane (PDMS) was poured on the wafer and allowed to cure in an oven at 65 °C for 1 h. The cured PDMS mold was carefully peeled off, and inlets and outlets were created using a biopsy punch. The PDMS mold and glass slide were cleaned via ultrasonication in ethanol and HPW water for 5 min, then dried in the oven for at least 1 h. A compact handheld plasma device (Piezobrush PZ3, Relyon Plasma GmbH) was used to bond PDMS mold to the glass slide by temporally oxidizing their surfaces for 2 min.

### Production of Cell‐Free Photothermally Powered Microgels

Two types of photothermally powered alginate microgels, incorporating either homogenous nanocomposite or core‐shell nanoactuators but without encapsulated cells, were fabricated using microfluidic approach to analyze strain behavior. For this purpose, three functionalized alginate (I‐1G, KIMICA) species were prepared, chemically modifying alginate batches with either 11‐azido‐3,6,9‐trioxaundecan‐1‐amine, Rhodamine B (DS 2), or cell‐binding RGD peptide (Gly)4‐Arg‐Gly‐Asp‐Ser‐Pro (RGD, Peptide 2.0), via EDC‐NHS carbodiimide chemistry. Functionalized alginate solutions were purified via dialysis against decreasing concentrations of sodium chloride for 3 days, and then treated with activated charcoal to eliminate residual impurities. The purified alginate solutions were then filtered through a 0.22 µm membrane, lyophilized at −50 °C for one week using a benchtop freeze dryer, and stored for further use.

For the encapsulation of homogeneous nanocomposites, two separate aqueous phases were prepared. The first phase contained 60 µL of azide‐modified alginate (2 wt%), 40 µL of calcium carbonate nanoparticles (10 mg mL^−1^), and 20 µL of bead buffer (130 mM NaCl, 25 mM HEPES, 2 mM CaCl_2_). The second phase consisted of 60 µL of Rhodamine B‐modified alginate (2 wt%), 50 µL of DBCO‐modified thermoresponsive polymer fragments (12 mg mL^−1^), and 10 µL of gold nanorods (5 mg mL^−1^). Both phases were loaded into separate 0.5 mL Luer‐Lok syringes. A fluorinated oil phase was prepared by mixing HFE 7500 (Novec 750 engineered fluid, 3M) with 1 vol% fluorinated surfactant Pico‐Surf (Dolomite Microfluidics) and 0.04 vol% acetic acid. This oil phase was loaded into a 3 mL syringe. The aqueous and oil phases were injected into a cross‐junction microfluidic device connected to a syringe pump (Darwin Microfluidics) for controlled droplet formation. After collection, emulsions were washed three times with perfluorooctane (PFO) to remove residual oil. Core‐shell nanoactuator‐laden microgels were fabricated using the same microfluidic approach by incorporating core‐shell nanoactuators at a final concentration of 20 mg mL^−1^, while keeping all other components unchanged.

### Strain Characterization of Thermoresponsive Nanoelements and Photothermally Powered Microgels

The strain behavior of thermoresponsive nanoelements was characterized via dynamic light scattering (DLS, Litesizer 500, Anton Paar), over a temperature range of 24 °C to 50 °C. Measurements were conducted in 2 °C increments, with a 3‐min equilibration period at each step. Strain performance of photothermally powered microgels was characterized using a custom set‐up, consisting of an inverted fluorescence microscope (DMi8, Leica Microsystems) equipped with an externally controlled 785 nm laser diode (200 mW, Thorlabs). Microgels were exposed to laser intensities ranging from 0 to 5.1 µW µm^−2^, and the changes in radial strain of microgels were captured via brightfield microscopy. Each photothermal actuation cycle consisted of 2 s of laser exposure followed by a 20‐s relaxation period. The laser intensities were determined by measuring power output using a photometer (Thorlabs) and a photosensitive fluorescent dye, Indocyanine Green, over the effective beam area. The frequency‐dependent strain response of microgels was evaluated by applying laser pulses at 1, 2, 3, and 4 Hz, with 50% duty cycle. Spatially heterogeneous tension refers to microgels that were anisotropically actuated, while isotropic compression was used to define isotropically actuated microgels. To quantify the local temperatures of microgels, the change in Rhodamine B intensities was recorded and converted to temperature values using a previously established calibration curve.^[^ ^40^ ^]^ Briefly, mean normalized Rhodamine B intensity values of the microgels were measured directly in the Leica software and converted into temperature values using the following experimentally determined equation (Equation 3),

(3)

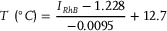

where *I_RhB_* represents mean normalized Rhodamine B intensity from the region of interest, i.e., radial microgel area, and *T* is the corresponding temperature. The LED power and exposure duration were kept constant for calibration and sample measurements. All strain measurements were conducted at a starting temperature of 37 °C by placing the microgels in a stage‐top incubator (H301‐K‐FRAME, Okolab).

### Finite Element Simulation

The numerical simulations were conducted using COMSOL Multiphysics, with version 6.0 for stationary modeling and version 6.2 for time‐dependent simulations, based on the previously published framework.^[^ ^40^ ^]^ The model was designed under the assumption that the mechanical effects of thermoresponsive nanoelements were accounted for by incorporating experimentally measured microgel strains as input. Consequently, the geometries of thermoresponsive polymer fragments were omitted in this 2D simulation. The cells and microgels were modeled as linear elastic solids based on Duhamel–Hooke's theory. The Young's moduli were experimentally measured via a nanoindentation approach, while Poisson's ratio was set to 0.49 for all material components. The geometrical properties including microgel and cell sizes as well as cell positions within microgels were obtained from experimentally acquired microscope images and analyzed using open‐source Fiji software.

### Nanoindentation‐Based Force Quantification

A microscope‐compatible stage‐top nanoindenter (Chiaro, Optics11 Life) was used to characterize the mechanical properties of microgels. The system was equipped with a 3 µm diameter spherical probe tip and a calibrated spring constant of 0.54 N m^−1^. Indentation experiments were performed under controlled conditions, with an indentation depth of 1000 nm, a loading rate of 1250 nm s^−1^, and a hold time of 20 s before retraction. To ensure microgel immobilization during indentation, a poly‐L‐lysine (PLL) (Sigma‐Aldrich) coating was applied to the well plate surface. A 0.05 mg mL^−1^ PLL solution was prepared, and 3 µL was added to each well, followed by incubation at 35 °C for 30 min to facilitate adsorption. After incubation, excess PLL was removed, and microgels were seeded into the wells in cell culture medium, allowing them to settle prior to nanoindentation. To evaluate the mechanical response under optically induced actuation, a 785 nm laser was applied during the hold phase of the indentation cycle. Actuation was performed under two conditions: isotropic (uniform exposure) and anisotropic (localized exposure). In the isotropic condition, the entire microgel was illuminated with 70 mA laser power, and the frequency was varied across three settings: 0.476 Hz (100 ms on, 2 s off), 0.5 Hz (1 s on, 1 s off), and 3.33 Hz (150 ms on, 150 ms off). For anisotropic actuation, only a portion of the gel was exposed to the laser, applying 70 mA at 0.33 Hz (2 s on, 1 s off). The microgel was scanned diagonally with a step size of 5 µm, with each step held for 10 s to assess anisotropic deformation. Scanning began at the laser actuation site, and the laser was used throughout the scanning experiment. Force‐indentation curves were analyzed using the Optics11 Data Viewer software, and all experiments were conducted at room temperature.

### Cell Culture and Microfluidic Cell Encapsulation

Murine mesenchymal stem cells (D1 ORL UVA, ATCC) were cultured in the media containing 10% fetal bovine serum (Gibco, US), high‐glucose Dulbecco's modified Eagle serum (Gibco, US), with 1% penicillin/streptomycin (Gibco, US) at sub‐confluency. Prior to encapsulation, microfluidic devices were sterilized in 70% ethanol and completely dried under sterile conditions. Microfluidic cell encapsulation was performed based on a previously established protocol with modifications. Unlike cell‐free microgel fabrication, the following preparation and procedures were conducted inside a biosafety cabinet to ensure sterile conditions. Two aqueous phases were prepared, which separated azide‐modified alginate and DBCO‐modified nanoactuators. The first aqueous mixture consisted of 60 µL of azide‐modified alginate 2 wt% in ethylenediaminetetraacetic acid, 4‐(2‐hydroxyethyl)‐1‐piperazineetha nesulfonic acid, Dulbecco's Modified Eagle Medium (EDTA–HEPES–DMEM), and 60 µL of a cell suspension containing 12 µL of OptiPrep and 48 µL of CaCO_3_‐treated cells. CaCO_3_‐treated cells were prepared by trypsinizing and centrifuging the cells with CaCO_3_ nanoparticles (CalEssence 70 PCC) to facilitate nanoparticle distribution on the cell surface based on a previously established protocol.^[^ ^39^, ^55^ ^]^ Specifically, cells cultured in a 500 mL standard tissue culture flask were trypsinized and passed through a 40‐µm mesh filter to remove cell aggregates. The resulting cell suspension was adjusted to a concentration of 10^7^ cells mL^−1^ in HEPES‐DMEM (DMEM supplemented with 10% FBS, 1% penicillin‐streptomycin, and buffered with 25 mM HEPES) by centrifugation. To facilitate nanoparticle adsorption on the cell surfaces, 600 µL of the prepared cell suspension was mixed thoroughly with 50 µL of CaCO_3_ nanoparticles by manually pipetting, and the treated cell solution was placed on ice for 5 min. This mixture was then centrifuged at 100 rcf for 5 min and resuspended to promote nanoparticle adhesion on cells. Following a second centrifugation, the cells were resuspended in 48 µL of EDTA‐HEPES‐DMEM (HEPES–DMEM supplemented with 1.8 mM EDTA). The final cell suspension was combined with OptiPrep and azide‐modified alginate as described above and loaded into a 1.5 mL syringe for subsequent use. The second mixture included 40 µL of either RGD‐modified alginate (2 wt%, DS 20) to form RGD‐bearing microgels or unmodified alginate (2 wt%) to create RGD‐absent microgels, 20 µL of Rhodamine B‐modified alginate (2 wt%), 50 µL of DBCO‐modified thermoresponsive polymer fragments (12 mg mL^−1^), and 10 µL of gold nanorods (5 mg mL^−1^). The final concentration of RGD‐modified alginate in cell‐adhesive microgels was 1 wt% at 3.3 degree of substitution (DS). The concentration of gold nanorods was kept at 5 mg mL^−1^ when preparing the encapsulated cells for intracellular calcium signaling studies. The gold nanorod concentration was reduced to 3 mg mL^−1^ for experiments related to cytoskeleton (F‐actin) remodeling and nuclear YAP translocation, to ensure sample transparency during imaging. All other parameters were kept the same. This second mixture was homogenized using tip sonication for 15 s and gently loaded into a 1.5 mL syringe. The oil phase was prepared as previously described, consisting of fluorinated oil HFE 7500 mixed with 1 vol% fluorinated surfactant Pico‐Surf and 0.04 vol% acetic acid. The prepared oil phase was added to a separate 3 mL syringe. Microfluidic encapsulation was carried out by mounting the prepared syringes onto a syringe pump, operated at a flow rate of 1.7 mL min^−1^, under gentle agitation at 350 rpm. The collected emulsions were incubated on ice for 15 min before demulsification with sterile‐filtered 33% PFO in oil. For demulsification, residual oil was first removed from the collected emulsions by pipetting. Then, 1 mL of cell culture media was added, followed by the addition of 220 µL of sterile‐filtered 33% PFO. The mixture was gently shaken up and down 10 times to ensure uniform mixing, then left undisturbed on ice for 5 min. The samples were subsequently washed three times by centrifugation at 270 rcf for 5 min to remove oil and PFO residues. The cell‐encapsulated microgels were cultured over the course of a week at 37 °C, with culture medium exchanged every two days.

### Cell Staining and Imaging

The viability of encapsulated mesenchymal stem cells was assessed using live‐dead cell staining. Encapsulated mesenchymal stem cells were incubated with 2 µM calcein AM and 4 µM ethidium homodimer‐1 (Thermo Fisher Scientific) for 10 min before imaging.

The change in intracellular calcium intensity was tracked using a live‐cell imaging fluorescent dye, Calbryte 520 AM (AAT Bioquest), during laser actuation. Briefly, cells were washed three times with 310 mOsm isotonic solution via centrifugation at 1200 rpm, then they were incubated with 4.5 µM Calbryte solution for 45 min. The stained cells in microgels were washed again using isotonic solution three times. In a separate experimental condition, 150 µM gadolinium (III) chloride hexahydrate was introduced to a subset of microgels to block ion channels. For photothermal actuation, cell‐laden microgels were either fully illuminated to induce isotropic actuation or partially activated to apply anisotropic endogenous forces. Microgels were subjected to cyclic laser beam at 1 Hz and with a 50% duty cycle for 10 s, followed by a 50 s relaxation period. The mean intracellular calcium fluorescence intensity was recorded at an excitation wavelength of 475 nm and quantified via Leica Microsystems Suite X software. The background noise was subtracted from the recorded mean intensities, which were normalized to the initial intensity at t = 0.

The F‐actin in actuated and unactuated control cells was tracked using a live‐cell actin imaging dye (CellMask Green Actin Tracking Stain, Thermo Fisher Scientific). Prior to staining, photothermally powered microgels carrying multiple cells were adhered onto a 96‐well plate, to enable easy tracking in later stages. For this purpose, the substrate was coated with a cationic polymer, poly‐L‐lysine (pLL, 150000–300000 Da, Sigma Aldrich) by adding 30 µL of 0.005% pLL into a 96‐well plate, and drying it completely at 70 °C using a hot plate for 1 h. The cell‐bearing microgels were added to the pLL‐coated well and allowed to adhere for 2 min. The seeded microgels were then washed with cell culture media twice to remove any unattached microgels. A working solution of F‐actin dye was prepared by adding 2 µL of the stain into 1 mL of cell culture media. 100 µL of this solution was then added to the plate seeded with microgels, and incubation was allowed to proceed at 37 °C for 45 min. The working solution was exchanged with fresh cell culture media prior to imaging. To generate 4% microgel strain, actuation was performed at 4.45 µW µm^−2^ with 1 Hz laser frequency (50% duty cycle) for 1 h, while 3.09 and 2.47 µW µm^−2^ were used to achieve 2.1% and 1.3% microgel strain, respectively. The changes in F‐actin intensity for all cases were recorded at 5‐min intervals.

YAP and RUNX2 immunostaining was conducted by following a previously established protocol.^[^ ^55^ ^]^ Cell‐carrying microgels were first placed into 96‐well plates using pLL‐coated substrates as described above. For YAP translocation, actuation was performed on adherent microgels at 1 Hz frequency (50% duty cycle) at 4.45 µW µm^−2^ laser power for 30 min. For RUNX2 translocation, adherent microgels were actuated at 1 Hz (50% duty cycle) with a laser power of 3.45 µW µm^−2^ for 30 min per day over 3 days. For both cases, the microgel strain was maintained at 4%, corresponding to an average force of 14 nN. In the case of RUNX2, actuation was performed in the absence and presence of an osteogenic induction medium (Gibco StemPro Osteogenesis Differentiation Kit, Thermo Fisher Scientific). Following fixation, the encapsulated cells were left in the incubator overnight, and immunostaining was performed 24 h following actuation. For this purpose, encapsulated cells were fixed with 4% paraformaldehyde (Thermo Fisher Scientific) in a buffer solution containing 130 mM NaCl, 2 mM CaCl_2_, and 25 mM HEPES, referred to as bead buffer. Permeabilization of fixed cells was done using 0.3% Triton X‐100 (Thermo Fisher Scientific) in bead buffer. To block non‐specific binding, samples were incubated with 10% goat serum (Thermo Fisher Scientific) for 1 h at room temperature. Cells were then incubated overnight at 4 °C in a humidified chamber with either YAP1 Rabbit Polyclonal Antibody (Invitrogen #PA1‐46189) diluted 1:100 in bead buffer for YAP translocation, or RUNX2 Rabbit Polyclonal Antibody (Invitrogen, #PA5‐86506) diluted 1:100 in 2% goat serum for RUNX2 translocation. The following day, samples utilized for YAP translocation were incubated with a secondary anti‐rabbit antibody conjugated to Alexa Fluor 647 (Abcam, ab150079) at a 1:200 dilution for 1 h at room temperature and Phalloidin‐Alexa Fluor 488 (Invitrogen A12379) at a 1:1000 dilution. Similarly, samples utilized for RUNX2 translocation were incubated with a secondary anti‐rabbit antibody conjugated to Alexa Fluor 488 at a 1:200 dilution for 1 h at room temperature. Nuclear counterstaining was performed using DAPI (1 µg/mL in PBS) for 15 min at room temperature, protected from light, followed by two washes with bead buffer. Fluorescence imaging was performed using the Leica Microsystems Suite X software. To analyze fluorescence YAP and RUNX2 intensity in the cells, nuclear regions were defined based on DAPI staining, and an equivalent cytoplasmic region was selected per cell. Mean YAP and RUNX2 fluorescence intensity was measured in both regions, and the nuclear‐to‐cytoplasmic ratio was calculated to evaluate mechanotransduction in actuated versus control conditions for each cell.

The materials and methods used in apoptosis, proliferation, and heat shock assays are provided in the Supporting Information.

### Statistical Analysis

Unless specified otherwise, all values are presented as means ± standard deviation from at least three measurements. Statistical comparisons between samples were performed using a two‐sample Student's t‐test after confirming normal distribution. To assess statistical significance between group means, a one‐way ANOVA followed by Tukey's post hoc test was applied. P‐values below 0.05 (*), 0.01 (**), or 0.001 (***) were considered statistically significant. Outliers were included in the dataset. Graphs were generated, and statistical analyses were conducted using Origin 2021b.

## Conflict of Interest

The authors declare no conflict of interest.

## Author Contributions

C.W., N.İ., and P.H. conducted experiments and analyzed the data. B.Ö. designed the study. V.F. contributed to the synthesis of thermoresponsive polymers, and V.K. conducted TEM imaging. C.W., N.İ., and B.Ö. wrote the manuscript with contributions from P.H., V.H.K.F., H.D., and O.M.M. All authors have given approval to the final version of the manuscript.

## Supporting information



Supporting Information

## Data Availability

The data that support the findings of this study are available from the corresponding author upon reasonable request.
