# Supplementary material for: Photothermally Powered 3D Microgels Mechanically Regulate Mesenchymal Stem Cells Under Anisotropic Force
Source: Adv Mater. 2025 Sep 24;38(2):e06769. doi: 10.1002/adma.202506769 (PMC12783973; doi:10.1002/adma.202506769)
Supplement: Supplementary file 1 — Supporting Information [file ADMA-38-e06769-s001.docx]

Supporting Information

Photothermally powered 3D microgels mechanically regulate mesenchymal stem cells under anisotropic force

Chen Wang, Nergishan İyisan, Philipp Harder, Valentin H. K. Fell, Viktorija Kozina, Hendrik Dietz, Olivia M. Merkel, Berna Özkale ^*^

Supporting Information

Chen Wang, Nergishan İyisan, Philipp Harder, Valentin H. K. Fell, Viktorija Kozina, Hendrik Dietz, Olivia M. Merkel, Berna Özkale ^*^

C. Wang, N. İyisan, P. Harder, B. Özkale

Microrobotic Bioengineering Lab, School of Computation, Information, and Technology, Department of Electrical Engineering, Technical University of Munich (TUM), Hans-Piloty-Straße 1, Garching 85748, Germany

C. Wang, N. İyisan, P. Harder, B. Özkale

Munich Institute of Robotics and Machine Intelligence, Technical University of Munich, Georg-Brauchle-Ring 60, 80992 Munich, Germany

C. Wang, N. İyisan, P. Harder, B. Özkale

Munich Institute of Biomedical Engineering, Technical University of Munich, Boltzmannstraße 11, 85748 Garching, Germany

V. H. K. Fell, O. M. Merkel

Pharmaceutical Technology and Biopharmaceutics, Department of Pharmacy, Ludwig-

Maximilians-Universität in Munich, Butenandtstraße 5-13, 81377 Munich, Germany

V. Kozina, H. Dietz

Laboratory for Biomolecular Nanotechnology, Department of Biosciences, School of Natural Sciences and Munich Institute of Biomedical Engineering, Technical University of Munich, Am Coulombwall 4a, 85748 Garching, Germany

E-mail: berna.oezkale@tum.de

# Supplementary Methods

**Microfluidic chip fabrication**

The masters of microfluidic devices were produced using single-side polished silicon wafers (4-inch, p-type, MicroChemicals GmbH) via standard photolithography. The silicon wafers were first cleaned in sequence with acetone, isopropanol, and deionized (DI) water to remove any particulates, and pre-baked on the hotplate at 100 °C to remove residual moisture. The substrate was allowed to cool down to room temperature for 5 minutes, and a negative photoresist (SU-8 3050, Kayaku Advanced Materials) was spin-coated onto the wafers. Spin coating was performed in two steps: first at 500 rpm (ramp rate: 100 rpm/s⁻¹) for 10 seconds, followed by 4000 rpm (ramp rate: 300 rpm/s⁻¹) for 30 seconds, yielding a resist layer thickness of approximately 25 µm. The coated wafers were then soft-baked at 95 °C for 15 minutes and allowed to rest at room temperature for 10 minutes to relieve internal stress prior to UV exposure. UV exposure was performed at a dose of 250 μJ cm^-2^ using a tabletop maskless aligner system (µMLA, Heidelberg Instruments). Following exposure, the wafer was post-baked at 68 °C for 2 minutes, then ramped to 95 °C over 15 minutes, and subsequently baked at 95 °C for 5 minutes. The wafer was then allowed to rest at room temperature for 10 minutes to relieve internal stress. Development of the photoresist on the master mold was carried out in three steps: first in mr-Dev 600 for 5 minutes, followed by a brief rinse in isopropanol for 10 seconds, and finally, a second development step in mr-Dev 600 for 5 seconds to optimize development efficiency. The fully developed master was then rinsed with isopropanol, dried with an air gun, and stored in a wafer carrier protected by aluminum foil.

Polydimethylsiloxane (PDMS) pre-polymer and curing agent were mixed at a 10:1 weight ratio and stirred for at least 3 minutes to ensure thorough mixing. The mixture was then poured onto the SU8-master and degassed in a desiccator to remove air bubbles before curing. The mold was subsequently soft-baked at 65 °C for 1 hour. After curing, the PDMS replica was carefully peeled from the master mold, and inlet/outlet holes were created using a 0.8 mm-diameter biopsy punch. For device assembly, both the PDMS molds and glass slides were sequentially cleaned with ethanol and DI water via ultrasonication for 5 minutes, followed by drying in an oven for at least 1 hour. Residual particulates on the PDMS surfaces were mechanically removed using adhesive tapes. A handheld plasma device (Piezobrush® PZ3, Relyon Plasma GmbH) was used to activate the surfaces by temporary oxidation for 2 minutes, enabling irreversible bonding between the PDMS and glass slides. The bonded devices were then baked at 65 °C for 1 hour to enhance adhesion. To render the microchannels hydrophobic, Rain-X was introduced through the inlets. The treated devices were subsequently baked overnight at 65 °C to complete the surface modification.

**Apoptosis and proliferation assay**

Apoptotic DNA fragmentation was assessed using the Click-iT™ Plus TUNEL assay with Alexa Fluor™ picolyl azide dyes (Thermo Fisher Scientific), following the manufacturer’s protocol with minor modifications for adherent cells cultured in well plates. Cells were fixed with 4% paraformaldehyde (PFA) for 20 minutes at room temperature, washed, and permeabilized using 0.25% Triton™ X-100 in bead buffer (130 mM NaCl, 2 mM CaCl_2_, and 25 mM HEPES) for 10 minutes. After two washes with DI water, cells were incubated with TdT Reaction Buffer alone for 10 minutes at room temperature to equilibrate DNA ends prior to labeling.

The TdT reaction mixture was freshly prepared by combining TdT Reaction Buffer, EdUTP, and TdT enzyme, and was added directly to the wells. Cells were then incubated at 37°C for 60 minutes in a humidified chamber to facilitate enzymatic incorporation of EdUTP at DNA strand breaks. Following the reaction, wells were washed twice with 3% bovine serum albumin (BSA) in PBS. Fluorescent labeling of incorporated EdUTP was performed via copper-catalyzed azide-alkyne cycloaddition by preparing the Click-iT™ Plus TUNEL reaction cocktail. This cocktail consisted of the Click-iT™ Plus Supermix (containing the Alexa Fluor™ picolyl azide dye) and the 10x Reaction Buffer Additive. The additive was reconstituted by dissolving the lyophilized powder in 2 mL of DI water to generate a 100x stock, which was diluted 1:10 immediately before use. The final cocktail was added to the wells and incubated for 30 minutes at 37°C in the dark.

After labeling, cells were washed with 3% BSA in bead buffer. For co-staining of proliferative cells, samples were blocked with 3% BSA in bead buffer for 1 hour at room temperature to prevent non-specific binding, then incubated with Ki-67 Recombinant Rabbit Monoclonal Antibody (SP6) (Invitrogen #MA5-14520, 1:100 dilution) overnight at 4°C in a humidified chamber. The following day, cells were incubated with an Alexa Fluor 488-conjugated secondary antibody (Abcam, ab150077) for 1 hour at room temperature. Nuclear counterstaining was performed using DAPI (1 µg/mL in PBS) for 15 minutes at room temperature, protected from light, followed by two bead buffer washes. Fluorescence imaging was conducted using Leica Microsystems Suite X software, with excitation settings of 647 nm for TUNEL and 488 nm for Ki-67. Quantification was performed by measuring fluorescence intensity and calculating the percentage of TUNEL- and Ki-67-positive cells.

**Heat shock assay**

Antibody staining was performed to assess heat shock protein 70 (Hsp70) expression to evaluate cellular heat response. Briefly, encapsulated cells were fixed with 4% paraformaldehyde (Thermo Fisher Scientific) in bead buffer, followed by permeabilization with 0.3% Triton X-100 (Thermo Fisher Scientific) in bead buffer. Samples were incubated with 10% goat serum (Thermo Fisher Scientific) for 1 hour at room temperature, in order to block non-specific binding. Treated cells were incubated overnight at 4°C in a humidified chamber with Hsp70 Polyclonal Antibody (Invitrogen #10995-1-AP) at a dilution of 1:100 in bead buffer. Secondary antibody conjugation was done the following day by incubating the samples with a secondary anti-rabbit antibody conjugated to Alexa Fluor 647 (Abcam, ab150079) at a dilution of 1:200 for 1 hour at room temperature and Phalloidin-Alexa Fluor 488 (Invitrogen A12379) at a dilution of 1:1000 in bead buffer. For nuclear counterstaining, DAPI was used, and cells were incubated in 1 µg/mL DAPI in PBS for 15 minutes in the dark at room temperature, followed by two washes in bead buffer. Fluorescence imaging was conducted using the Thunder Leica microscope, and image analysis was performed using the Leica Microsystems Suite X software. The background-subtracted fluorescence intensities for Hsp70 were quantified and compared between control and actuated cell groups.

**Transmission Electron Microscopy on Thermoresponsive Nanoelements**

FCF-400-Cu TEM with a Formvar carbon film grid was purchased from Electron Microscopy Sciences, which was glow-discharged for 45 s at 35 mA before incubating the synthesized thermoresponsive nanoelements on it for 60 to 120 s, depending on the sample concentration. The grids were then stained with 2% uranyl formate containing 25 mM NaOH for 30 s and subsequently blotted dry on filter paper. Imaging was performed at magnifications ranging from 42000x to 110000x, using FEI Tecnai T12 electron microscope at 120 kV and Tietz TEMCAM-F416 camera, operated with the SerialEM software.

**Evaluation of Mixing Performance among Varying Microfluidic Designs**

Our purpose was to generate microgels with a homogenous distribution of multiple components within the alginate network. To this end, we designed a series of three-channel flow-focusing microfluidic devices with diverse internal structures and compared their mixing performance. For the first aqueous phase, 60 µL core-shell recipe 1-based nanoactuators (10 mg mL^-1^) with 60 µL Rhodamine B-tagged alginate (2 wt%) were used. For the second aqueous phase, 60 µL unmodified alginate (2 wt%), 40 µL calcium nanoparticles (10 mg mL^-1^), and 20 µL bead buffer were prepared. The oil phase contained fluorinated surfactant (1 vol%) and acetic acid (0.04 vol%) in HFE 7500. The microfluidic fabrication was performed at 1.7 min mL^-1^, and the emulsion was collected separately from individual devices. The collections were washed with PFO at the end of fabrication and re-dispersed in bead buffer for brightfield imaging analysis.

To validate the brightfield imaging results mentioned above, another microfluidic production was further performed using Fluorescein and Rhodamine B-modified alginates. One mixture contained 60 µL Rhodamine B modified alginate (2 wt%), 40 µL calcium carbonate, and 20 µL bead buffer, while another mixture was prepared with 60 µL Fluorescein modified alginate (2 wt%), 40 µL calcium carbonate, and 20 µL bead buffer. The Fluorescein signal was recorded at 475 nm excitation wavelength and filtered through a 535 nm bandpass, whereas Rhodamine was imaged with 555 nm excitation wavelength and filtered through a 590 nm bandpass. Images extracted from both fluorescent channels were merged via Leica Microsystem Suite X software.

Supplementary Tables

Table S1. Parameters utilized in mechanical simulation modeling.

| Mechanical properties |  |  |
| --- | --- | --- |
| Name | Young's modulus / kPa | Poisson's ratio |
| Cell | 1.41 | 0.49 |
| Microgel | 1.24 | 0.49 |
| Geometrical properties |  |  |
| Name | Symbol | Value |
| First cell radius | $r_{c1}$ | 12.5 µm |
| Second cell radius | $r_{c2}$ | 8 µm |
| Third cell radius | $r_{c3}$ | 8.5 µm |
| Microgel radius | $r_{m}$ | 28 µm |

# Supplementary Figures

**
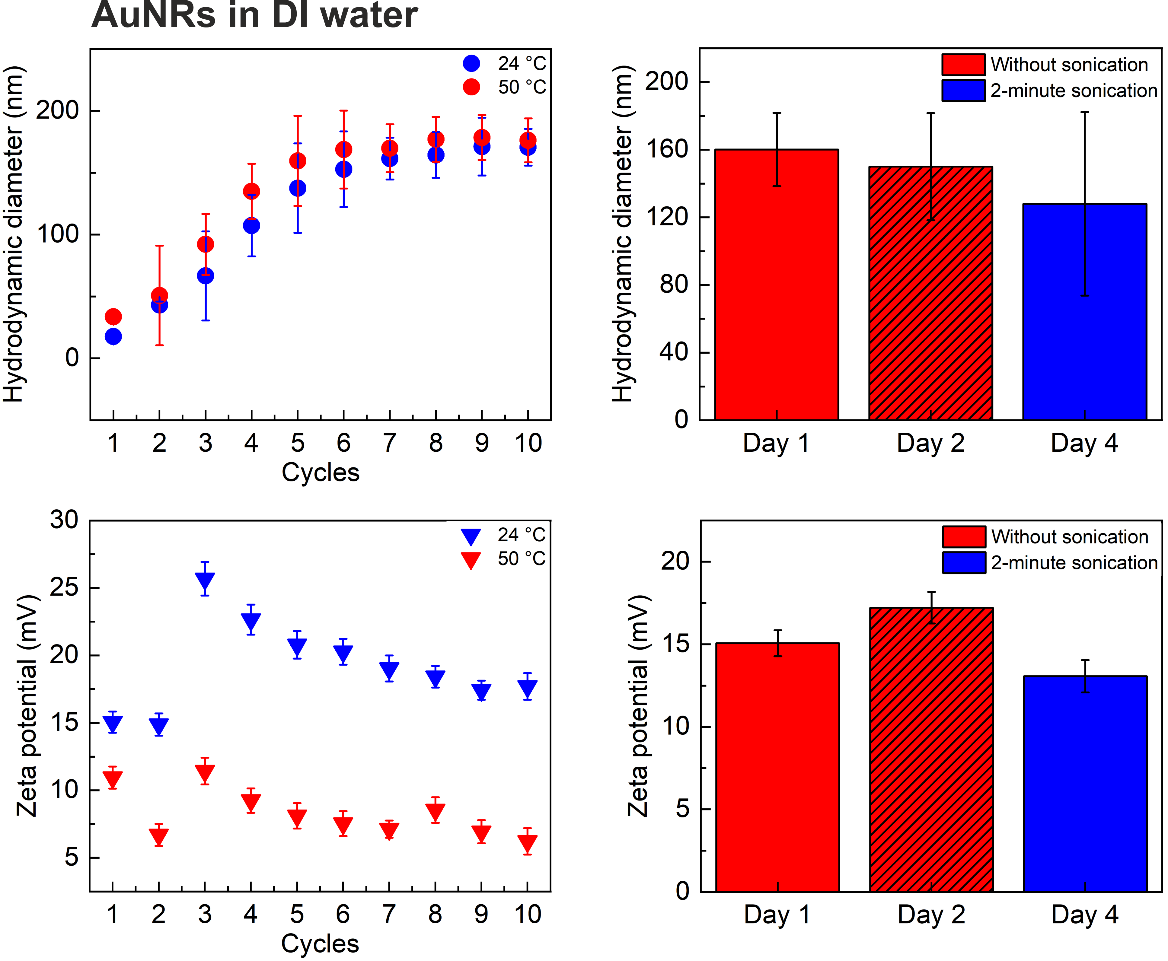
**

**Figure S1.** Long-term stability of gold nanorods was assessed using dynamic light scattering (DLS). Zeta potential and hydrodynamic diameter were measured for 10 heating cycles on Day 1, and subsequently re-evaluated on Day 2 and Day 4 to monitor changes over time.


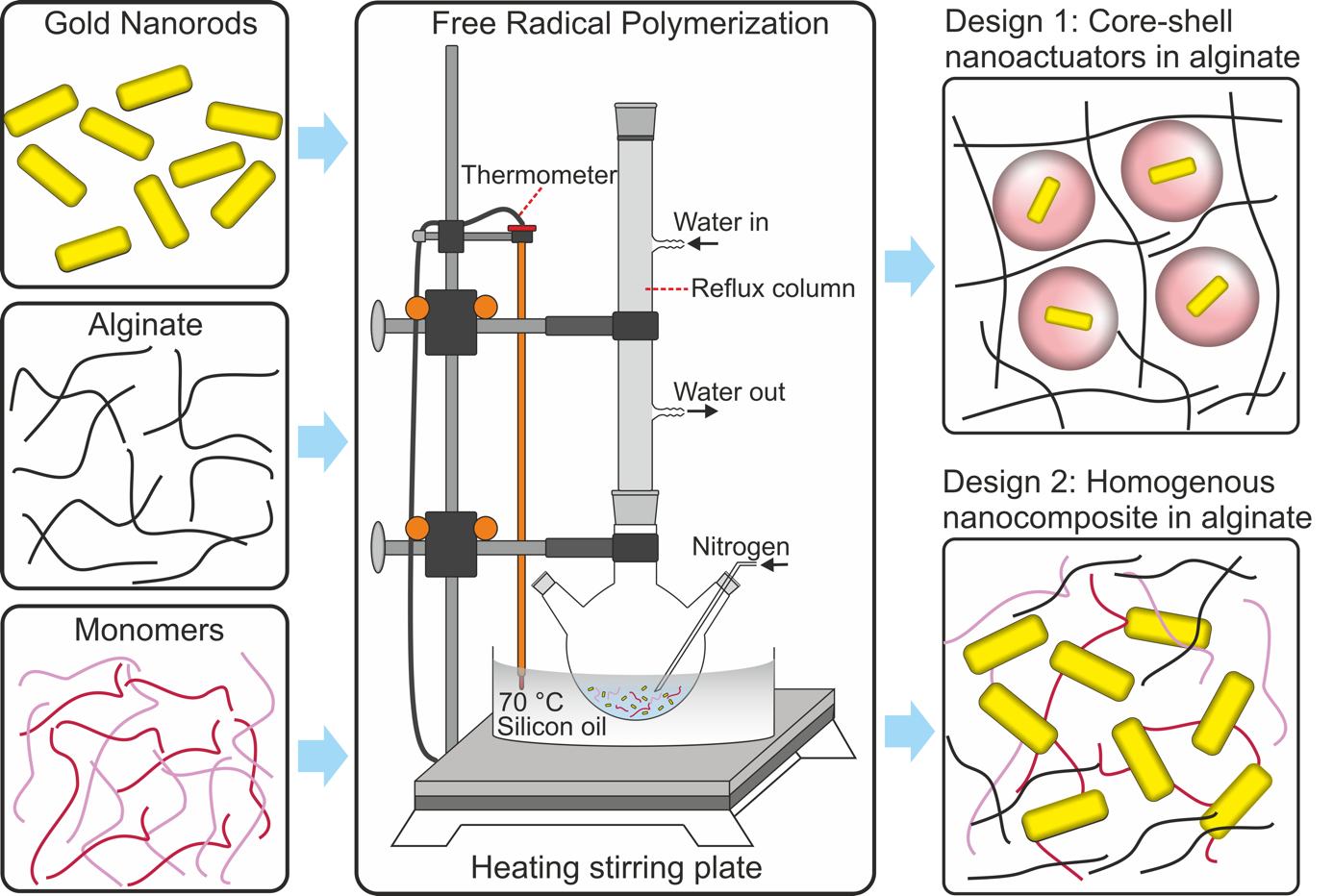


Figure S2. Schematic representation of the synthesis process for thermoresponsive nanoelements via free radical polymerization. Both types of nanoactuators, i.e., core-shell and solid, were prepared with the same approach in separate reactions, either in the presence or absence of gold nanorods, respectively.

**
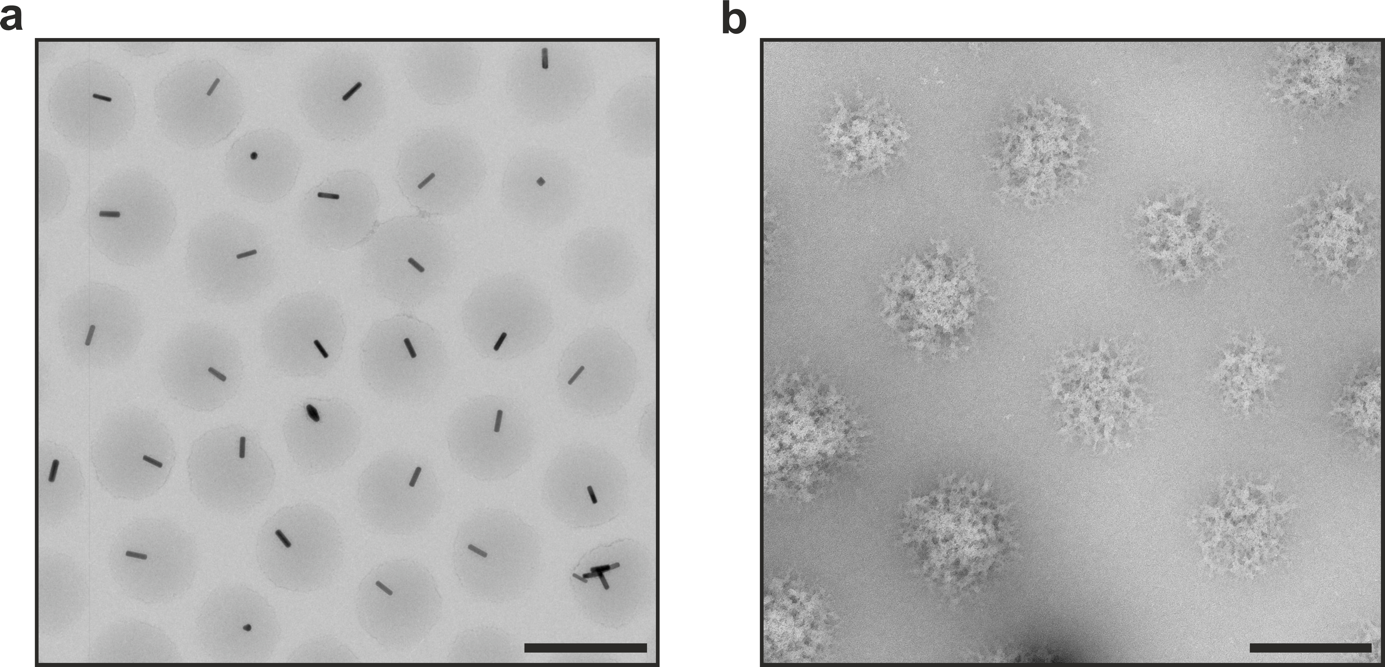
**

Figure S3. TEM images of a) Core-shell nanoactuators (scale bar: 500 nm) and b) solid nanoactuators (scale bar: 500 nm) synthesized using polymer recipe 3.


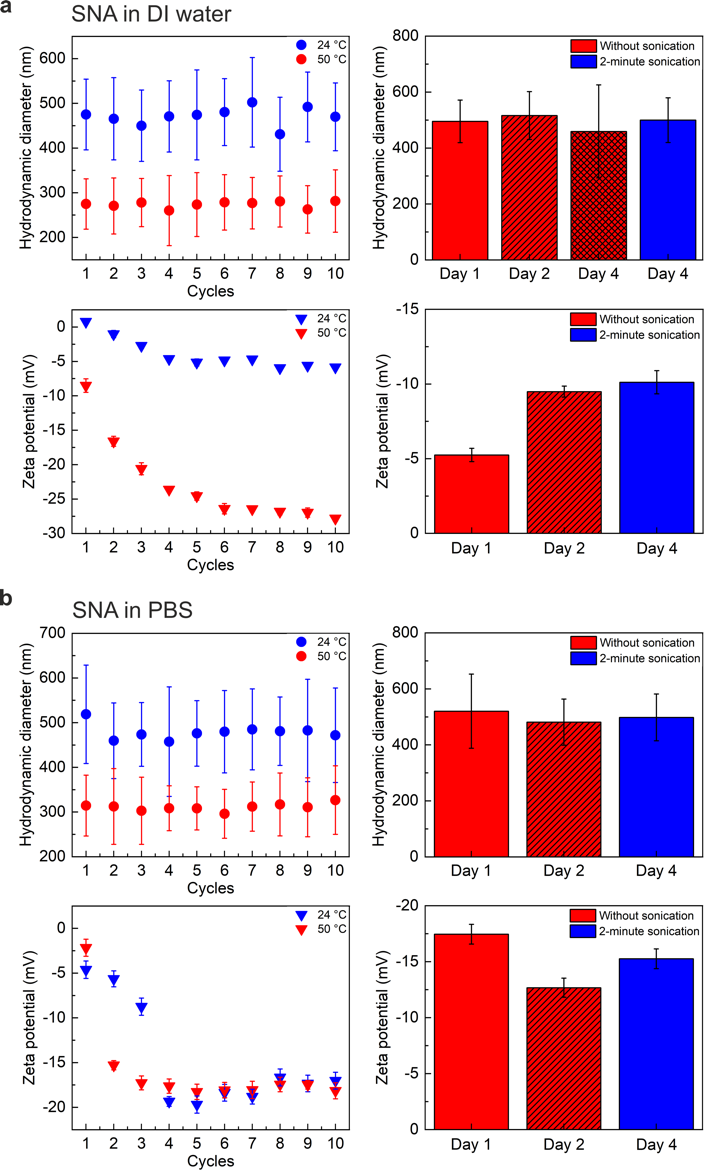


**Figure S4.** The long-term stability of Recipe 3-type thermoresponsive copolymers was evaluated in deionized (DI) water using dynamic light scattering (DLS). Zeta potential and hydrodynamic diameter were measured for 10 heating cycles on Day 1 and re-assessed on Days 2 and 4 to monitor temporal changes.

**
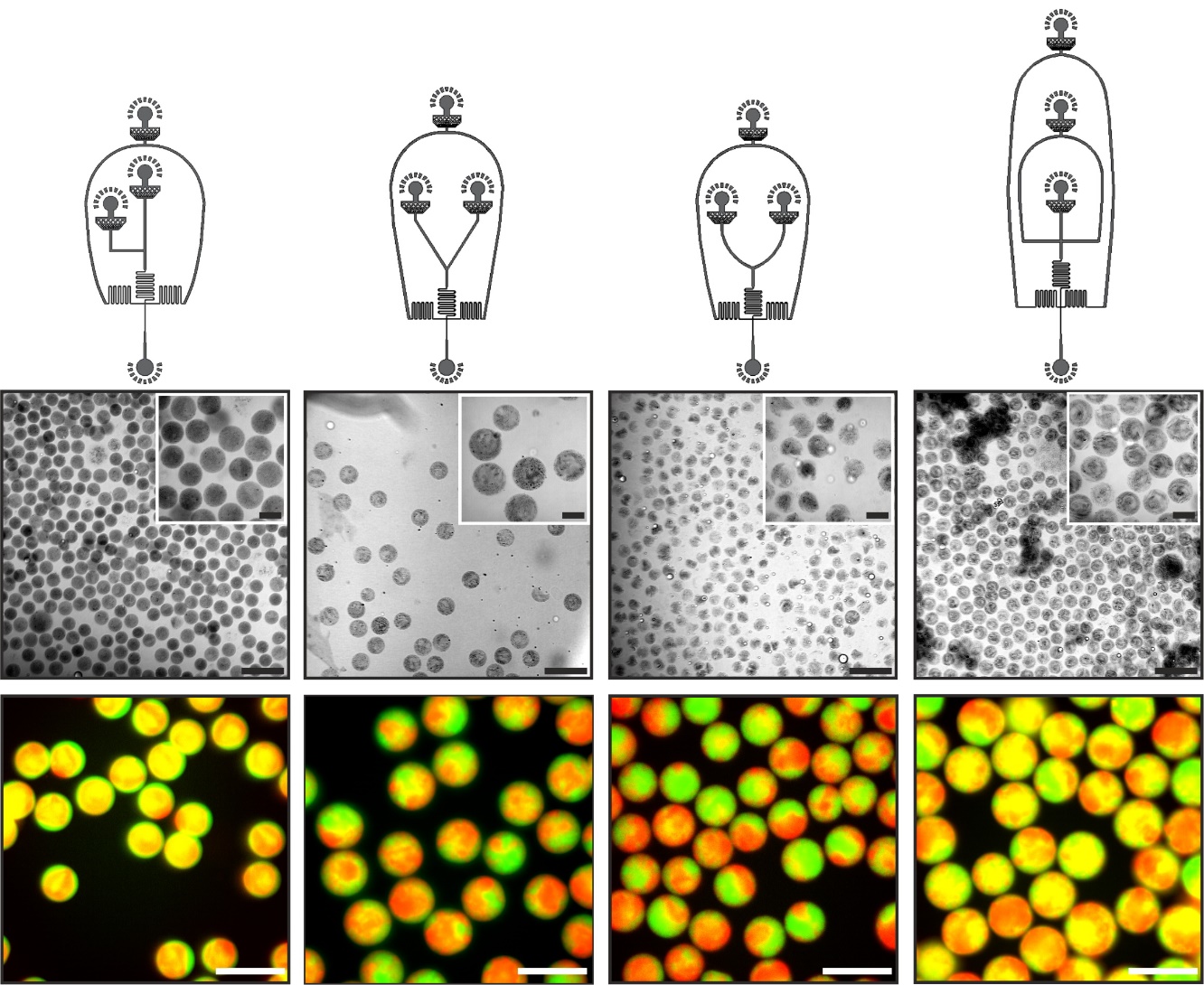
**

Figure S5. Comparison of encapsulation efficiency of varying flow-focusing microfluidic designs, among which the internal designs for aqueous phase mixing are different. Brightfield images of microgels fabricated using the 4 different microfluidic devices demonstrate the influence of device properties on the homogeneity of the resulting microgels. Overview images taken at 10x magnification (scale bar: 200 µm) show variance within the populations and inset images recorded at 40x magnification show heterogeneities within the individual microgels (scale bar: 50 µm). The fluorescence images show the degree of mixing between the two aqueous streams during droplet formation. For this purpose, microgels were constructed with Rhodamine B and Fluorescein-modified alginates (Scare bar: 80 µm). The green and yellow colors correspond to the Rhodamine B and fluorescein, respectively.


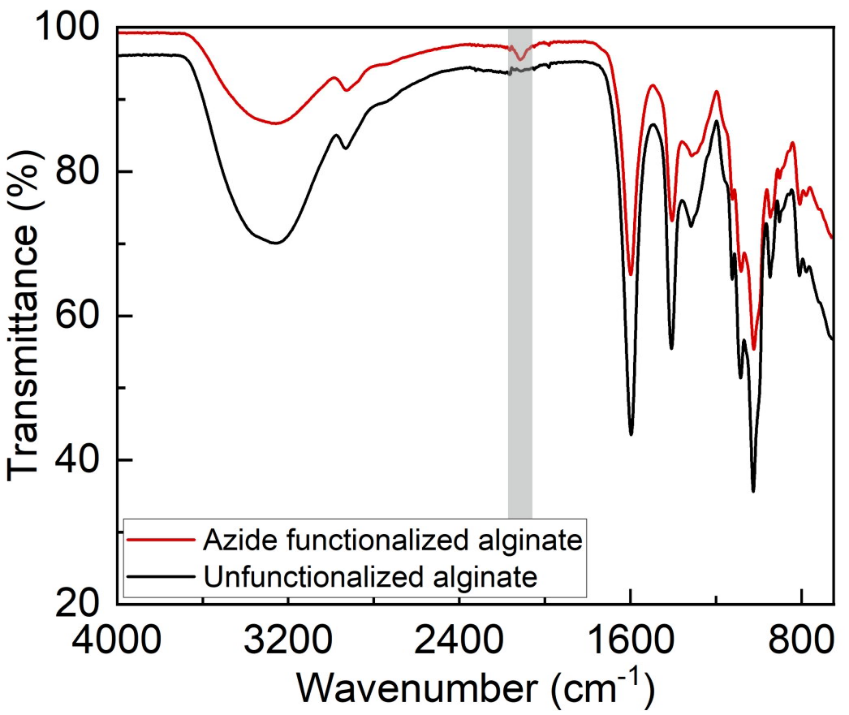


Figure S6. FTIR spectrum of azide-functionalized alginate (red) compared to pure, unfunctionalized alginate (black). The peak at 2110 cm^-1^ indicates the azide group in the modified alginate, which is absent in the unfunctionalized alginate.


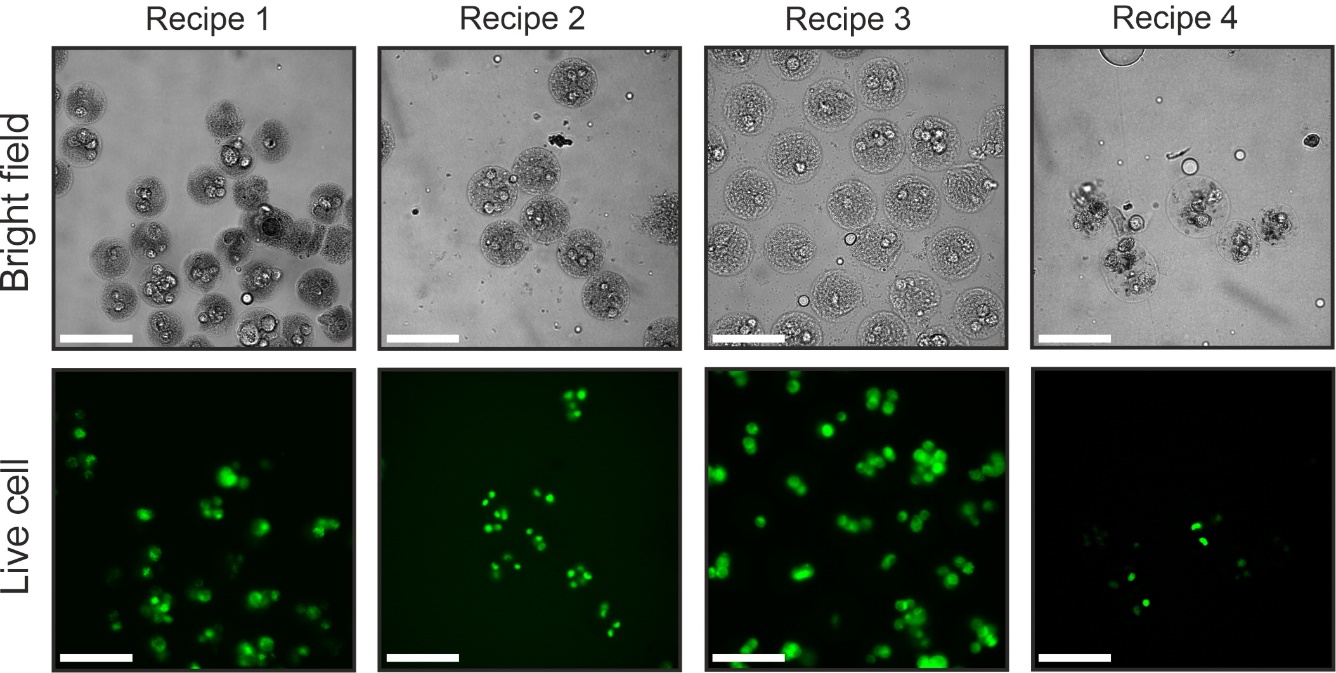


Figure S7. Brightfield microscopy images show microgels fabricated using the T-junction microfluidic device with core-shell nanoactuators containing four different types of random co-polymers ranging from recipe 1 to recipe 4 (from left to right, scale bar: 100 µm). The underlying fluorescence images show encapsulated live cells strained using calcein AM (scale bar: 100 µm).


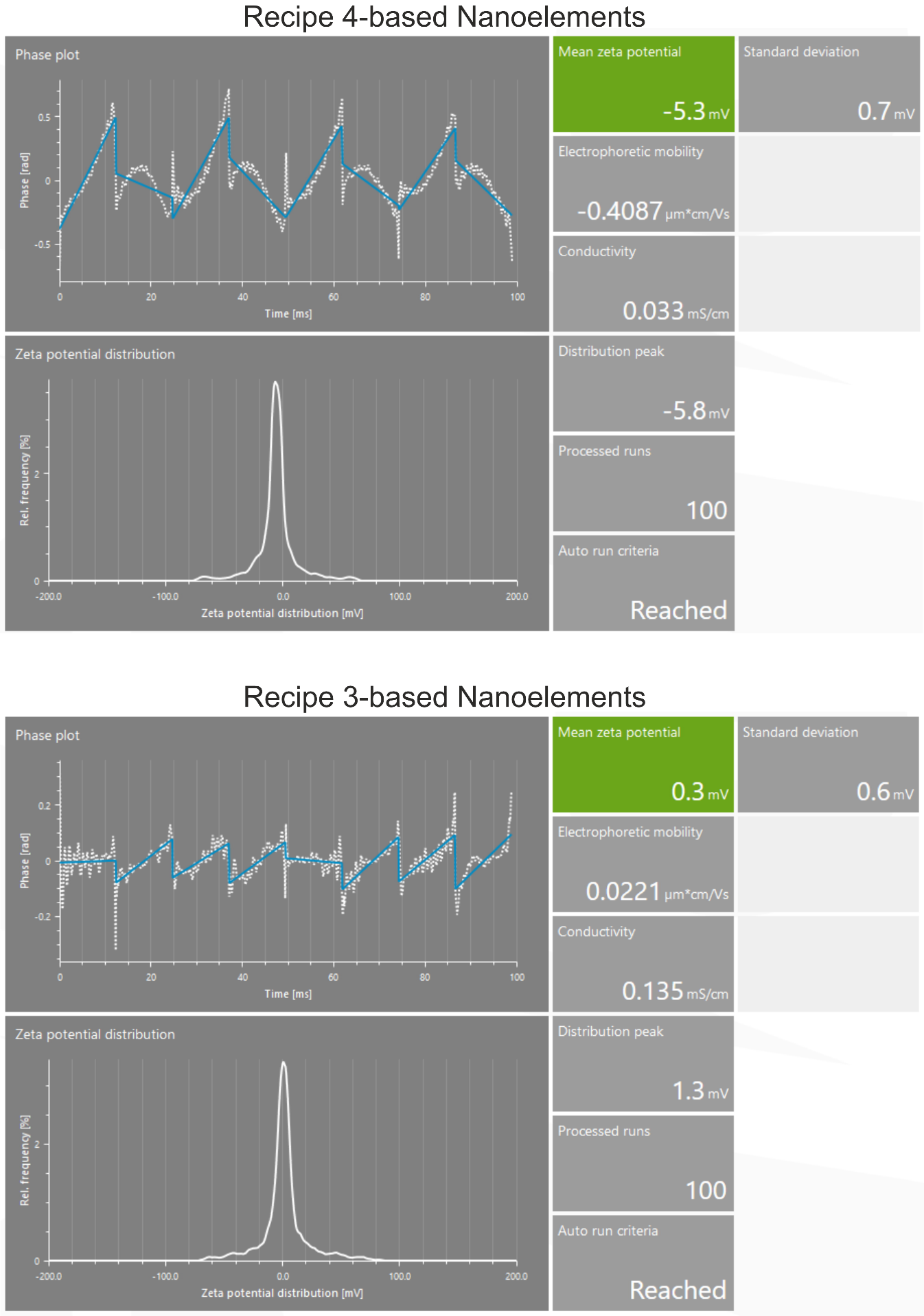


Figure S8. Comparison of zeta potentials measured for thermoresponsive nanoelements, specifically solid core-shell nanoactuators, using recipe 3 and recipe 4 at room temperature.


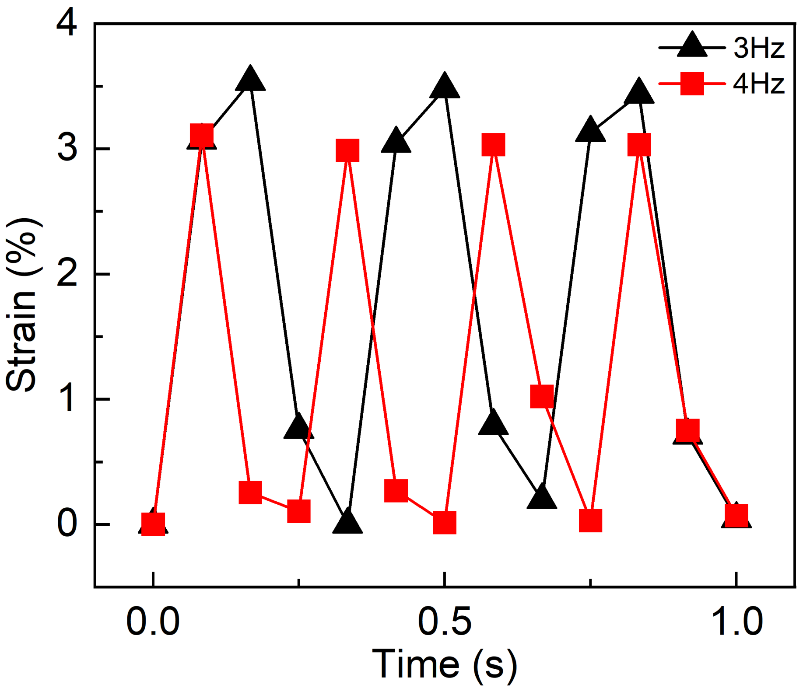


Figure S9. Radial strain profile of photothermally powered D2-type microgels was measured over time at 3 and 4 Hz actuation frequency.


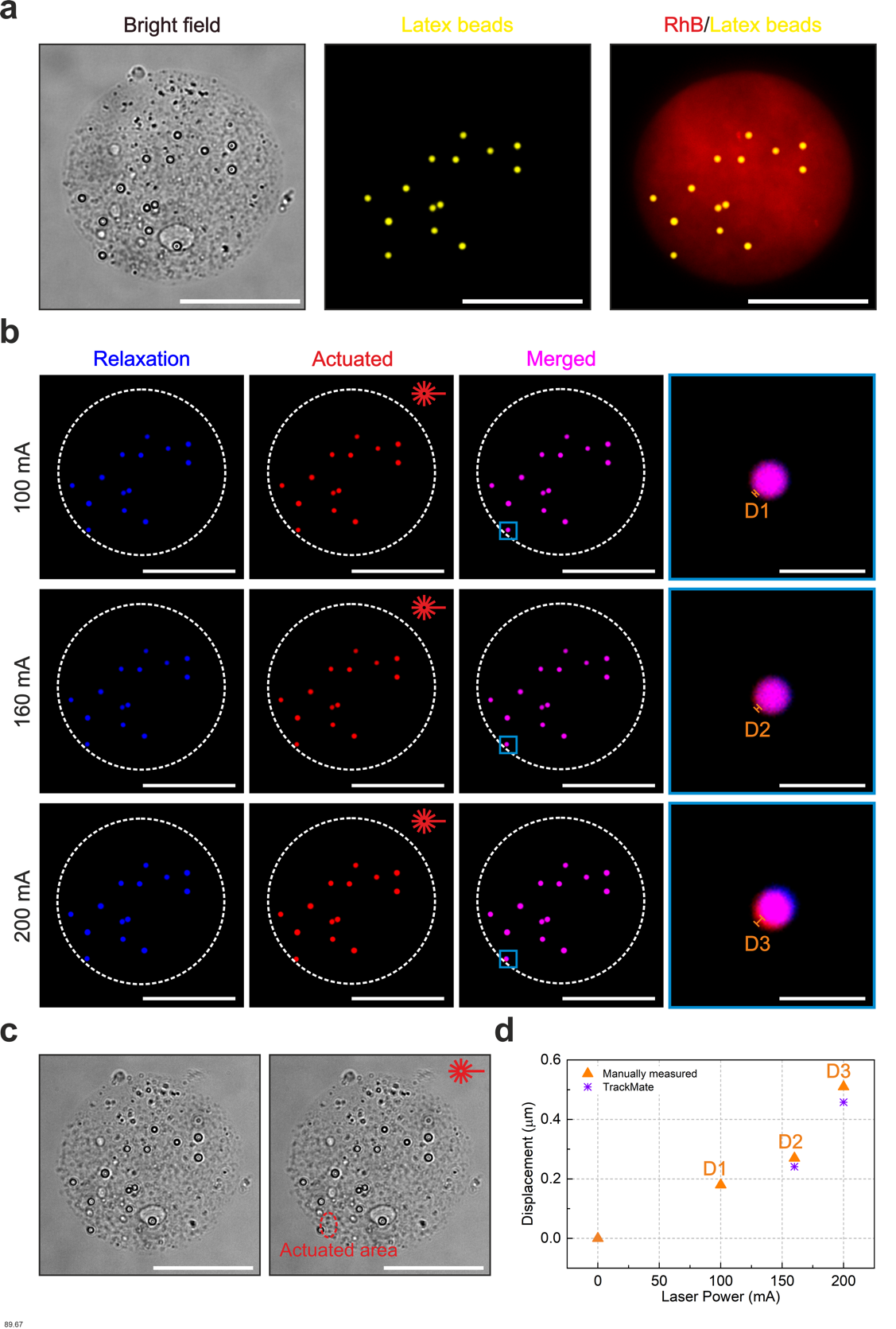


Figure S10. Spatial resolution of photothermally powered 3D microgels. a) Brightfield and fluorescence images of microgels containing 0.03 % fluorescently labeled polystyrene nanoparticles (Latex beads, 2.0 µm, Sigma Aldrich). The yellow channel indicates the nanoparticles, and the red channel shows the Rhodamine B-labeled alginate network (scale bar: 40 µm). b) Fluorescence images of fluorescent beads in the microgels under relaxed states and actuated states at increasing laser power, 100 mA (3.93 µW µm^-2^), 160 mA (7.95 µW µm^-2^), and 200 mA. The laser beam was positioned to partially expose the lower left corner of the microgel, resulting in the movement of beads (scale bar: 40 µm for the first three columns of images and 5 µm for the last column of zoomed images). c) Brightfield images show the microgel morphology at relaxed (left) and actuated states (right, 100 mA), where the red dashed line represents the area actuated by the laser (scale bar: 40 µm). (d) The graph shows the displacement quantification of a representative latex bead aided by the TrackMate plugin in Fiji software.


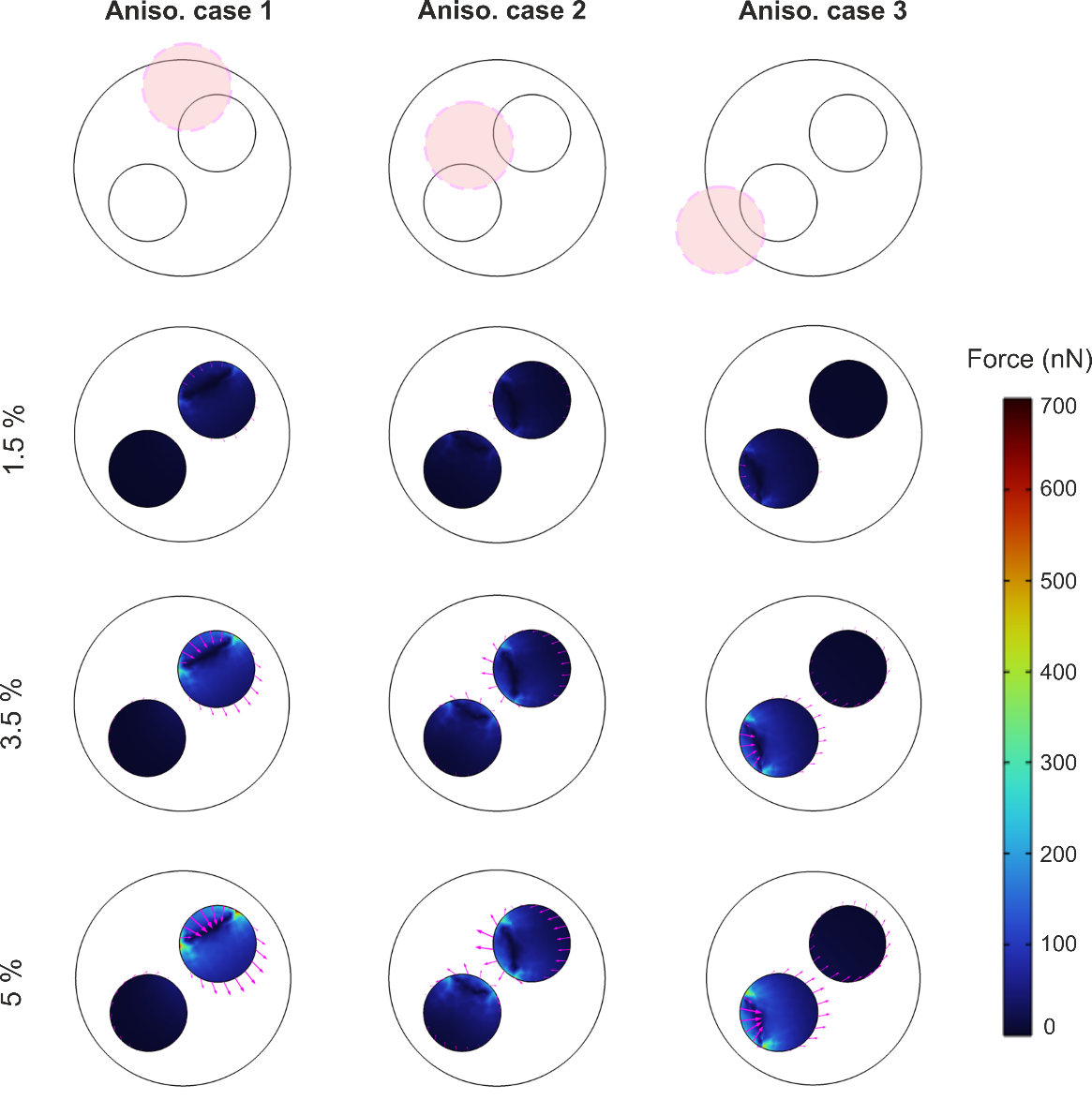


**Figure S11**. Finite element method (FEM) analyses reveal distinct force distribution patterns under three representative anisotropic actuation conditions, highlighting the enhanced force-controlled capabilities of our multicellular 3D microgels.


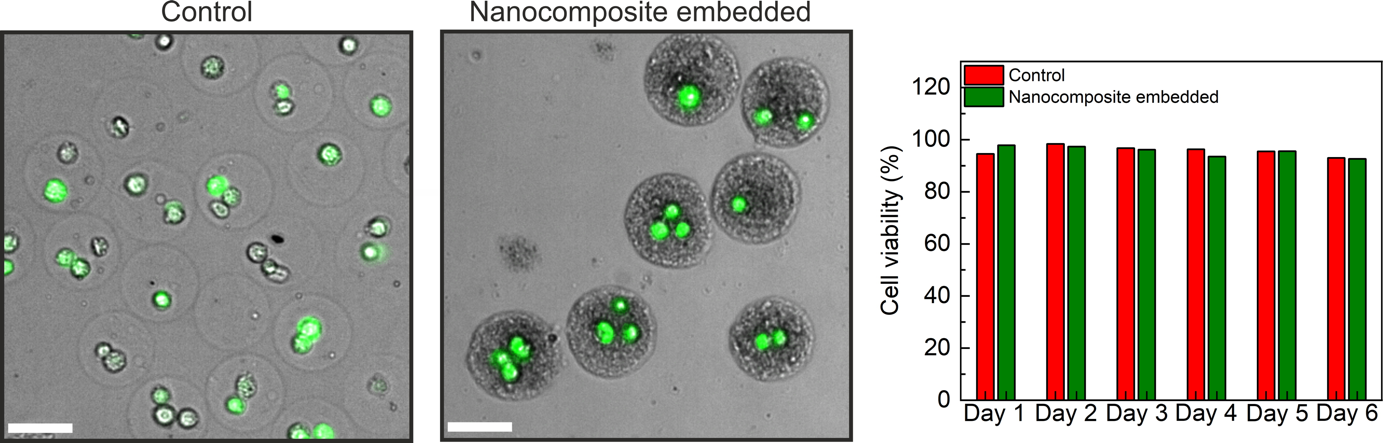


**Figure S12.** Representative multichannel images of encapsulated cells in only RGD-modified alginate microgels and D2-type microgels with nanocomposite structure (scale bars: 40 µm), indicating viable cells (green labeled). Corresponding analysis of cell viability in both types of microgels over 6 days in culture. For both conditions, cells were encapsulated on Day 0 and cultured in a cell culture plate at 37°C without any agitation. Viability assay was conducted using live-dead staining via Calcein AM – Ethidium homodimer 1 markers.

**
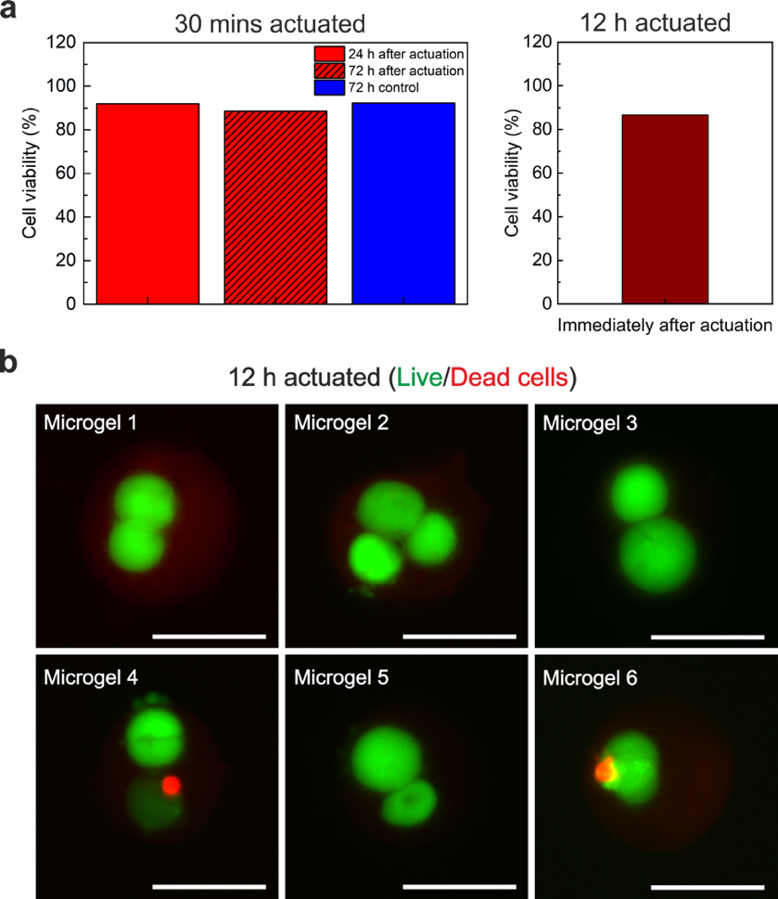
**

**Figure S13.** Long-term viability under continuous exposure to photothermal actuation is presented for 30 minutes and 2 hours of actuation. Actuation was performed isotropically using a laser power of 4.45 µW µm^-2^ with a 1 Hz frequency (50% duty cycle). a) Viability plots are shown for 30 minutes of actuation and 12 hours separately using live-dead staining. The measurement time points for the 30-minute actuation condition were 24 hours (n=62) and 72 hours (n=52) after stimulation, compared to 72-hour control cells (n=39). The 12-hour-long actuated cells were analyzed for viability immediately after actuation (n=14). b) Images of live-dead stained cells residing in the photothermally powered microgels, at the end of the 12-hour actuation period (scale bar: 40 µm).


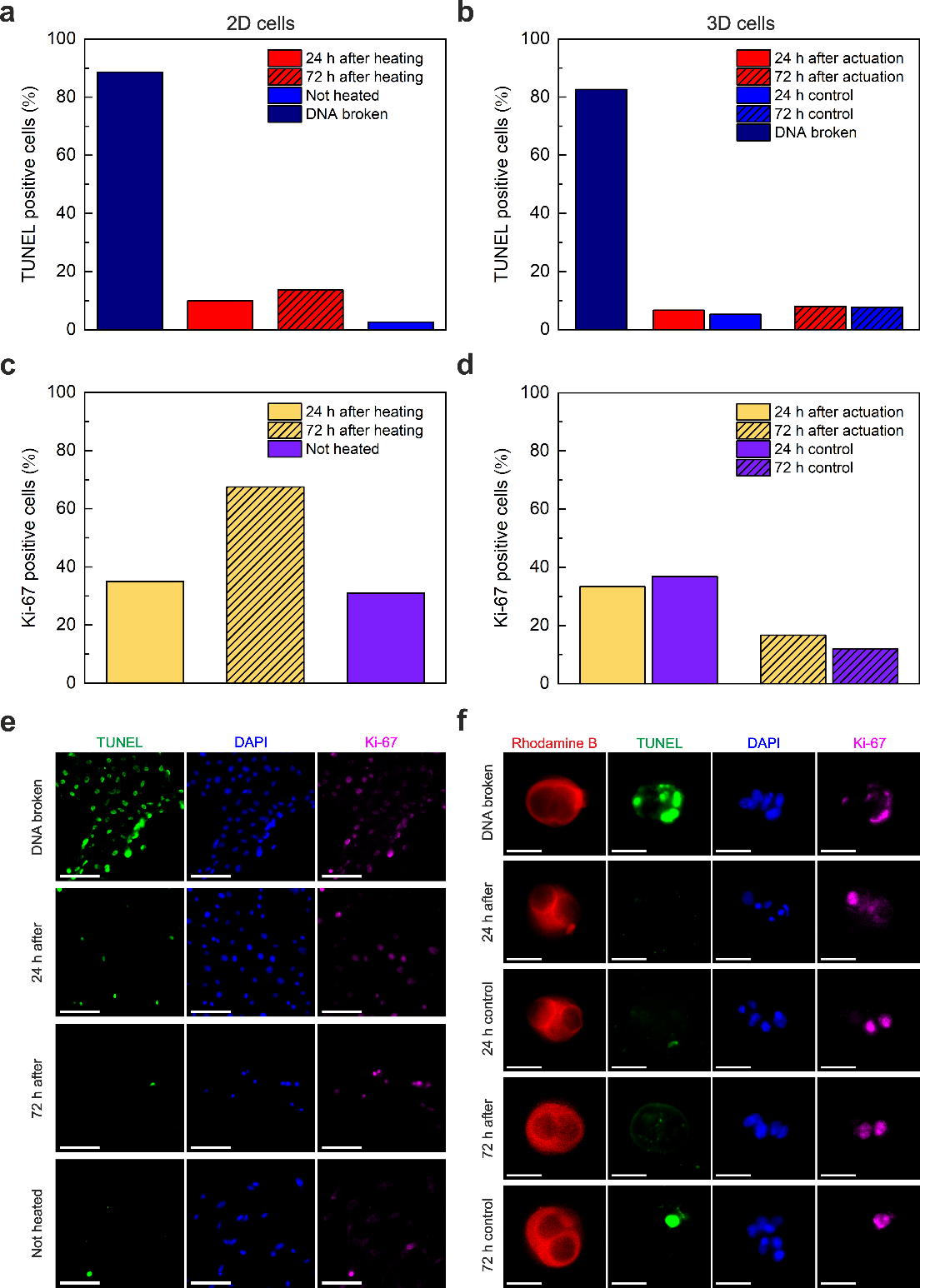


Figure S14. The influence of heat loads on stem cells cultured over 2D substrates and within photothermally powered 3D microgels. a) The percentage of apoptotic cells determined using the TUNEL assay is shown for 2D culture conditions corresponding to DNA broken positive control (n=79), 24 hours after thermal stimulation (n=120), 72 hours after thermal stimulation (n=80), and not heated, negative control (n=113) samples. Thermal stimulation was performed by subjecting the 2D cultured cells to 42°C for 30 minutes using a stage top heater. b) Apoptotic cell percentages in encapsulated cells subjected to DNA break (n=23), 24 hours after photothermal actuation (n=15) with the corresponding unactuated control cells (n=25), and 72 hours after actuation (n=25) compared to their control (n=26) are shown. Photothermal stimulation was performed isotropically at 4.45 µW µm^-2^ using 1 Hz frequency for 30 minutes. Both 2D substrate-bound and 3D encapsulated cells were evaluated using TUNEL immunostaining for apoptosis. Proliferation capacity of heat-treated cells c) cultured over 2D substrates (n=120 for 24 hours after actuation, n=80 for 72 hours after actuation, and n=113 for not actuated control cells) and d) encapsulated in photothermally powered microgels (n=15 for 24 hours after actuation and n=19 for 24-hour control; n=25 for 72 hours after actuation and n=26 for 72-hour control). The percentage of Ki-67 positive cells indicates proliferation, and the actuation parameters were kept the same as in the apoptosis assay. Representative fluorescence image series of e) thermally stimulated cells under 2D culture conditions (scale bar: 100 µm) and f) 3D encapsulated cells in photothermally powered microgels (scale bar: 30 µm).


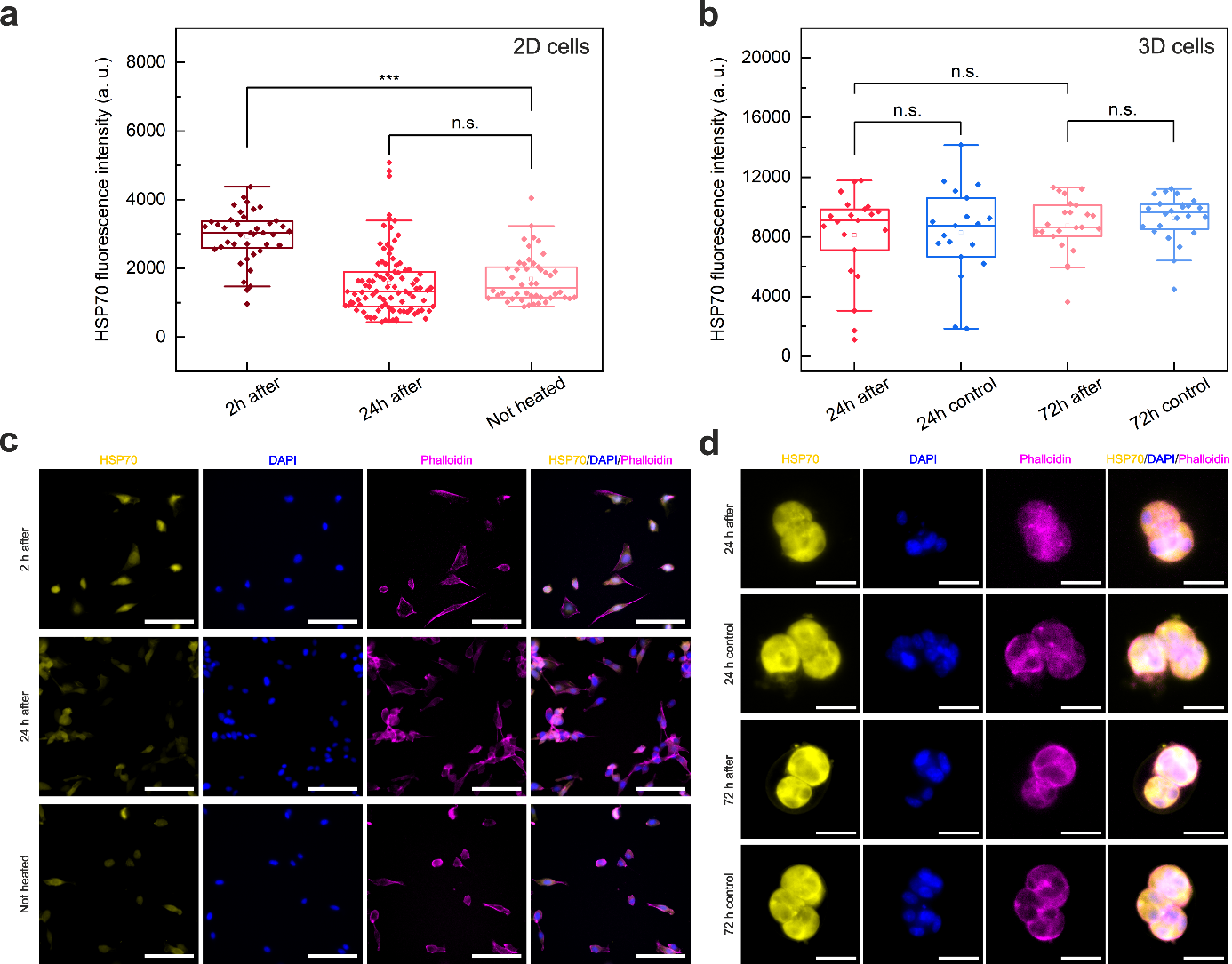


Figure S15. Cell response to heat loads, measured using the heat stress marker HSP70. a) Fluorescence HSP70 signal (arbitrary units) measured in stem cells cultured over 2D substrates corresponding to 2 hours (n=40) and 24 hours (n=93) after being subjected to continuous heating at 42°C for 30 minutes using a stage top heating plate, versus not heated, control cells (n=46). b) Heat shock protein activity in encapsulated cells residing in photothermally powered 3D microgels is shown for 24 hours (n=21) and 72 hours (n=23) after actuation with corresponding unactuated control microgels for 24 hours (n=19) and 72 hours (n=24). Photothermally powered microgels were actuated at 4.45 µW µm^-2^ with 1 Hz (50% duty cycle) for 30 minutes. Representative fluorescence images for all conditions are provided in c) 2D cultured MSCs (scale bar: 100 µm) and d) encapsulated cells in 3D microgels (scale bar: 30 µm). Yellow indicates HSP70 intensity, blue shows nucleus DAPI, and magenta indicates phalloidin for F-actin. Error bars represent the range of the data, excluding outliers, n.s. indicates not significant difference, and *p<0.001 indicates significant difference (one-way ANOVA).


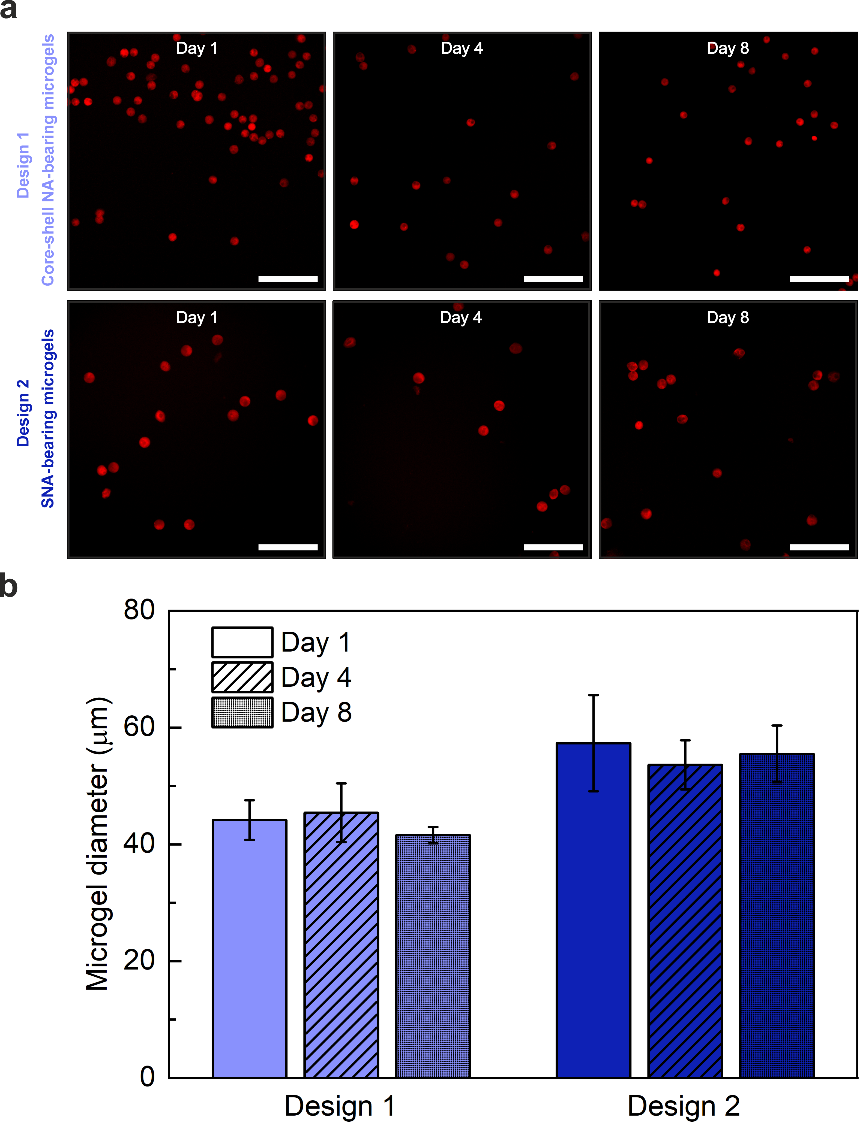


**Figure S16.** Mechanical stability of cell-carrying microgels. a) Representative fluorescence images of multicellular microgels for Designs 1 (microgels with core-shell nanoactuators) and 2 (solid nanoactuator and gold nanorod containing microgels with homogeneous nanocomposite structure) over 8 days in culture. The red signal indicates the Rhodamine B-labeled alginate network making up the microgels. b) Changes in microgel diameter for Design 1 and Design 2 throughout culture on Days 1, 4, and 8 (scale bar: 300 µm). A two-way ANOVA was performed to assess the effects of microgel design, culture time, and their interaction on microgel diameter. Post hoc analysis using Tukey’s multiple comparisons test revealed no statistically significant differences between time points or designs (p > 0.05).


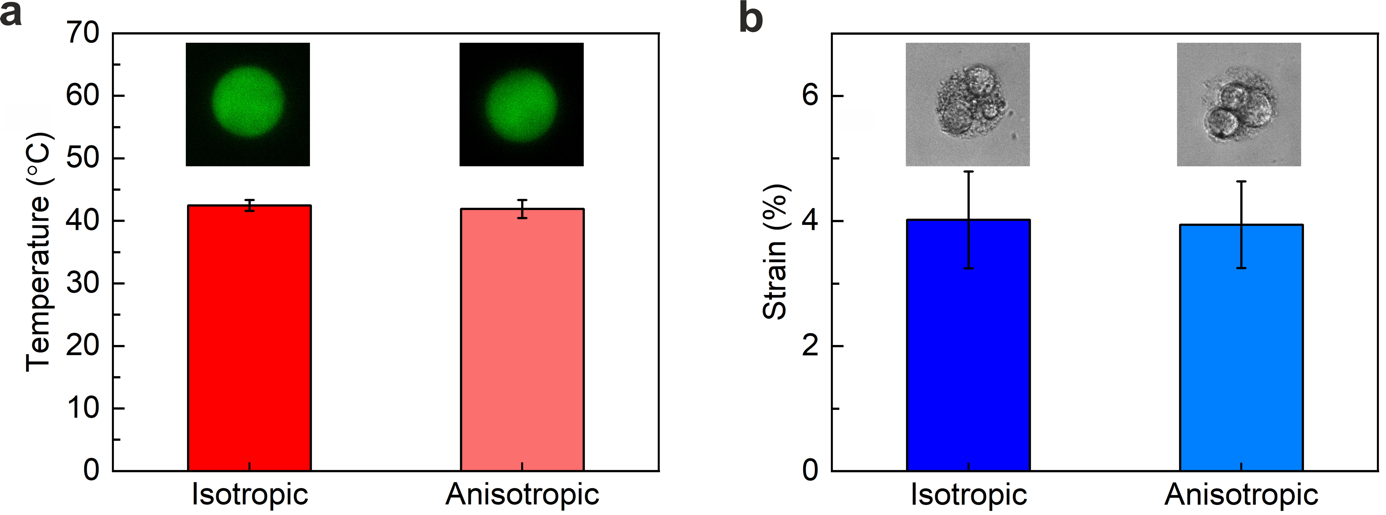


**Figure S17.** Plots show average of maximum temperature and strain in D2-type photothermally powered 3D microgels with encapsulated cells under isotropic and anisotropic actuation. Error bars indicate standard deviation (n=10).


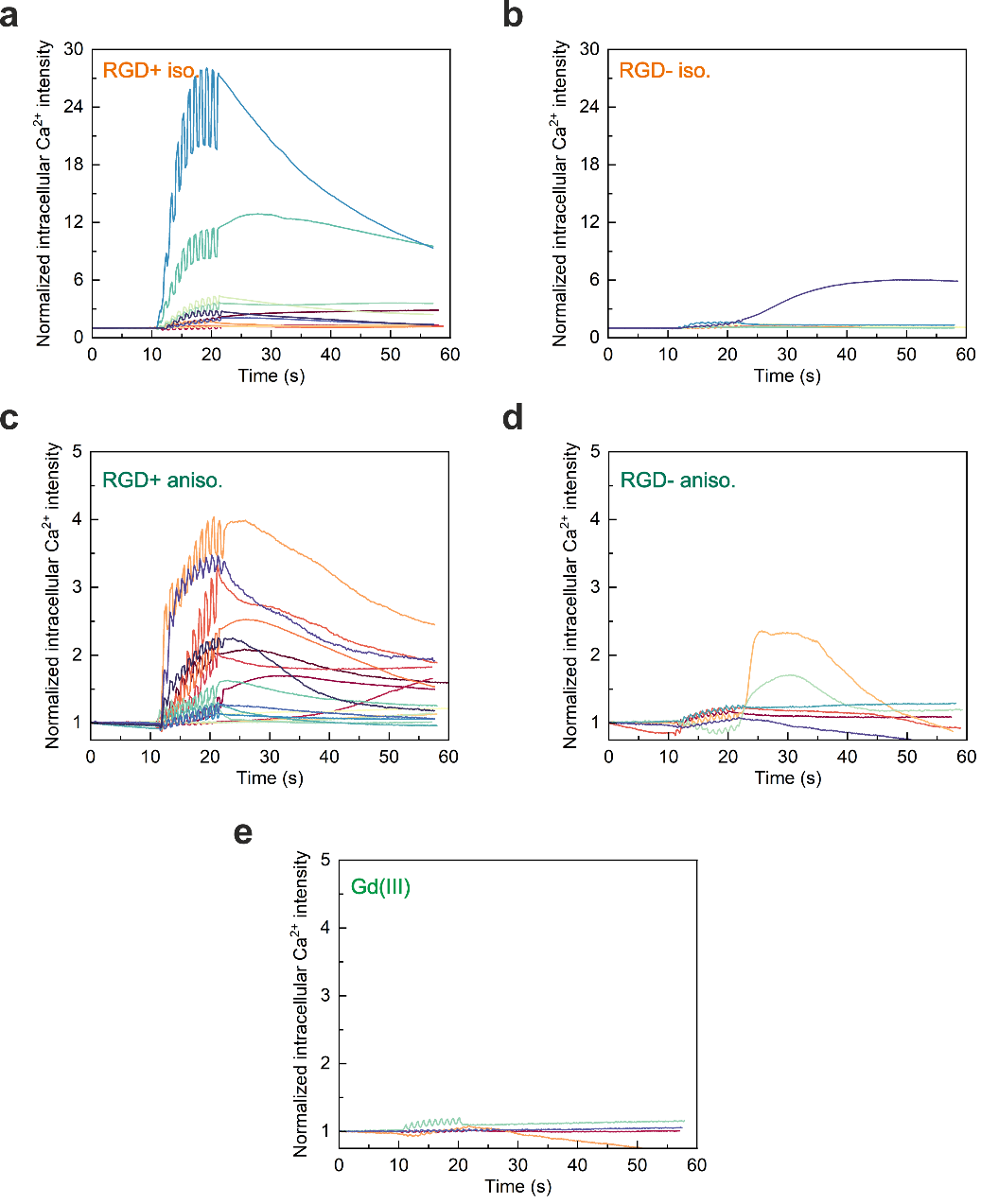


**Figure S18.** The plots a) - e) show the normalized transient intracellular calcium of encapsulated cells within photothermally powered 3D microgels, which corresponds to those presented in the main manuscript.

**
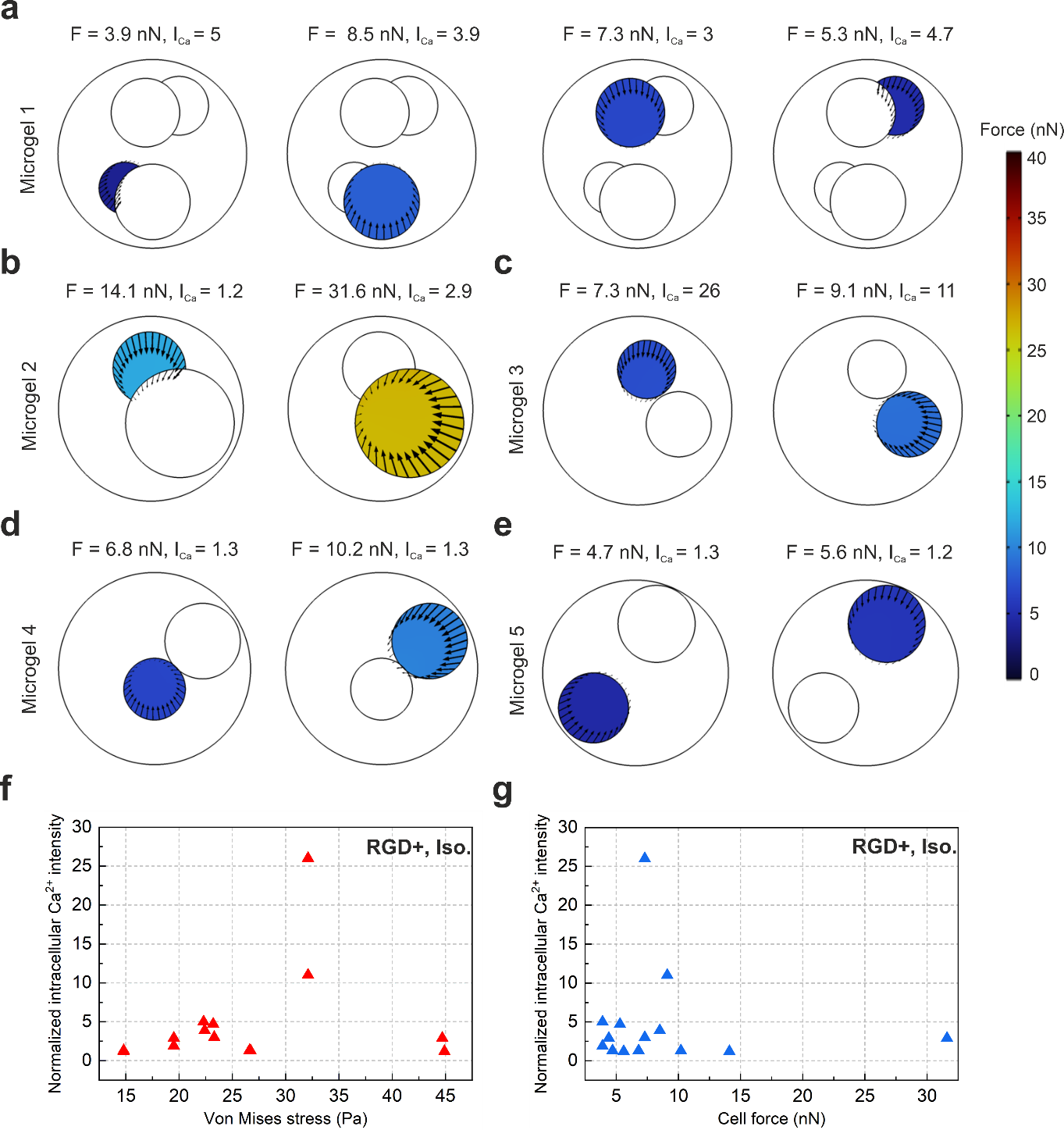
**

**Figure S19.** a) - e) A series of 2D force maps generated by FEM analysis quantifies the force experienced by individual cells encapsulated in isotropically actuated RGD-presenting microgels used in intracellular calcium signaling studies presented in the main manuscript. f) Von Mises stress and g) force acting on the encapsulated cells with the corresponding experimentally measured maximum intracellular calcium intensity are plotted in separate graphs.

**
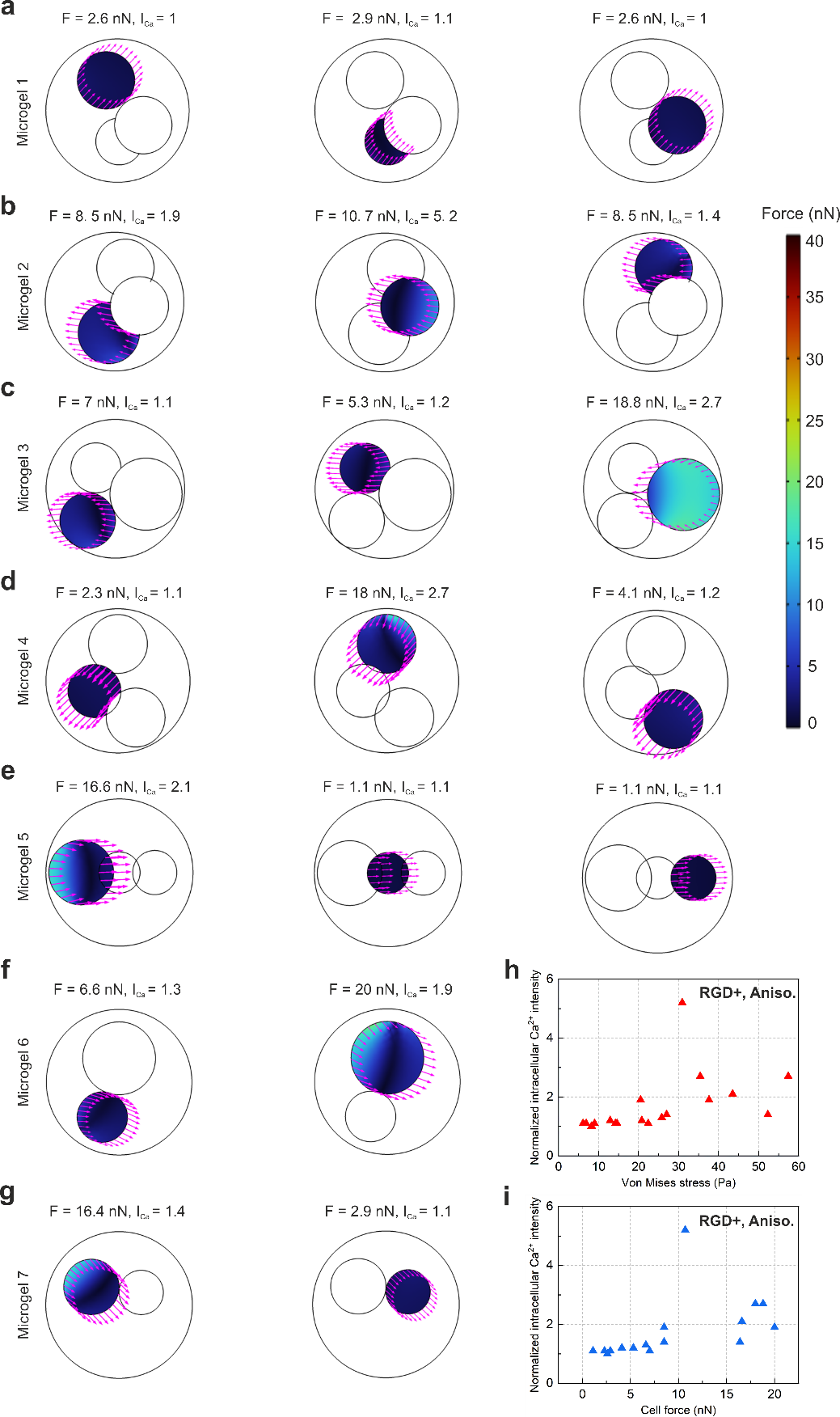
**

**Figure S20.** a)-g) A series of 2D force maps generated by FEM analysis quantifies the force experienced by individual cells encapsulated in anisotropically actuated RGD-presenting microgels used in intracellular calcium signaling experiments presented in the main manuscript. h) Von Mises stress and i) force acting on the encapsulated cells with the corresponding experimentally measured maximum intracellular calcium intensities are plotted in separate graphs.


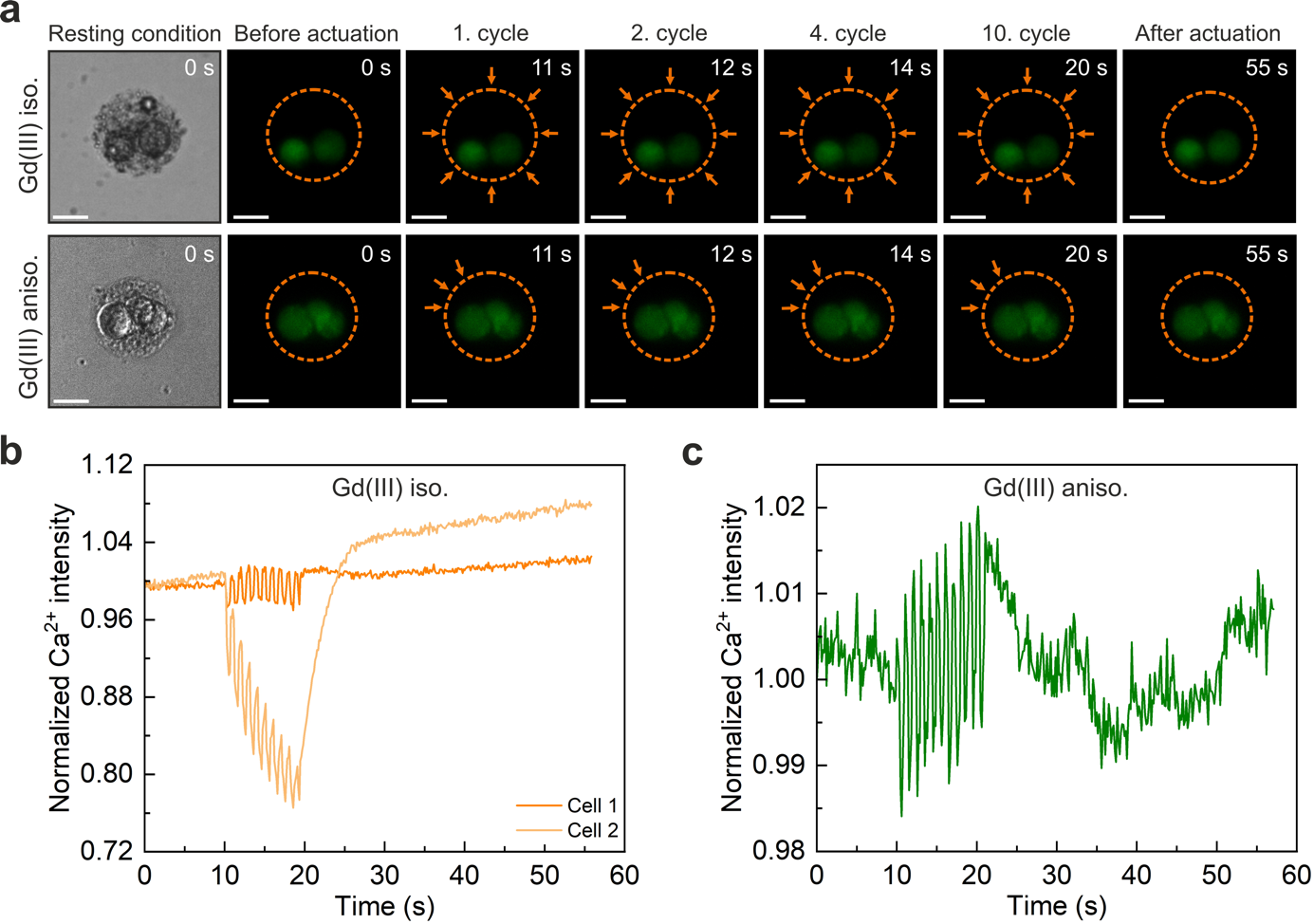


**Figure S21.** Control experiments on encapsulated cells using Gd3+. a) Fluorescent image series over time shows a lack of changes in intracellular calcium signaling under isotropic and anisotropic forces in the presence of Gd^3+^. Corresponding plots showing changes in normalized intracellular calcium intensity for b) isotropically actuated cells and c) anisotropically stimulated encapsulated stem cells (scale bar: 20 µm).


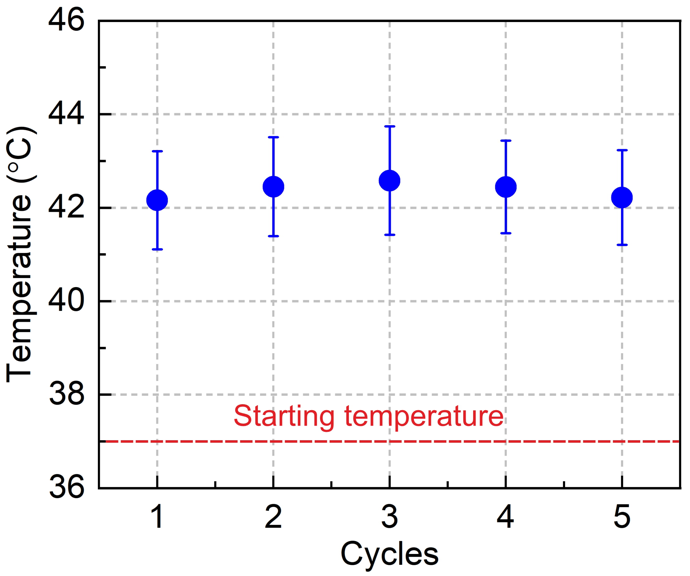


**Figure S22.** The average of maximum microgel temperature is plotted for 5 cycles of actuation, quantified by Rhodamine B-based microthermometry (n=10 for cycles 1-3 and n=5 for cycles 4-5). The photothermally powered microgels (RGD-presenting) contained multiple cells, and encapsulation was performed using a 3 mg mL^-1^ gold nanorod concentration. The concentrations of all other components were kept constant, and microfluidic encapsulation was performed as described in the manuscript. The microgels were actuated at 4.45 µW µm^-2^.


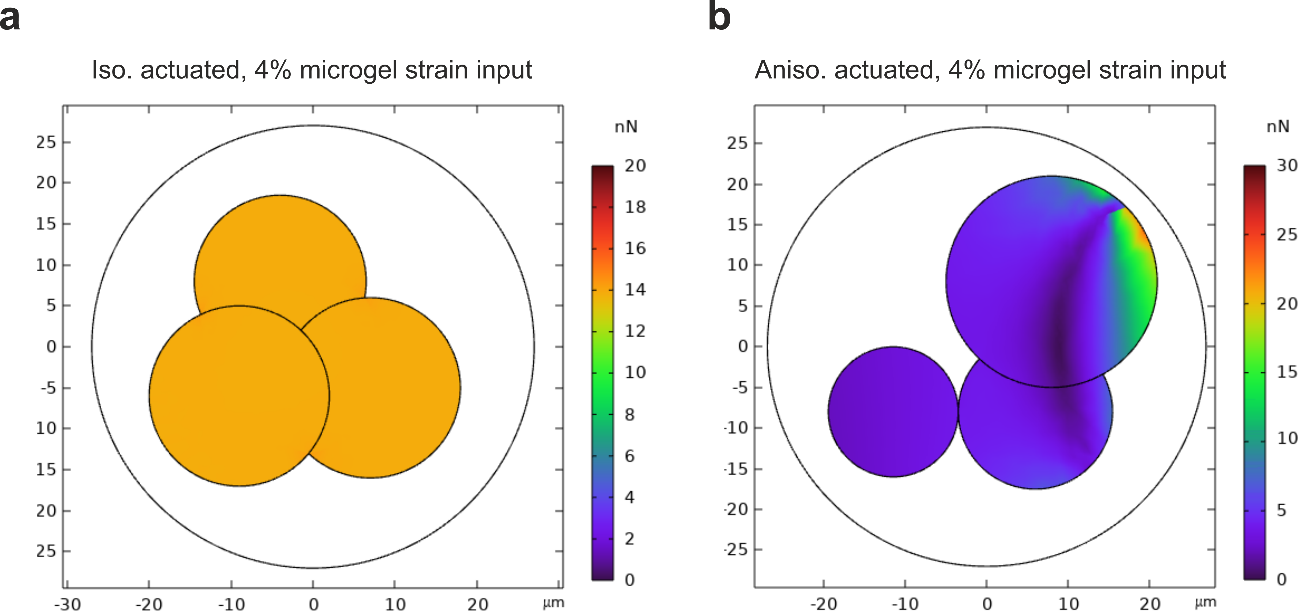


**Figure S23.** Force distribution plots of multicellular microgels actuated at 4.45 µW µm^-2^ laser power, resulting in 4% radial deformation, for a) isotropically and b) anisotropically actuated cases. The placement of the cells within the microgels corresponds to those shown F-actin experiments presented in the main manuscript.


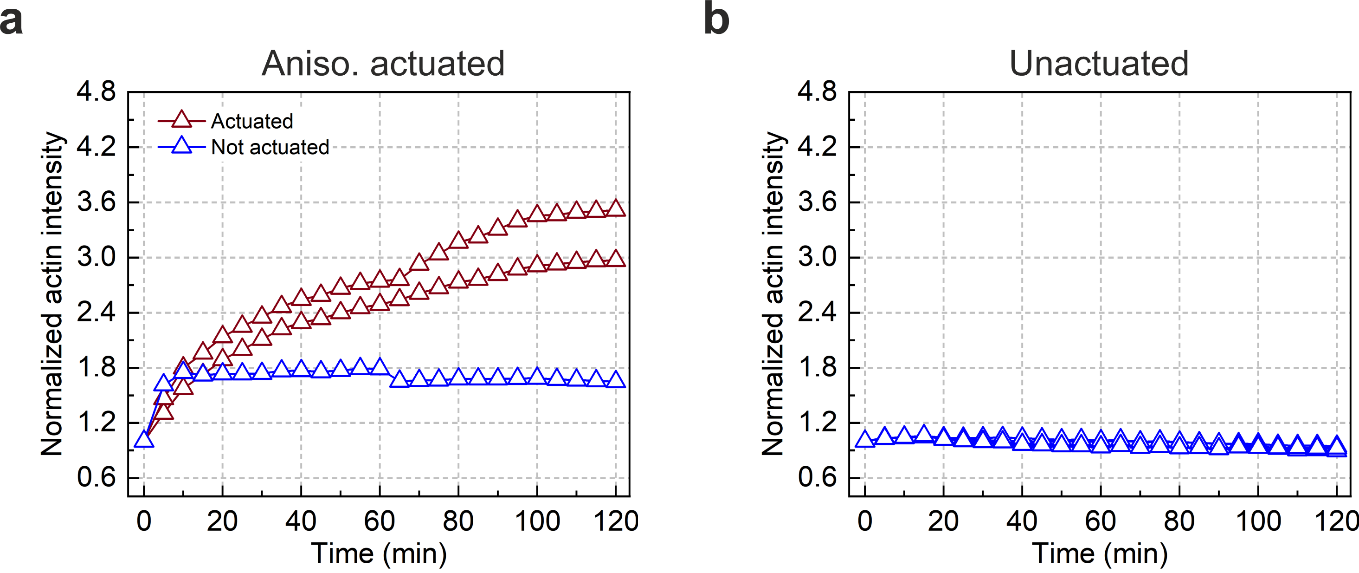


**Figure S24.** The graphs show the change in F-actin fluorescence intensity of the encapsulated cells within either a) anisotropically actuated microgels or b) unactuated control microgels, corresponding to the image series of cells presented in Figure 7c.


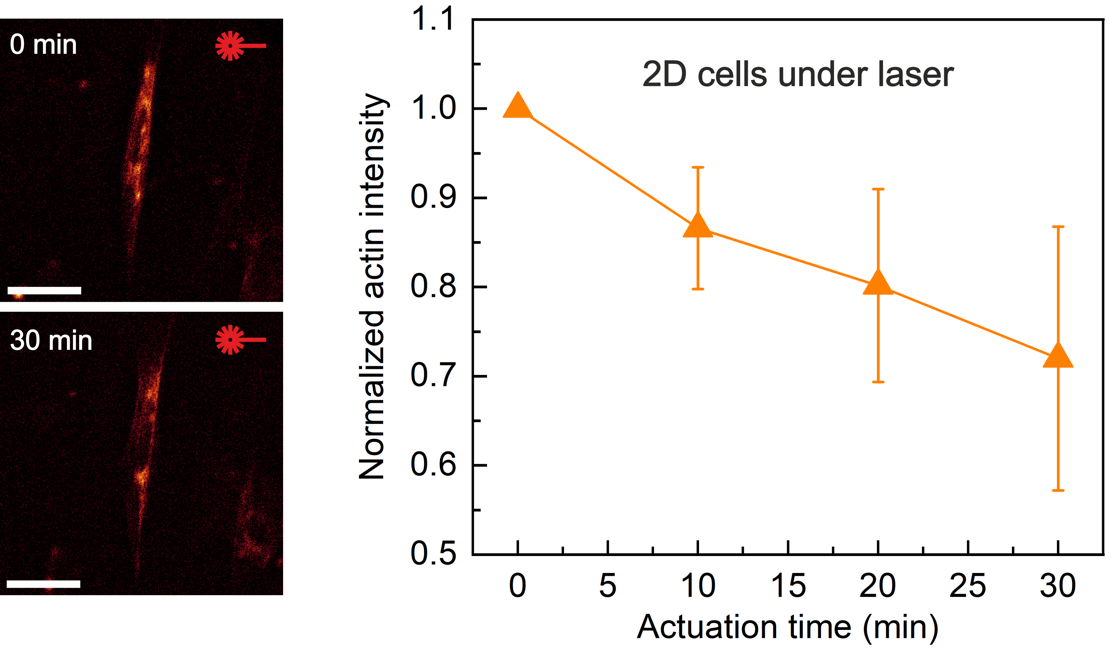


Figure S25. The change in F-actin intensity of stem cells cultured over conventional 2D substrates is shown under NIR laser without the photothermally powered microgels (n=5). Cells were subjected to the NIR laser (100 mA, 3.1 µW µm^-2^) at 1 Hz frequency (50% duty cycle) for 30 minutes. The decrease in F-actin intensity is likely due to photobleaching. Error bars indicate standard deviation (scale bar: 30 µm).


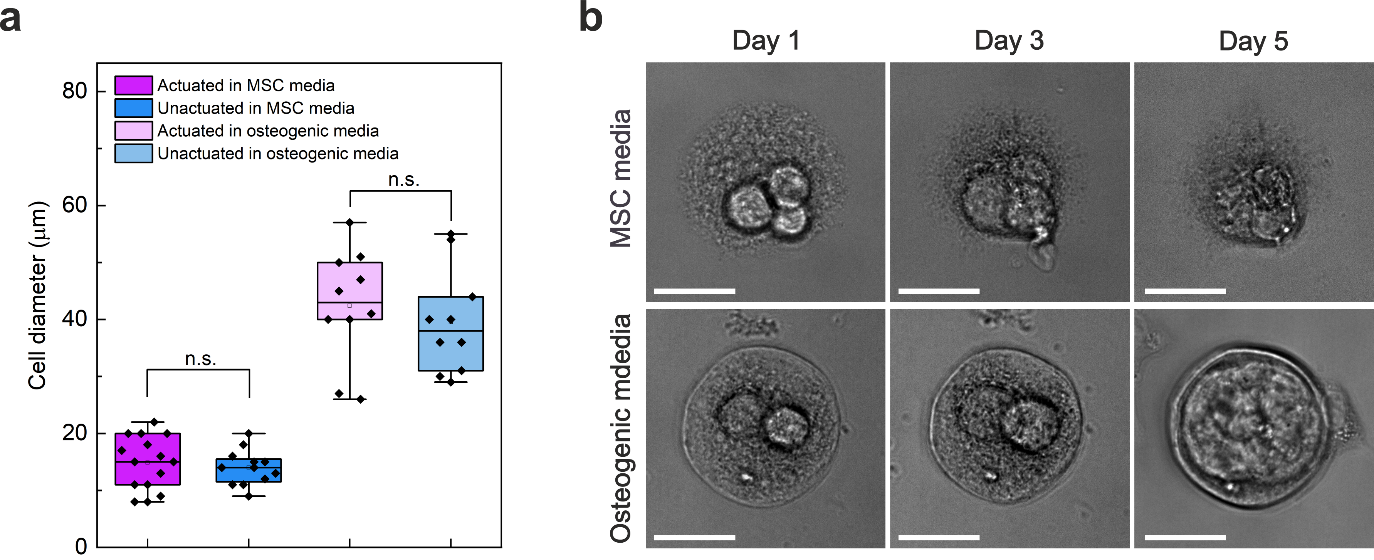


**Figure S26.** a) Changes in the diameter of encapsulated cells in microgels in either DMEM-based regular cell culture media or osteogenic media. b) Representative bright-field image series of cell-laden microgels illustrate cell diameter changes in the two media on Day 1 before mechanical stimulation, Day 3 before mechanical stimulation, and Day 5 after fixation (scale bar: 30 µm).
